# Synthesis of Enantiomerically Pure Bambus[6]urils Utilizing Orthogonal Protection of Glycolurils

**DOI:** 10.1021/acs.joc.3c00667

**Published:** 2023-07-28

**Authors:** Petr Slávik, Jacopo Torrisi, Pia Jurček, Jan Sokolov, Vladimír Šindelář

**Affiliations:** † Department of Chemistry, Faculty of Science, Masaryk University, 625 00 Brno, Czech Republic; ‡ RECETOX, Faculty of Science, Masaryk University, 625 00 Brno, Czech Republic

## Abstract

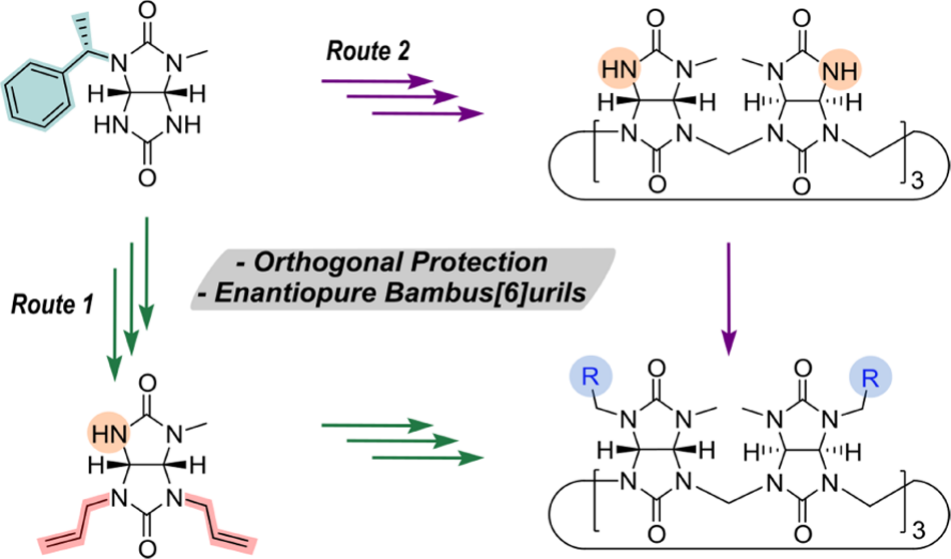

A general strategy for the synthesis of 2*N*,4*N*′-disubstituted glycoluril enantiomers on a multigram scale using orthogonal protection is reported. The use of these glycolurils is demonstrated in the synthesis of enantiomerically pure bambus[6]uril macrocycles. Moreover, the deprotection of (*S*)-1-phenylethyl substituents on the macrocycle was achieved, opening access to various chiral bambus[6]urils *via* post-macrocyclization modification strategy.

## Introduction

Chiral macrocycles play an important role among supramolecular host molecules because of their ability to specifically interact with chiral guest molecules. Selective and strong host–guest complex formation is due to a well-confined binding site of the macrocycle that offers a multivalent interaction with the guest. Thus, chiral macrocycles are often used for the differentiation, separation, and sensing of chiral compounds with great potential in pharmaceutical, material chemistry, and biology.^1,2^

Glycoluril and its derivatives are rigid heterocycles with a curved backbone that have been used as building blocks for supramolecular hosts^3−5^ such as molecular clips,^6^ tweezers,^7^ baskets,^8^ and three-dimensional capsules.^9,10^ The most widely used glycoluril-based hosts are barrel-shaped macrocyclic molecules: cucurbit[*n*]urils^4,11−13^ and bambus[*n*]urils.^14,15^ Preparation of chiral cucurbituril derivatives as racemates was only reported without further isolation of the corresponding enantiomers.^16−21^ Other closely related chiral macrocycles such as biotin[6]uril^22^ and cyclohexylhemicucurbiturils^23−26^ were isolated as pure enantiomers, but their enantioselective binding of chiral carboxylates was demonstrated only in one case.^24^

Bambus[*n*]urils are macrocyclic molecules consisting of *n* alternating 2*N*,4*N′-*disubstituted glycoluril units connected by one row of methylene bridges. Six-membered bambusurils, bambus[6]urils, form stable inclusion complexes with various inorganic anions in which a single anion is usually positioned in their electron-positive cavity, further stabilized by hydrogen bonds with hydrogen methine atoms of glycoluril constitutional units. Bambus[6]urils show high association constants ranging up to 10^11^ M^–1^ in organic solvents.^27^ They function as efficient anion transporters,^27^ supramolecular hydrogels,^28^ as rotaxane constituents,^29^ and in the selective recognition of diacyanoaurate(I).^30^

**Scheme 1:**
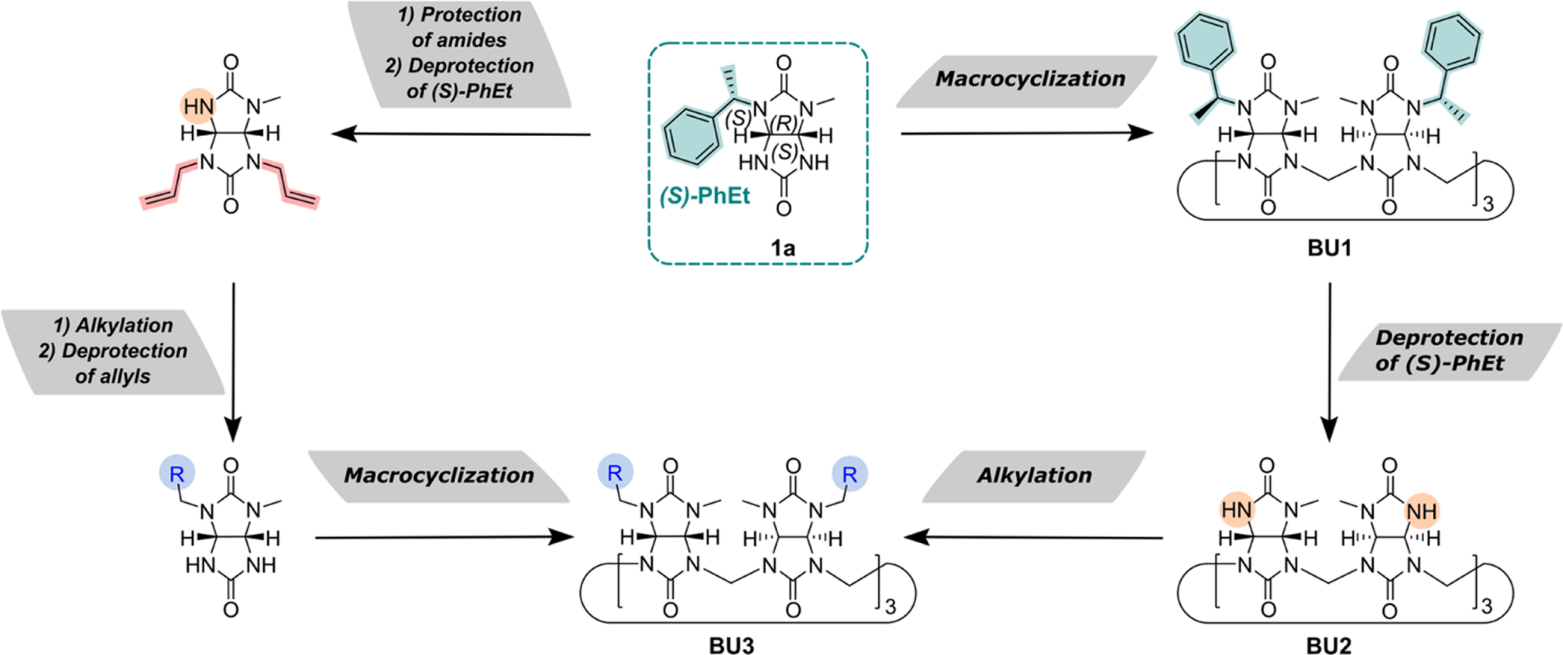
Schematic Overview of the Preparation of Enantiomerically Pure Bambus[6]uril **BU3***via* Orthogonal Protective Steps of **1a** and Post-Macrocyclization Modification of **BU1**

Majority of bambusurils are achiral.^15^ However, the synthesis of the first chiral bambusurils was recently reported, and their ability to bind enantiomers of biologically relevant compounds with selectivity exceeding 3 was demonstrated.^31^ The starting monomers for the synthesis of enantiomerically pure bambusurils are 2*N*,4*N*′-disubstituted glycolurils, bearing two different substituents, which are produced as a mixture of two stereoisomers. When a racemic mixture of glycolurils was used for the bambus[4]urils synthesis, a mixture of macrocycle stereoisomers was obtained, from which chiral macrocycles were separated by time-consuming and expensive high-performance liquid chromatography with chiral stationary phase.^32^ On the other hand, the use of a single stereoisomer in the macrocyclization reaction resulted in the selective preparation of enantiomerically pure bambusuril.^33^ Thus, isolation of a single glycoluril stereoisomer from the mixture is highly beneficial prior to the macrocyclization. When both substituents of 2*N*,4*N*′-disubstituted glycolurils are achiral, the glycolurils are produced as a racemic mixture from which isolation of a pure enantiomer can be challenging. This is why our previously reported approach was based on the preparation of 2*N*,4*N*′-disubstituted glycoluril **1** bearing (*S*)-phenylethyl and methyl substituents.^31^ As a consequence, glycoluril **1** was prepared as a mixture of diastereomers **1a** and **1b**. A single stereoisomer **1a** (for structure, see Scheme 1) was separated on a multigram scale from the mixture based on its different solubility in methanol and isopropanol. Simple preparation and isolation lacking chromatography purification and the use of relatively inexpensive starting material, (*S*)-1-phenylethylamine, make glycoluril **1a** ideal for the preparation of chiral bambus[6]uril **BU1** on gram scale. (*S*/*R*)-1-Phenylethylamine is used in diastereoselective additions of nucleophiles^34−36^ mainly as chiral auxiliary,^37^ which can be removed by hydrogenolysis^36^ or by using organic acids.^38−40^ If such a deprotection is possible on glycolurils, we may use it in conjunction with **1a** in the synthesis of a wide variety of chiral glycolurils and, consequently, the synthesis of chiral bambus[6]urils (**BU3**, Scheme 1). Deprotection of the (*S*/*R*)-1-phenylethyl group on bambus[6]uril **BU1** represents the alternative pathway to chiral bambus[6]urils (**BU3**). Here, we report our results on this line.

## Results and Discussion

**Scheme 2:**
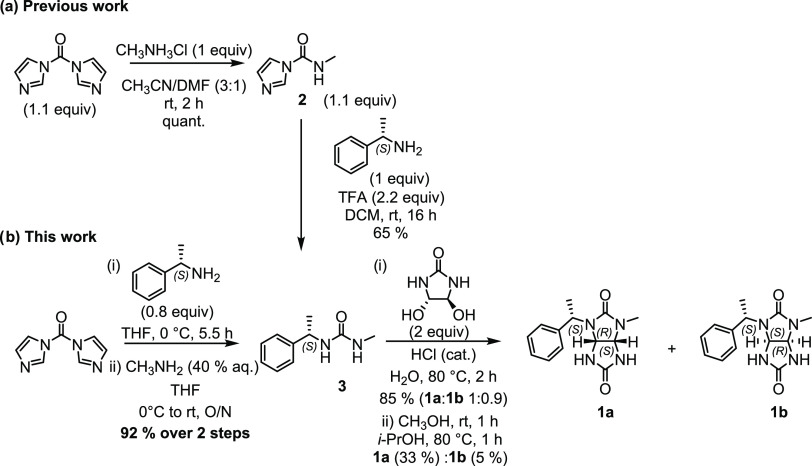
Synthesis of Glycolurils **1a** and **1b** (a) Previously reported synthesis route.^31,41^ (b) This work.

The previously described synthesis of glycoluril **1a** (Scheme 2a) had several drawbacks.^31^ The synthesis of chiral urea **3** starting from 1,1-carbonyldiimidazole (CDI) was time-consuming, as the intermediate *N*-methyl carbamoylimidazole **2** was obtained only after column chromatography purification.^41^ Furthermore, the reaction of **2** and (*S*)-1-phenylethylamine yielded urea **3** only in a moderate yield of 65%.^31^ Thus, we searched for an alternative approach. Inspired by a procedure reported by Padiya et al.,^42^ we prepared **3** in one pot by a two-step reaction employing CDI (Scheme 2b and Table S1). The reaction of CDI with (*S*)-1-phenylethylamine in THF resulted in **3** (65%), which was contaminated by undesired *N*,*N*′-bis((*S*)-1-phenylethyl)urea (35%). The formation of the side product was suppressed by slow addition of a dilute solution of (*S*)-1-phenylethylamine in THF using a syringe pump at 0 °C. In contrast to the slow addition of (*S*)-1-phenylethylamine, aqueous methylamine was added in one portion, followed by overnight stirring at room temperature. The resulting urea **3** was obtained with an overall yield of 92%.

Following a modified literature procedure,^31^ condensation of urea **3** with *trans*-4,5-dihydroxyimidazolidin-2-one afforded a mixture of diastereomers **1a** and **1b** (Scheme 2). For the following steps, we decided to use less soluble glycoluril **1a**, which was isolated from the glycoluril mixture by washing it with methanol and isopropanol.

**Scheme 3:**
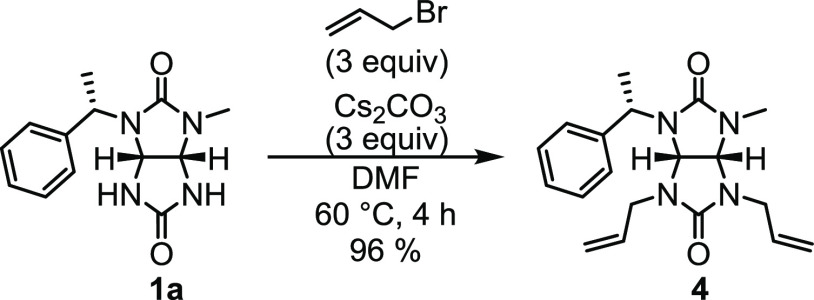
Protection of Nitrogen Atoms of Glycoluril **1a** with Allyl Bromide

Glycoluril **1a** was alkylated with allyl bromide in the presence of a base (Scheme 3, Table S2). We first tested sodium hydroxide to obtain glycoluril **4** in a yield of 58 and 68%. Later, we found that the reaction gives a better yield (96%) of **4** in the presence of cesium carbonate (Scheme 3). Two main reasons for the high yield of **4** were identified: (a) the cesium salt is a mild base and does not cause decomposition of both the starting material and the product, and (b) cesium salts can be removed from the reaction mixture by simple filtration.

**Scheme 4:**
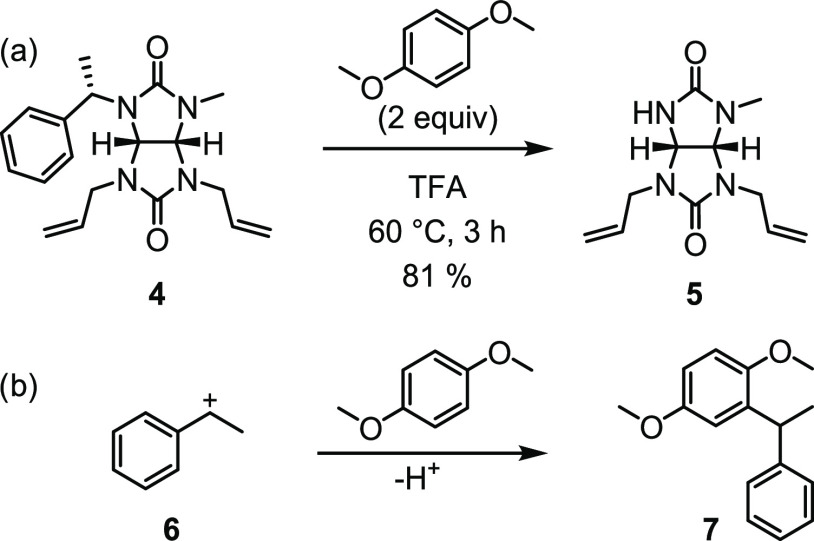
(a) Cleavage of the (*S*)-1-Phenylethyl Group and (b) Reaction of 1-Phenylethan-1-ylium Cation **6** with 1,4-Dimethoxybenzene

The allyl group was chosen as a suitable orthogonal protective group for the two NH positions of glycoluril **1a**, since it can withstand acidic conditions required for the deprotection of (*S*)-1-phenylethyl group. The removal of the (*S*)-1-phenylethyl group from glycoluril **4** was first tested by formic acid (Table S3).^38^ However, the long reaction time resulted in the decomposition of the starting material and the product. Better results were achieved using neat trifluoroacetic acid (TFA) at 60 °C, which yielded the desired product **5** (Scheme 4a, Table S3). However, TLC analysis of the reaction mixture showed the presence of multiple products. We hypothesized that it could be caused by an undesired reaction of cleaved 1-phenylethan-1-ylium cation **6**.^43^ Thus, we included a cation scavenger, 1,4-dimethoxybenzene, in the reaction, which resulted in a less complex reaction mixture and in an improvement of the yield of **5** from 70 to 81% (Scheme 4a). We were also able to isolate the byproduct **7** formed by the reaction of phenylethyl cation **6** and 1,4-dimethoxybenzene (Scheme 4b).^44^

**Scheme 5:**
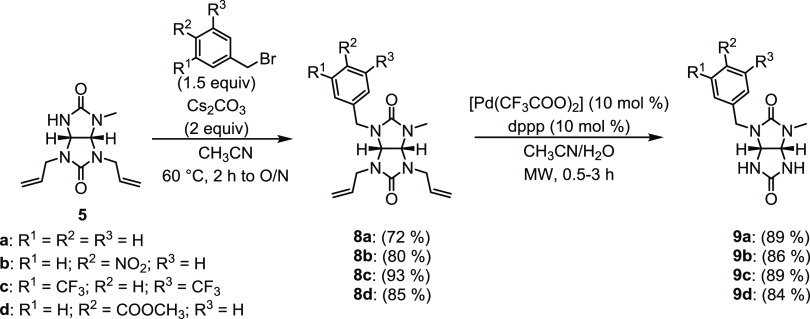
Alkylation of Glycoluril **5** followed by Cleavage of the Allyl Groups of **8a**–**8d**

Our next step was to alkylate the NH position of enantiomerically pure glycoluril **5** with benzyl derivatives bearing various substituents (H, NO_2_, CF_3_, COOCH_3_, Scheme 5). The reactions were carried out in CH_3_CN at 60 °C in the presence of cesium carbonate, yielding **8a–8d** in yields of 72–93%.^45^

The final step in the synthesis of 2*N*,4*N*′-disubstituted chiral glycolurils **9a–9d** was the deprotection of allyl groups on **8a–8d** (Scheme 5). Three different reaction conditions were tested using **8c** as a model compound (Table S4). The first attempt was inspired by Zacuto and Xu.^46^ RhCl_3_ and glycoluril **8c** were refluxed in anhydrous *n*-propanol for 16 h, yielding the desired glycoluril **9c** in a yield of 46% (Table S4). Next, we followed a modified procedure reported by Cadierno.^47^ The starting materials, ruthenium catalyst dichloro-[(2,6,10-dodecatriene)-1,12-diyl]ruthenium(IV) and KIO_4_, were heated at 80 °C in H_2_O/CH_3_CN mixture for 3 days, but the desired product was not detected. Lastly, we tested the conditions published by Ohmura.^48^ Glycoluril **8c**, palladium(II) trifluoroacetate, and 1,3-bis(diphenylphosphino)propane (dppp) were heated in H_2_O/CH_3_CN mixture at 60 °C (Scheme 5, Table S4). The reaction took 6 days to complete with an 85% yield of **9c**. We were able to reduce the reaction time to 30 min by performing the reaction in a closed vessel using a microwave reactor at 120 °C, obtaining **9c** in 89% yield. The latter procedure was used to convert glycolurils **8a–8d** into **9a–9d** with high yields of 84–89%. To demonstrate the potential of prepared chiral glycolurils, we selected glycoluril **9a** and used it in the synthesis of bambusuril **BU3a**. The reaction was performed in dry dioxane in the presence of paraformaldehyde and a catalytic amount of sulfuric acid. The compound was isolated as **HSO**_**4**_^**–**^**@BU3a** in 46% yield.

**Scheme 6:**
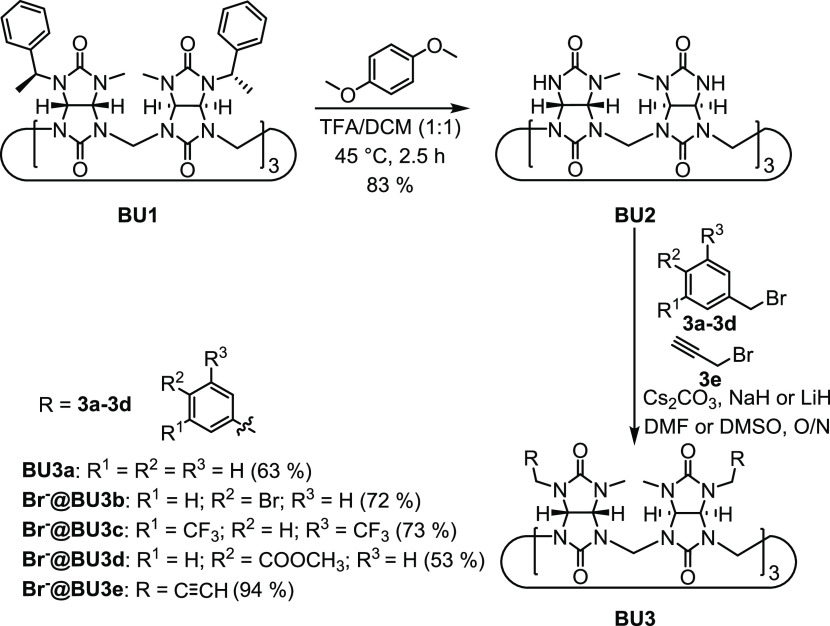
Cleavage of the (*S*)-1-Phenylethyl Group from **BU1** Resulting in **BU2** and Subsequent Alkylation of **BU2** into **BU3a–BU3e**

The successful deprotection of the (*S*)-1-phenylethyl group on glycoluril inspired us to investigate the deprotection of the same group in the case of bambusuril **BU1** (Scheme 6). Anion free macrocycle **BU1** was previously prepared in our group in 14% yield.^31^ However, in this work, we were able to increase its yield to 61% by improving its isolation and purification. Deprotection of **BU1** was performed in TFA/DCM (1:1) in the presence of 1,4-dimethoxybenzene at 45 °C for 2.5 h (Scheme 6). **BU2** was isolated in 83% yield.

To demonstrate that **BU2** can be further modified and used for the synthesis of various enantiomerically pure bambus[6]uril derivatives, **BU2** was alkylated with benzyl bromide derivatives or propargyl bromide(Scheme 6). The alkylation was carried out in dry DMF or DMSO in the presence of Cs_2_CO_3_, NaH, or LiH under an argon atmosphere overnight. Enantiomerically pure bambusurils **BU3a–BU3e** were isolated anion free or as bromide complexes in yields of 53–94%.

## Conclusions

Two general synthetic strategies to obtain enantiomerically pure bambus[6]urils were described (Scheme 1). Both routes utilized deprotection of (*S*)-1-phenylethyl group attached to glycoluril. The first route, derived from diastereomerically pure glycoluril **1a** using orthogonal protection and deprotection cycles of allyl and (*S*)-1-phenylethyl groups, resulted in several enantiomerically pure glycolurils **9a–9d**. *p*-Methoxybenzyl,^49,50^*tert*-butyloxycarbonyl,^49^ benzyl,^51^ and acetyl^51^ groups have been used in the protection of glycoluril’s nitrogen atoms. However, to the best of our knowledge, orthogonal (de)protection of glycolurils has not been reported to date. Glycoluril **9a** was further macrocyclized into enantiomerically pure bambus[6]uril **BU3a** to demonstrate the potential of these glycoluril derivatives. The second route leading to enantiomerically pure bambus[6]urils **BU3a–BU3e** was based on the deprotection of (*S*)-1-phenylethyl groups of **BU1** and subsequent alkylation of **BU2**. Both synthetic strategies allow straightforward access to a large library of enantiomerically pure bambus[6]uril macrocycles. However, the second route based on the deprotection of **BU1** is preferable over the first route for the bambus[6]uril synthesis as it comprises less synthetic steps and affords **BU3** in a slightly higher yield. The presence of functional groups such as bromobenzyl (**BU3b**) and propargyl (**BU3e**) allows further modification on the macrocycles by using, for example, cross-coupling and azide-alkyne Huisgen cycloaddition reactions.

## Experimental Section

### General

All reagents and solvents used were purchased from commercial suppliers and used without further purification. *trans*-4,5-Dihydroxyimidazolidin-2-one was synthesized based on a reported procedure.^52^ Reaction mixtures were heated on DrySyn heating blocks, and the reaction temperatures stated refer to the settings of the magnetic stirrer. Microwave syntheses were performed in pressurized sealed Discovered SP vessels closed with Activent caps. MW material was purchased from CEM. The Dynamic Control method was used for all microwave reactions, where the temperature and the pressure were set (*P* = 150 W max, *T* = 120 °C, 300 PSI max). Reactions were monitored by thin-layer chromatography (TLC) using aluminum plates precoated with silica gel (60 F_254_, Merck) impregnated with a fluorescent indicator. TLC plates were visualized with ultraviolet light (λ = 254 nm) and by staining with aqueous potassium permanganate (KMnO_4_) or ceric ammonium molybdate (CAM), followed by heating. Flash column chromatography was performed using silica gel (60 Å, 40–63 μm, Fluorochem) or CombiFlash NextGen 300 from Teledyne ISCO. NMR spectra were recorded on a Bruker Avance III HD 500 and Avance III 300 MHz spectrometer equipped with a BBFO probe with working frequency 500 MHz or 300 MHz for ^1^H, 126 MHz for ^13^C{^1^H}, and 471 or 282 MHz for ^19^F{^1^H}. All experiments were recorded at 303.15 K. NMR chemical shifts (δ) are reported in parts per million (ppm) using a residual solvent signal as a reference for the measured spectra in DMSO-*d*_6_ (^1^H = 2.50, ^13^C = 39.52) and CD_3_CN (^1^H = 1.94, ^13^C = 1.32). ^19^F NMR spectra were not referenced. Multiplicities are reported as singlet (s), doublet (d), doublet of doublets (dd), doublet of doublet of triplets (ddt), doublet of quartets (dq), triplet (t), quartet (q), multiplet (m), and broad (br). Signals were assigned with the aid of ^1^H-^1^H COSY, ^1^H-^13^C HSQC, and ^1^H-^13^C HMBC experiments. High-resolution mass spectra (HRMS) were obtained on Agilent 6224 accurate-mass time-of-flight (TOF) mass spectrometer. Samples were ionized by electrospray ionization (ESI) or atmospheric pressure chemical ionization (APCI). Matrix-assisted laser desorption ionization with detection of time-of-flight (MALDI-TOF) mass spectra were measured on the MALDI-TOF MS UltrafleXtreme (Bruker Daltonics). Samples were ionized by Nd-YAG laser (355 nm) from 2,5-dihydroxybenzoic acid (DHB) matrix. Melting points were measured on a Stuart SMP40 melting point apparatus.

### Glycolurils **1a** and **1b**

The reaction procedure for separation of diastereomers was modified from a previously published procedure.^31^ Urea **1** (7.68 g, 43.09 mmol, 1.0 equiv) and *trans*-4,5-dihydroxyimidazolidin-2-one (10.15 g, 85.95 mmol, 2.0 equiv) were weighed into a 250 mL round-bottom flask. Water (70 mL) was added, and the mixture was heated to 80 °C. After 15 min, HCl (10%; 2 mL) was added, and heating was continued at 80 °C. The solids gradually dissolved, and a white precipitate emerged. After 2 h, the reaction mixture was cooled to 0 °C, and the resulting solid was isolated by filtration, washed with water (2 × 25 mL), and dried *in vacuo*, yielding a mixture of diastereomers (9.50 g; 85%; **1a/1b** 1:0.9). *R*_*f*_ = 0.33 (DCM/CH_3_OH 9:1; UV, KMnO_4_). The mixture of diastereomers (9.50 g) was suspended in CH_3_OH (60 mL), and the mixture was stirred at room temperature for 1 h. Solid was isolated by filtration and dried *in vacuo*. The solid was then suspended in *i*-PrOH (30 mL) and stirred at 80 °C for 1 h, and the resulting solid was isolated by filtration. The filtrate was left to stand at room temperature, and more precipitate was collected and dried *in vacuo*. The solids were combined, yielding the less soluble diastereomer **1a** as a white solid (3.70 g; 33%). The methanolic filtrate was evaporated under reduced pressure to give a white solid, which was then recrystallized from boiling water (190 mL). Crystals were collected by filtration and dried *in vacuo* to give a mixture of diastereomers, which could be separated again. The aqueous filtrate was evaporated under reduced pressure to give a white solid, which was recrystallized from boiling *i*-PrOH (25 mL) to give the more soluble diastereomer **1b** (561 mg; 5%). The spectroscopic data correspond to the literature.^31^

### Urea **3**

1,1′-Carbonyldiimidazole (10.50 g, 64.75 mmol, 1.0 equiv) was weighed into a 250 mL round-bottom flask equipped with a stir bar and septum. The flask was flushed with argon, and THF (80 mL, precooled) was added by a syringe. The resulting white suspension was cooled to 0 °C while stirring the mixture vigorously. Solution of (*S*)-1-phenylethylamine (6.03 g, 49.76 mmol, 0.8 equiv) in THF (80 mL) was added to the reaction mixture by a syringe pump (20 mm diameter syringe with a volume of 20 mL, addition rate 300 μL min^–1^). The reaction mixture was cooled the whole time. The reaction mixture gradually dissolved to give a yellow transparent solution. After the addition was finished (5 h), the reaction was stirred for another 30 min to complete the transformation to intermediate. Methylamine (40% aq.; 6.00 g, 77.27 mmol, 1.2 equiv) was added to the reaction mixture in one portion at 0 °C, and the resulting yellow solution was stirred overnight, allowing the temperature to grow gradually to room temperature. The reaction mixture was evaporated under reduced pressure to give a yellow oily liquid, which solidified upon standing. The crude was diluted with HCl (10%; 50 mL), and a sticky white precipitate emerged. The mixture was extracted with DCM (3 × 50 mL); the combined organic layers were washed with HCl (10%; 25 mL) and brine (50 mL) and dried over anhydrous magnesium sulfate. The drying agent was filtered off, and the filtrate was evaporated under reduced pressure to give a white solid (8.18 g, 92%). The analytical sample was obtained by recrystallization from water. The spectroscopic data correspond to the literature.^31^*M*_p_: 104–106 °C; *R*_*f*_ = 0.27 (DCM/CH_3_OH 19:1; UV, KMnO_4_). ^1^H NMR (500 MHz, DMSO-*d*_6_): δ 7.33–7.25 (m, 4H), 7.23–7.16 (m, 1H), 6.30 (d, *J* = 8.2 Hz, 1H), 5.63 (q, *J* = 4.8 Hz, 1H), 4.77–4.68 (m, 1H), 2.53 (d, *J* = 4.6 Hz, 3H), 1.30 (d, *J* = 6.9 Hz, 3H). ^13^C{^1^H} NMR (126 MHz, DMSO-*d*_6_): δ 157.8, 145.8, 128.1, 126.3, 125.7, 48.6, 26.2, 23.3. HRMS (APCI+) *m*/*z*: [M + H]^+^ Calcd for C_10_H_15_N_2_O 179.1179; found: 179.1181.

### Glycoluril **4**

Glycoluril **1a** (2.59 g, 9.95 mmol, 1.0 equiv) and Cs_2_CO_3_ (9.82 g, 30.14 mmol, 3.0 equiv) were weighed into a 100 mL round-bottom flask equipped with a stir bar and septum. The flask was flushed with argon. Dry DMF (40 mL) was added, and the resulting white suspension was heated at 60 °C for 1 h. Allyl bromide (3.65 g, 30.17 mmol, 3.0 equiv) was added dropwise over 30 min. The resulting off-white suspension was stirred at 60 °C. TLC analysis after 4 h indicated the disappearance of the starting material. The reaction mixture was filtered through a Celite pad, and the pad was washed with additional DMF (20 mL). The clear yellow filtrate was evaporated under reduced pressure to give a dark orange oily liquid. The crude product was purified by column chromatography (SiO_2_, DCM/CH_3_OH 40:1) to give a white solid (3.25 g, 96%). *M*_p_: 89–91 °C; *R*_*f*_ = 0.72 (DCM/CH_3_OH 9:1; UV, KMnO_4_). ^1^H NMR (500 MHz, DMSO-*d*_6_): δ 7.37–7.26 (m, 5H), 5.78 (m, 1H), 5.52 (m, 1H), 5.23–5.16 (m, 2H), 5.16 (d, *J* = 8.5 Hz, 1H), 5.12 (d, *J* = 8.5 Hz, 1H), 5.00 (dq, *J* = 10.4, 1.4 Hz, 1H), 4.87–4.78 (m, 2H), 3.98 (m, 2H), 3.81 (dd, *J* = 16.4, 6.2 Hz, 1H), 3.26 (dd, *J* = 16.2, 7.2 Hz, 1H), 2.80 (s, 3H), 1.58 (d, *J* = 7.0 Hz, 3H). ^13^C{^1^H} NMR (126 MHz, DMSO-*d*_6_): δ 158.1, 141.0, 133.8, 132.8, 128.3, 126.9, 126.7, 117.46, 116.92, 70.26, 67.70, 52.82, 45.10, 44.53, 29.90, 18.84. HRMS (APCI+) *m*/*z*: [M + H]^+^ Calcd for C_19_H_25_N_4_O_2_: 341.1972; found: 341.1975. Optical rotation: [α]_589_^23^ = 17.3° (*c* = 1.25 g/100 mL, MeOH)

### Glycoluril **5**

Glycoluril **4** (5.24 g, 15.39 mmol, 1.0 equiv) and 1,4-dimethoxybenzene (4.26 g, 30.83 mmol, 2.0 equiv) were weighed into a 100 mL round-bottom flask. TFA (15 mL) was added, and the resulting clear brown solution was stirred at 60 °C. TLC analysis (EtOAc) after 3 h indicated the disappearance of the starting material. The reaction mixture was cooled to 0 °C, and the reaction mixture was basified with saturated sodium carbonate solution (55 mL), strong gas evolution was observed, and the pH level reached 8–9. The resulting mixture was extracted with DCM (3 × 50 mL); the combined organic layers were washed with brine and dried over anhydrous magnesium sulfate. The drying agent was filtered off, and the filtrate was evaporated under reduced pressure to give a dark orange oil, which solidified upon standing. The crude product was purified by column chromatography (SiO_2_, DCM/CH_3_OH 20:1) to give an off-white solid (2.96 g, 81%). *M*_p_: 90–93 °C; *R*_*f*_ = 0.15 (EtOAc; KMnO_4_). ^1^H NMR (500 MHz, DMSO-*d*_6_): δ 7.61 (s, 1H), 5.83–5.68 (m, 2H), 5.23– 5.12 (m, 6H), 3.98 (dd, *J* = 16.4, 5.0 Hz, 1H), 3.93 (dd, *J* = 15.9, 4.8 Hz, 1H), 3.77 (dd, *J* = 16.4, 6.2 Hz, 1H), 3.54 (dd, *J* = 16.0, 6.8 Hz, 1H), 2.74 (s, 3H). ^13^C{^1^H} NMR (126 MHz, DMSO-*d*_6_): δ 159.6, 157.2, 134.1, 133.1, 117.3, 116.9, 71.4, 63.5, 45.0, 43.1, 29.4. HRMS (APCI+) *m*/*z*: [M + H]^+^ Calcd for C_11_H_17_N_4_O_2_ 237.1346; found: 237.1349. Optical rotation: [α]_589_^23^ = 17.4° (*c* = 1.22 g/100 mL, MeOH)

### General Procedure for the Alkylation of Glycoluril **5**

Glycoluril **5** (4.32 mmol, 1.0 equiv, 0.43 M) and Cs_2_CO_3_ (8.62 mmol, 2.0 equiv, 0.86 M) were weighed into a 50 mL round-bottom flask equipped with a stir bar and septum. The flask was flushed with argon, and CH_3_CN (10 mL) was added. The resulting off-white suspension was stirred at 60 °C for 1 h under an argon atmosphere. Benzyl bromide derivative (6.48 mmol, 1.5 equiv, 1.3 M) in CH_3_CN (5 mL) was added to the reaction mixture dropwise over 40 min. The resulting mixture was stirred at 60 °C. TLC analysis (DCM/CH_3_OH) indicated the disappearance of the starting material. The reaction mixture was filtered through a Celite pad and was washed with additional CH_3_CN (10 mL). The filtrate was evaporated under reduced pressure, and the crude was purified by column chromatography.

#### Glycoluril **8a**

Alkylation was performed based on the general procedure (reaction time: overnight). White solid; 150 mg (72%, calculated yield) from 150 mg (0.63 mmol) of **5** in CH_3_CN (2 mL); SiO_2_, gradient from DCM to DCM/CH_3_OH 99:1 as eluent. ^1^H NMR (500 MHz, DMSO-*d*_6_): δ 7.38–7.19 (m, 5H), 5.84–5.73 (m, 1H), 5.69–5.57 (m, 1H), 5.23–5.19 (m, 3H), 5.11–5.05 (m, 2H), 5.01 (dq, *J* = 17.1, 1.7 Hz, 1H), 4.54 (d, *J* = 16.3 Hz, 1H), 4.32 (d, *J* = 16.2 Hz, 1H), 4.06–3.80 (m, 3H), 3.40 (ddt, *J* = 16.2, 6.7, 1.3 Hz, 1H), 2.84 (s, 3H). ^13^C{^1^H} NMR (126 MHz, DMSO-*d*_6_): δ 158.6, 157.8, 137.5, 133.9, 133.4, 128.5, 127.2, 127.1, 117.2, 116.9, 70.1, 67.7, 46.1, 45.2, 44.9, 30.1. HRMS (APCI+) *m*/*z*: [M + H]^+^ Calcd for C_18_H_22_N_4_O_2_: 327.1816; found: 327.1815. [α]_589_^23^ = −18.4° (*c* = 0.60 g/100 mL, MeOH).

#### Glycoluril **8b**

Alkylation was performed based on the general procedure (reaction time: 4 h). Orange solid; yield 1.28 g (80%) from 1.02 g (4.32 mmol) of **5** in CH_3_CN (15 mL); SiO_2_, DCM/CH_3_OH 45:1 as eluent; *M*_p_: 77–78 °C (decomp.); *R*_*f*_ = 0.51 (DCM/CH_3_OH 19:1; UV, KMnO_4_). ^1^H NMR (500 MHz, DMSO-*d*_6_): δ 8.21 (d, *J* = 8.4 Hz, 2H), 7.50 (d, *J* = 8.4 Hz, 2H), 5.85–5.74 (m, 1H), 5.68–5.57 (m, 1H), 5.26–5.16 (m, 4H), 5.11–4.98 (m, 2H), 4.58 (d, *J* = 17.1 Hz, 1H), 4.53 (d, *J* = 17.1 Hz, 1H), 4.04–3.96 (m, 1H), 3.94–3.80 (m, 2H), 3.38 (dd, *J* = 16.3, 6.7 Hz, 1H), 2.85 (s, 3H). ^13^C{^1^H} NMR (126 MHz, DMSO-*d*_6_): δ 158.6, 157.7, 146.7, 146.0, 133.8, 133.3, 128.1, 123.6, 117.3, 117.0, 70.3, 68.3, 46.0, 45.2, 45.0, 30.2. HRMS (APCI+) *m*/*z*: [M + H]^+^ Calcd for C_18_H_22_N_5_O_4_: 372.1666; found: 372.1666. [α]_589_^23^ = −13.9° (*c* = 1.19 g/100 mL, MeOH)

#### Glycoluril **8c**

Alkylation was performed based on the general procedure (reaction time: 2 h). White solid; yield 4.11 g (93%) from 2.26 g (9.57 mmol) of **5** in CH_3_CN (25 mL); SiO_2_, DCM/CH_3_OH 60:1 as eluent; *M*_p_: 114–117 °C; *R*_*f*_ = 0.54 (DCM/CH_3_OH 19:1; UV, KMnO_4_). ^1^H NMR (500 MHz, DMSO-*d*_6_): δ 8.02 (s, 1H), 7.93 (s, 2H), 5.79 (m, 1H), 5.57 (m, 1H), 5.26–5.14 (m, 4H), 5.03–4.91 (m, 2H), 4.61 (s, 2H), 3.99 (ddt, *J* = 16.5, 4.8, 1.6 Hz, 1H), 3.93–3.80 (m, 2H), 3.43 (ddt, *J* = 16.3, 6.6, 1.4 Hz, 1H), 2.84 (s, 3H). ^13^C{^1^H} NMR (126 MHz, DMSO-*d*_6_): δ 158.7, 157.8, 141.8, 133.8, 133.4, 130.3 (q, *J* = 32.8 Hz), 127.9, 122.8 (q, *J* = 272.7 Hz), 120.9, 116.9, 116.8, 70.4, 68.6, 45.8, 45.1, 45.0, 30.1. ^19^F{^1^H} NMR (471 MHz, DMSO-*d*_6_): δ −61.35. HRMS (APCI+) *m*/*z*: [M + H]^+^ Calcd for C_20_H_21_F_6_N_4_O_2_: 463.1563; found: 463.1566. [α]_589_^23^ = 7.6° (*c* = 1.25 g/100 mL, MeOH)

#### Glycoluril **8d**

Alkylation was performed based on the general procedure (reaction time: 3 h). Yellow wax; yield 1.22 g (85%) from 876 mg (3.71 mmol) of **5** in CH_3_CN (20 mL); SiO_2_, DCM/CH_3_OH 50:1 as eluent; *R*_*f*_ = 0.47 (DCM/CH_3_OH 19:1; UV, KMnO_4_). ^1^H NMR (500 MHz, DMSO-*d*_6_): δ 7.94 (d, *J* = 8.3 Hz, 2H), 7.37 (d, *J* = 8.3 Hz, 2H), 5.82–5.76 (m, 1H), 5.62-5.57 (m, 1H), 5.24–5.17 (m, 2H), 5.22 (d, *J* = 8.5 Hz, 1H), 5.15 (d, *J* = 8.5 Hz, 1H), 5.06 (dd, *J* = 10.3, 1.5 Hz, 1H), 4.99 (dd, *J* = 17.2, 1.7 Hz, 2H), 4.55 (d, *J* = 16.8 Hz, 1H), 4.45 (d, *J* = 16.8 Hz, 1H), 3.99 (dd, *J* = 16.5, 4.9 Hz, 1H), 3.89–3.84 (m, 2H), 3.85 (s, 3H), 3.35 (dd, *J* = 16.2, 6.7, 1.4 Hz, 1H), 2.85 (s, 3H). ^13^C{^1^H} NMR (126 MHz, DMSO-*d*_6_): δ 166.0, 158.6, 157.7, 143.4, 133.9, 133.3, 129.4, 128.5, 127.3, 117.3, 116.9, 70.2, 68.0, 52.0, 46.1, 45.2, 44.9, 30.2. HRMS (APCI+) *m*/*z*: [M + H]^+^ Calcd for C_20_H_24_N_4_O_4_: 385.1870; found: 385.1868; [α]_589_^23^ = 19.1° (*c* = 1.48 g/100 mL, MeOH).

### General Procedure for Deprotection of Allyl Groups

Pd(CF_3_COO)_2_ (0.1 equiv) and dppp (0.1 equiv) were added to a microwave (MW) vial with a stir bar, and the solids were flushed with argon. CH_3_CN (2.0 mL) and water (1.6 mL) were added to the solids, and the resulting solution was stirred for 10 min at room temperature. Allyl-protected glycoluril (**8a–8d**) (3.0 mmol, 1 equiv) in CH_3_CN (5.0 mL) was then added to the MW vial. The resulting solution was irradiated for 0.5–3 h. TLC analysis (DCM/CH_3_OH) indicated the disappearance of the starting material. The reaction mixture was filtered through a cotton wool, which was further washed with CH_3_CN (15 mL). The clear filtrate was evaporated under reduced pressure and further purified by column chromatography.

#### Glycoluril **9a**

Deprotection of allyl groups was performed based on the general procedure. Irradiation parameters: 120 °C, 150 W max, 300 PSI max, medium stirring, 0.5 h. White solid; yield 100 mg (89%) from 150 mg (0.46 mmol) of **8a** in CH_3_CN (3 mL) and H_2_O (330 μL); SiO_2_, DCM/CH_3_OH 50:1 as eluent. This compound was previously reported as a part of a racemic mixture with second enantiomer.^32^^1^H NMR (500 MHz, DMSO-*d*_6_): δ 7.56 (m, 1H), 7.37–7.22 (m, 5H), 5.15 (dd, *J* = 8.0, 1.8 Hz, 1H), 5.02 (dd, *J* = 8.0, 1.8 Hz, 1H), 4.58 (d, *J* = 15.6 Hz, 1H), 4.00 (d, *J* = 15.6 Hz, 1H), 2.69 (s, 3H). ^13^C{^1^H} NMR (126 MHz, DMSO-*d*_6_): δ 161.0, 157.4, 137.4, 128.4, 127.7, 127.1, 67.2, 65.0, 43.9, 27.7. HRMS (APCI+) *m*/*z*: [M + H]^+^ Calcd for C_12_H_14_N_4_O_2_: 247.1190; found: 247.1192. [α]_589_^23^ = −24.0° (*c* = 0.78 g/100 mL, MeOH).

#### Glycoluril **9b**

Deprotection of allyl groups was performed based on the general procedure. Irradiation parameters: 120 °C, 150 W max, 300 PSI max, medium stirring, 0.5 h. Off-white foamy solid; yield 431 mg (86%) from 639 mg (1.72 mmol) of **8b** in CH_3_CN (4 mL) and H_2_O (1.2 mL); SiO_2_, DCM/CH_3_OH 10:1 as eluent; *M*_p_: 110 °C (decomp.); *R*_*f*_ = 0.23 (DCM/CH_3_OH 9:1; UV, CAM). ^1^H NMR (500 MHz, DMSO-*d*_6_): δ 8.21 (d, *J* = 8.6 Hz, 2H), 7.59 (s, 1H), 7.52 (d, *J* = 8.7 Hz, 2H), 7.50 (s, 1H), 5.19 (dd, *J* = 8.0, 1.7 Hz, 1H), 5.13 (dd, *J* = 8.1, 1.9 Hz, 1H), 4.61 (d, *J* = 16.4 Hz, 1H), 4.24 (d, *J* = 16.3 Hz, 1H), 2.70 (s, 3H). ^13^C{^1^H} NMR (126 MHz, DMSO-*d*_6_): δ 160.8, 157.5, 146.7, 145.9, 128.6, 123.5, 67.4, 65.5, 43.8, 27.7. HRMS (APCI+) *m*/*z*: [M + H]^+^ Calcd for C_12_H_14_N_5_O_4_: 292.1040; found: 292.1038. [α]_589_^23^ = −46.5° (*c* = 1.20 g/100 mL, MeOH)

#### Glycoluril **9c**

Deprotection of allyl groups was performed based on the general procedure. Irradiation parameters: 120 °C, 150 W max, 300 PSI max, medium stirring, 0.5 h. Off-white foamy solid; yield 144 mg (89%) from 194 mg (0.42 mmol) of **8c** in CD_3_CN (1 mL) and D_2_O (0.3 mL); SiO_2_, DCM/CH_3_OH 10:1 as eluent; *M*_p_: 99–101 °C (decomp.); *R*_*f*_ = 0.07 (DCM/CH_3_OH 19:1; UV, CAM). ^1^H NMR (500 MHz, DMSO-*d*_6_): δ 8.00 (s, 1H), 7.94 (s, 2H), 7.60 (s, 1H), 7.48 (s, 1H), 5.23–5.15 (m, 2H), 4.60 (d, *J* = 16.4 Hz, 1H), 4.33 (d, *J* = 16.4 Hz, 1H), 2.71 (d, *J* = 1.2 Hz, 3H). ^13^C{^1^H} NMR (126 MHz, DMSO-*d*_6_): δ 160.8, 157.6, 141.6, 130.2 (q, *J* = 32.7 Hz), 128.4 (d, *J* = 4.0 Hz), 122.8 (q, *J* = 273.4 Hz), 120.9, 67.52, 65.7, 43.9, 27.8. ^19^F{^1^H} NMR (471 MHz, DMSO-*d*_6_): δ −61.21. HRMS (APCI+) *m*/*z*: [M + H]^+^ Calcd for C_14_H_13_F_6_N_4_O_2_: 383.0937; found: 383.0935. [α]_589_^23^ = 16.5° (*c* = 1.04 g/100 mL, MeOH)

#### Glycoluril **9d**

Deprotection of allyl groups was performed based on the general procedure. Irradiation parameters: 140 °C, 200 W max, 300 PSI max, medium stirring, 3 h. Off-white foamy solid; yield 753 mg (84%) from 1.14 g (2.95 mmol) of **8d** in CD_3_CN (7 mL) and H_2_O (1.6 mL); SiO_2_, DCM/CH_3_OH 10:1 as eluent; *M*_p_: 88–90 °C; *R*_*f*_ = 0.26 (DCM/CH_3_OH 9:1; UV, CAM). ^1^H NMR (500 MHz, DMSO-*d*_6_): δ 7.93 (d, *J* = 8.3 Hz, 2H), 7.57 (s, 1H), 7.53 (s, 1H), 7.39 (d, *J* = 8.3 Hz, 2H), 5.18 (dd, *J* = 8.1, 1.8 Hz, 1H), 5.08 (dd, *J* = 8.0, 1.8 Hz, 1H), 4.60 (d, *J* = 16.0 Hz, 1H), 4.14 (d, *J* = 16.1 Hz, 1H), 3.84 (s, 3H), 2.70 (s, 3H). ^13^C{^1^H} NMR (126 MHz, DMSO-*d*_6_): δ 166.0, 160.9, 157.5, 143.3, 129.3, 128.5, 127.8, 67.3, 65.3, 52.0, 43.8, 27.7. HRMS (APCI+) *m*/*z*: [M + H]^+^ Calcd for C_14_H_16_N_4_O_4_: 305.1244; found: 305.1242; [α]23589 = 68.2° (*c* = 1.45 g/100 mL, MeOH).

### Macrocyclization

#### Bambus[6]uril **HSO**_**4**_^**–**^**@BU3a**

Glycoluril **9a** (100 mg, 0.41 mmol, 1 equiv) and paraformaldehyde (15 mg, 0.50 mmol, 1.2 equiv) were suspended in dry dioxane (2 mL) and heated to 80 °C. H_2_SO_4_ (60 μL; 2.9% v/v) was added to the hot solution, and the clear solution was stirred at 80 °C for 2 h. The mixture was cooled to room temperature, and Et_2_O was added to precipitate the crude. The filtrated solid was further sonicated in CHCl_3_, filtrated, and dried *in vacuo* to yield **HSO**_**4**_^**–**^**@BU3a** as a white solid (51 mg, 46%). ^1^H NMR (500 MHz, DMSO-*d*_6_) δ 7.33–6.97 (m, 30H), 5.61 (dd, *J* = 8.5 Hz, 6H), 5.43 (dd, *J* = 8.5 Hz, 6H), 5.08–4.66 (m, 12H), 4.57 (d, *J* = 16.2 Hz, 6H), 4.25 (s, 6H), 2.98 (s, 18H). ^13^C{^1^H} NMR (126 MHz, DMSO-*d*_6_) δ 159.1, 158.7, 139.2, 128.0, 126.6, 68.9, 68.6, 48.0, 46.9, 46.7, 30.6. HRMS (ESI+) *m*/*z*: [M + H]^+^ Calcd for C_78_H_85_N_24_O_12_: 1549.6773; found: 1549.6755. [α]_589_^23^ = −15.3° (*c* = 0.57 g/100 mL, MeOH)

#### Bambus[6]uril **BU1**

The reaction procedure was slightly modified from previously published procedure.^31^ Glycoluril **1a** (2.50 g, 9.6 mmol, 1 equiv) and paraformaldehyde (325 mg, 10.8 mmol, 1.1 equiv) were dissolved in a mixture of dry dioxane (50 mL) and H_2_SO_4_ (1.4 mL; 2.7% v/v). The mixture was stirred at 80 °C for 80 min, after which the reaction mixture was allowed to cool to room temperature. Solid was collected by filtration, washed with dioxane (5 mL) and Et_2_O (2 × 25 mL), and dried *in vacuo*. Dried solid crude was dissolved in methanol (20 mL). Water (30 mL) and 25% ammonia (10 mL) were added, which resulted in precipitation of a white solid. The suspension was stirred at 80 °C overnight, after which it was cooled to room temperature and filtered. The collected solid was dissolved in DCM (25 mL) and sonicated for 10 min. The mixture was filtered through filter paper with fine pores to remove insoluble impurities. The filtrate was evaporated under reduced pressure to half of its volume when methanol (10 mL) was added. Evaporation under reduced pressure was continued to give **BU1** as a white solid (1.60 g, 61%). All data correspond to those in the literature.^31^

#### Bambus[6]uril **BU2**

Bambus[6]uril **BU1** (1.0 g, 0.61 mmol, 1 equiv) and 1,4-dimethoxybenzene (761 mg, 5.51 mmol, 9 equiv) were flushed with argon and dissolved in dry DCM (5 mL). TFA (5 mL) was added, and the solution was stirred at 45 °C for 2.5 h under an argon atmosphere. The solution was evaporated under reduced pressure and additionally co-evaporated with dichloromethane and methanol to dryness. The residue was suspended in ethanol (25 mL) and sonicated for 10 min, after which the suspension was centrifuged. The solid was washed with ethanol (2 × 4 mL) and Et_2_O (3 × 10 mL). The suspension was centrifuged after each wash to obtain **BU2** as a white solid (513 mg, 83%). *M*_p_: 356 °C (decomp.); ^1^H NMR (500 MHz, DMSO-*d*_6_) δ 7.82 (s, 6H), 5.22 (d, *J* = 8.2 Hz, 6H), 5.17 (d, *J* = 8.2 Hz, 6H), 4.82 (s, 6H), 4.66 (s, 6H), 2.96 (s, 18H). ^13^C{^1^H} NMR (126 MHz, DMSO-*d*_6_) δ 159.5, 158.0, 70.3, 63.6, 48.3, 46.4, 30.0. HRMS (ESI−) *m*/*z*: [M + I]^−^ Calcd for C_36_H_48_N_24_O_12_I: 1135.2934; found: 1135.2914. [α]_589_^23^ = −17.6° (*c* = 0.50 g/100 mL, MeOH)

### Alkylation of Bambus[6]uril BU2

#### Bambus[6]uril **BU3a**

Bambus[6]uril **BU2** (200 mg, 0.20 mmol, 1 equiv) and Cs_2_CO_3_ (600 mg, 1.84 mmol, 9.2 equiv) were dispersed in dry DMF (3.5 mL) under an argon atmosphere. Benzyl bromide (160 μL, 230 mg, 1.34 mmol, 6.7 equiv) was added, and the reaction mixture was stirred at room temperature for 18 h. The mixture was then filtered, and the reaction flask and the solids were washed with DMF (2 × 1 mL). DMF filtrate was evaporated to dryness, and the resulting solids were dispersed in Et_2_O (10 mL), collected by vacuum filtration, washed with Et_2_O (10 mL), and left to dry on the frit. The crude was treated with chloroform (10 mL) and sonicated for 5 min. The resulting suspension was filtered, and the solid was washed with chloroform (2 × 5 mL) and dried *in vacuo*, yielding **Br**^**–**^**@BU3a** (250 mg). The solid was dissolved in a mixture of CH_3_OH (4 mL) and DCM (2 mL). To the solution was added AgSbF_6_ (72 mg, 0.21 mmol) in CH_3_OH (3 mL), and the resulting suspension was stirred for 30 min. The solid was removed by filtration. The filtrate was concentrated *in vacuo* to approximately 3 mL, resulting in precipitation of a solid. The suspension was diluted with water (10 mL), and the solid was collected by filtration, washed with water (2 × 10 mL), and dried *in vacuo* yielding **BU3a** as a white solid (194 mg, 63%). ^1^H NMR (500 MHz, DMSO-*d*_6_) δ 7.27–7.14 (m, 30H), 5.23 (d, *J* = 8.3 Hz, 6H), 5.10 (d, *J* = 8.3 Hz, 6H), 4.84 (s, 6H), 4.64 (d, *J* = 16.3 Hz, 6H), 4.53 (d, *J* = 16.3 Hz, 6H), 4.16 (s, 6H), 3.05 (s, 18H). ^13^C{^1^H} NMR (126 MHz, DMSO-*d*_6_) δ 159.1, 158.2, 138.1, 128.3, 127.0, 126.8, 69.7, 68.8, 48.3, 47.7, 47.0, 31.0. HRMS (ESI+) *m*/*z*: [M + H]^+^ Calcd for C_78_H_85_N_24_O_12_: 1549.6773; found: 1549.6718. [α]_589_^23^ = −176.0° (*c* = 0.50 g/100 mL, MeOH).

#### Bambus[6]uril **Br**^**–**^**@BU3b**

NaH (60% dispersion in oil; 182 mg, 7.93 mmol, 24 equiv) was weighed into a Schlenk flask, and argon was flushed through for 5 min. Dry DMF (4.8 mL) was added at 0 °C. Subsequently, **BU2** (200 mg, 0.20 mmol, 1 equiv) was added in portions as a solid under an argon flow. The suspension was stirred at 0 °C for 1 h. 4-Bromobenzyl bromide (891 mg, 3.57 mmol, 18 equiv) was added in portions as a solid under an argon flow. The white mixture was stirred at 0 °C for 10 min, the cooling bath was removed, and the suspension was stirred at room temperature for 24 h. The reaction mixture was quenched with H_2_O (milli-Q; 1.5 mL) at 0 °C and stirred for another 10 min. EtOAc was added and transferred to a separating funnel. The organic layer was extracted with brine (5 × 10 mL) and dried over anhydrous Na_2_SO_4_. The solvent was removed under reduced pressure. The crude was dissolved in CH_3_OH/EtOAc (4:1) assisted by heating, and an excess of Et_2_O was added to precipitate the product **Br**^–^**@BU3b** as a white solid (300 mg, 72%). ^1^H NMR (500 MHz, CD_3_CN) δ 7.35 (d, *J* = 8.4 Hz, 12H), 7.18 (d, *J* = 8.1 Hz, 12H), 5.65 (d, *J* = 7.9 Hz, 6H), 5.46 (d, *J* = 8.5 Hz, 6H), 5.10 (s, 6H), 4.79 (d, *J* = 16.7 Hz, 6H), 4.53 (d, *J* = 16.7 Hz, 6H), 4.39 (s, 6H), 3.00 (s, 18H). ^13^C{^1^H} NMR (126 MHz, CD_3_CN) δ 160.0, 159.7, 140.0, 132.3, 129.5, 121.1, 69.4, 69.2, 49.9, 48.5, 47.9, 31.3. HRMS (ESI+) *m*/*z*: [M + Na]^+^ Calcd for C_78_H_78_Br_6_N_24_O_12_Na: 2045.1183; found: 2045.1125. [α]_589_^23^ = −14.6° (*c* = 0.54 g/100 mL, MeOH).

#### Bambus[6]uril **Br**^**–**^**@BU3c**

NaH (60% dispersion in oil; 48 mg, 1.2 mmol, 24 equiv) was weighed into a Schlenk flask, and argon was flushed through for 5 min. Dry DMF (1 mL) was added at 0 °C. Subsequently, **BU2** (50 mg, 0.05 mmol, 1 equiv) was added in portions as a solid under an argon flow. The suspension was stirred at 0 °C for 1 h. Then, a solution of 3,5-bis(trifluoromethyl)benzyl bromide (368 mg, 1.2 mmol, 24 equiv) in dry DMF (1 mL) was added dropwise over 0.5 h. The white mixture was stirred at 0 °C for 10 min, the cooling bath was removed, and the suspension was stirred overnight at room temperature. The reaction mixture was quenched with H_2_O (milli-Q; 5 mL) at 0 °C and stirred for another 10 min. The precipitate was filtered and washed with H_2_O (milli-Q). The crude was purified by flash column chromatography to yield **Br^–^@BU3c** as a yellow solid (89 mg, 73%). ^1^H NMR (500 MHz, CD_3_CN) δ 7.81 (m, 18H), 5.66 (d, *J* = 8.5 Hz, 6H), 5.56 (d, *J* = 8.5 Hz, 6H), 5.12 (s, 6H), 4.99 (d, *J* = 17.4 Hz, 6H), 4.68 (d, *J* = 17.1 Hz, 6H), 4.14 (s, 6H), 3.05 (s, 18H). ^13^C{^1^H} NMR (126 MHz, CD_3_CN) δ 160.2, 159.8, 144.1, 132.1 (q, *J* = 33.6 Hz), 128.0 (br), 124.5 (q, *J* = 272.0 Hz), 121.8(br), 69.8, 69.6, 49.9, 48.3, 48.1, 31.3. ^19^F{^1^H} NMR (282 MHz, CD_3_CN): δ −63.33. MALDI-TOF(+)MS *m*/*z*: [M + Na]^+^ Calcd for C_90_H_72_F_36_N_24_O_12_Na: 2387.508; found: 2387.568. [α]_589_^23^ = −23.4° (*c* = 0.55 g/100 mL, MeOH).

#### Bambus[6]uril **Br**^**–**^**@BU3d**

Bambus[6]uril **BU2** (100 mg, 0.10 mmol, 1 equiv) and Cs_2_CO_3_ (297 mg, 0.91 mmol, 9.1 equiv) were dispersed in dry DMF (2 mL) under an argon atmosphere. Methyl 4-(bromomethyl) benzoate (210 mg, 0.87 mmol, 8.7 equiv) was added, and the reaction mixture was stirred at room temperature for 24 h. The mixture was then filtered, and the reaction flask and the solid were washed with DMF (2 × 1 mL). Isolated solid was washed with Et_2_O (2 × 10 mL) and dried in a stream of air. The solid was poured into a mixture of water (10 mL) and acetic acid (1 mL) and sonicated for 5 min. The resulting suspension was filtered, and the isolated solid was washed with water (2 × 10 mL) and dried *in vacuo*. The compound was again washed with DMF (2 × 2 mL) and water (2 × 10 mL) and dried *in vacuo* yielding **Br**^**–**^**@BU3d** (120 mg, 53%). ^1^H NMR (300 MHz, DMSO-*d*_6_) δ 7.77 (d, *J* = 8.4 Hz, 12H), 7.28 (d, *J* = 8.3 Hz, 12H), 5.64 (d, *J* = 8.7 Hz, 6H), 5.47 (d, *J* = 8.5 Hz, 6H), 5.06 (s, 6H), 4.80 (d, *J* = 17.3 Hz, 6H), 4.59 (d, *J* = 17.2 Hz, 6H), 4.21 (s, 6H), 3.81 (s, 18H), 2.96 (s, 18H). All data correspond to those in the literature.^33^

#### Bambus[6]uril **Br**^**–**^**@BU3e**

**BU2** (50 mg, 0.05 mmol, 1 equiv) and LiH (9.6 mg, 1.2 mmol, 24 equiv) were weighed into a vial and flushed with argon. The vial was sealed with a septum and attached with an argon balloon. Dry DMSO-*d*_6_ was added (750 μL), and the reaction mixture was stirred at 40 °C for 1 h. Propargyl bromide solution (80% in toluene; 99 μL, 0.75 mmol, 15 equiv) was added dropwise to the mixture, and the solution was stirred at 40 °C for 24 h. The reaction mixture was quenched with H_2_O (milli-Q; 5 mL). The precipitate was filtered, washed with H_2_O (milli-Q), and dried *in vacuo* to yield **Br^–^@BU3e** as a brown solid (62 mg, 94%). *M*_p_: 310 °C (decomp.); ^1^H NMR (500 MHz, DMSO-*d*_6_) δ 5.75 (d, *J* = 8.6 Hz, 6H), 5.37 (d, *J* = 8.7 Hz, 6H), 5.05 (s, 6H), 5.00 (s, 6H), 4.43 (dd, *J* = 17.9, 2.4 Hz, 6H), 4.24 (dd, *J* = 17.9, 2.4 Hz, 6H), 3.02 (s, 18H), 2.66 (t, *J* = 2.4 Hz, 6H). ^13^C{^1^H} NMR (126 MHz, DMSO-*d*_6_) δ 158.3, 158.2, 80.3, 73.0, 68.5, 66.4, 48.6, 46.7, 33.7, 30.0. MALDI-TOF(-)MS *m*/*z*: [M + Br]^-^ Calcd for C_54_H_60_N_24_O_12_Br: 1315.401; found: 1315.407. [α]_589_^23^ = 14.4° (*c* = 0.69 g/100 mL, MeOH).

## Data Availability

The data underlying this study are available in the published article and its online Supporting Information.

## Supporting Information Available

Screening of reaction conditions of glycolurils (Tables) and NMR and MS spectra for all new compounds (PDF)
