# Supplementary material for: Synthesis of Enantiomerically Pure Bambus[6]urils Utilizing Orthogonal Protection of Glycolurils
Source: J Org Chem. 2023 Jul 28;88(16):11514–22. doi: 10.1021/acs.joc.3c00667 (PMC10442914; doi:10.1021/acs.joc.3c00667)
Supplement: Supplementary file 1 — jo3c00667_si_001.pdf [file jo3c00667_si_001.pdf]

Supporting Information

Synthesis of Enantiomerically Pure Bambus[6]urils Utilizing Orthogonal Protection of Glycolurils

Petr Slávik, Jacopo Torrisi, Pia Jurček, Jan Sokolov, Vladimír Šindelář*

Department of Chemistry, Faculty of Science, Masaryk University, 625 00 Brno, Czech Republic; RECETOX, Faculty of Science, Masaryk University, 625 00 Brno, Czech Republic

*Email: sindelar@chemi.muni.cz

Table of Contents

1. Screening of reaction conditions S2
2. ^{1}H, $^{13}C\{^{1}H\}$ and $^{19}F\{^{1}H\}$ NMR spectra S5
3. MS spectra S81
4. References S89

1. Screening of reaction conditions

Table S1. Screening of reaction conditions for synthesis of urea **3**.

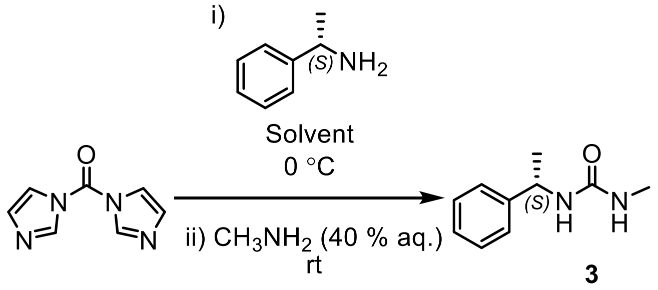

entry	solvent	reaction time (h)	yield (%)
1[a]	H_2O	i) 1	
		ii) up to 4	0[b]
2	THF	i) 1[c]	
		ii) o/n	65[b,d]
3	THF	i) 1[e]	
		ii) o/n	91[b,f]
4	THF	i) 5[g]	
		ii) o/n	92[h]

[a] Reaction conditions by Padiya *et al.*[1] [b] Yield based on ^{1}H NMR analysis. [c] Side product 2*N*,4*N'*-(*S*)-1-phenylethylurea 35 %. [d] (*S*)-1-Phenylethylamine was added in one portion. [e] (*S*)-1-Phenylethylamine was added dropwise. [f] Side product 2*N*,4*N'*-(*S*)-1-phenylethylurea 9 %. [g] (*S*)-1-Phenylethylamine was added dropwise via syringe pump. [h] Isolated yield. No side product observed.

Table S2. Screening of reaction conditions for protection of amides of glycoluril **1a**.

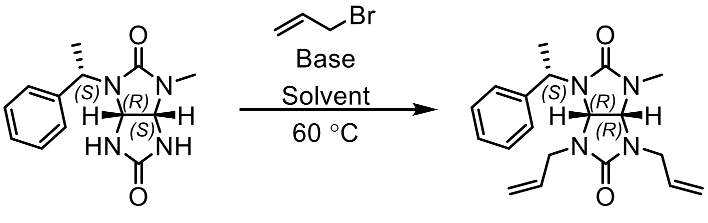

entry	base	solvent	reaction time (h)	yield (%)[a]
1	NaOH	DMF	16	58 and 68[b]
2	Cs_2CO_3	CH_3CN	8	90
3	Cs_2CO_3	DMF	4	96

[a] Isolated yield. [b] Decomposition of starting material and product.

Table S3. Screening of reaction conditions for deprotection of (*S*)-phenylethyl of glycoluril **4**.

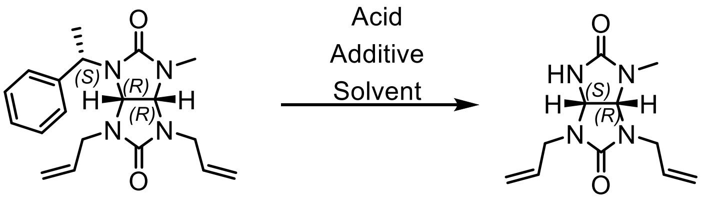

entry	acid	additive	solvent	temp. (°C)	reaction time (h)	yield (%)[a]
1[b]	MsOH	-	toluene	110	8	0
2	Formic acid	-	-	rt	days	0[c]
3	Formic acid	-	-	60	days	0[c]
4	TFA	-	-	60	3	70
5	TFA	1,4-dimethoxybenzene	-	60	3	81

[a] Isolated yields. [b] Reaction conditions by Paik and Lee.[2] [c] Decomposition of starting material and product.

Table S4. Screening of reaction conditions for deprotection of allyl groups of glycoluril **8c**.

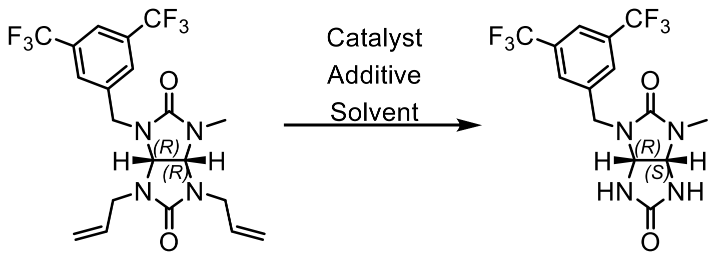

8c **9c**

entry	catalyst	additive	solvent	temp. (°C)	reaction time (h)	yield (%)[a]
1[b]	$RhCl_3$	-	*n*-propanol	97	16	46
2[c]	Ru cat.[d]	KIO_4	H_2O/CH_3CN	80	72	0
3[e]	$[Pd(CF_3COO)_2]$	dppp[f]	H_2O/CH_3CN	60	144	85
4[g]	$[Pd(CF_3COO)_2]$	dppp[f]	H_2O/CH_3CN	120	0.5	89

[a] Isolated yields. [b] Reaction conditions by Zacuto and Xu.[3] [c] Reaction conditions by Cadierno *et al.*[4] [d] Dichloro-[(2,6,10-dodecatriene)-1,12-diyl]ruthenium(IV). [e] Reaction conditions by Ohmura *et al.*[5] [f] 1,3-Bis(diphenyl-phosphino)propane. [g] Reaction was done in a microwave reactor: 150 W max., 300 PSI max., medium stirring.

2. ^{1}H, $^{13}C\{^{1}H\}$ and $^{19}F\{^{1}H\}$ NMR spectra

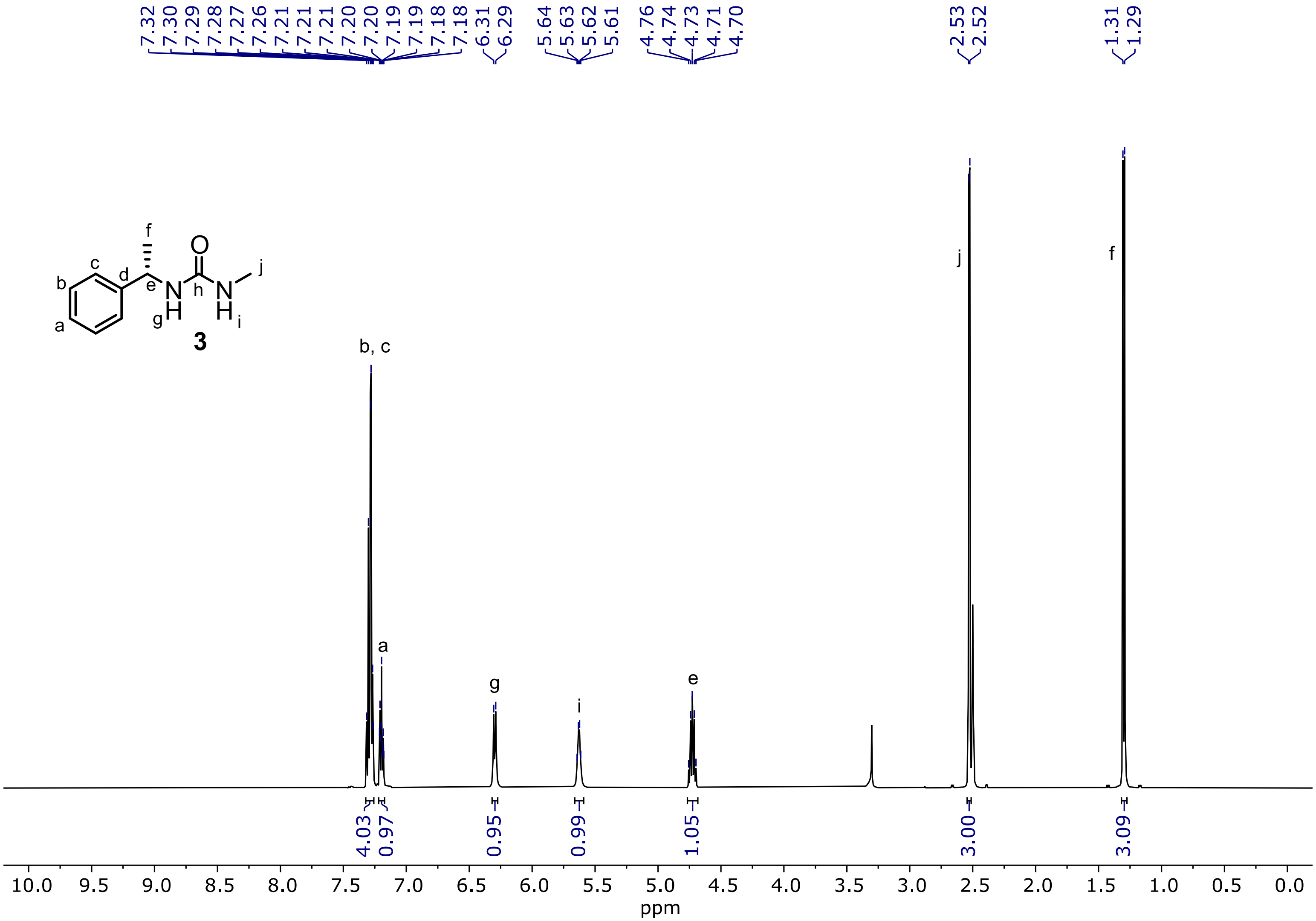

Figure S1. ^{1}H NMR spectrum (500 MHz, DMSO-d_6, 303 K) of urea **3.**

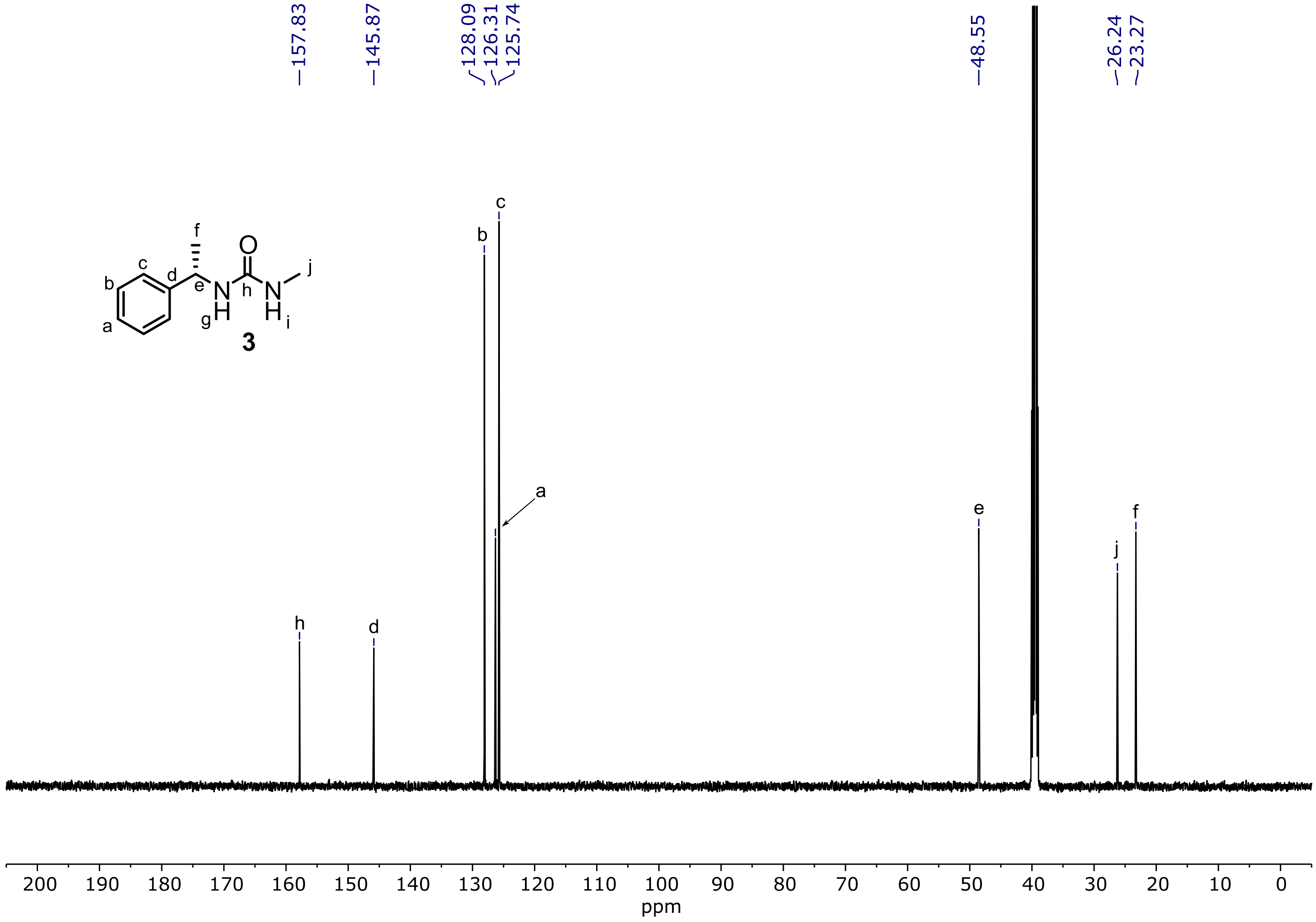

Figure S2. ^{1}H NMR spectrum (500 MHz, DMSO-d_6, 303 K) of urea **3.**

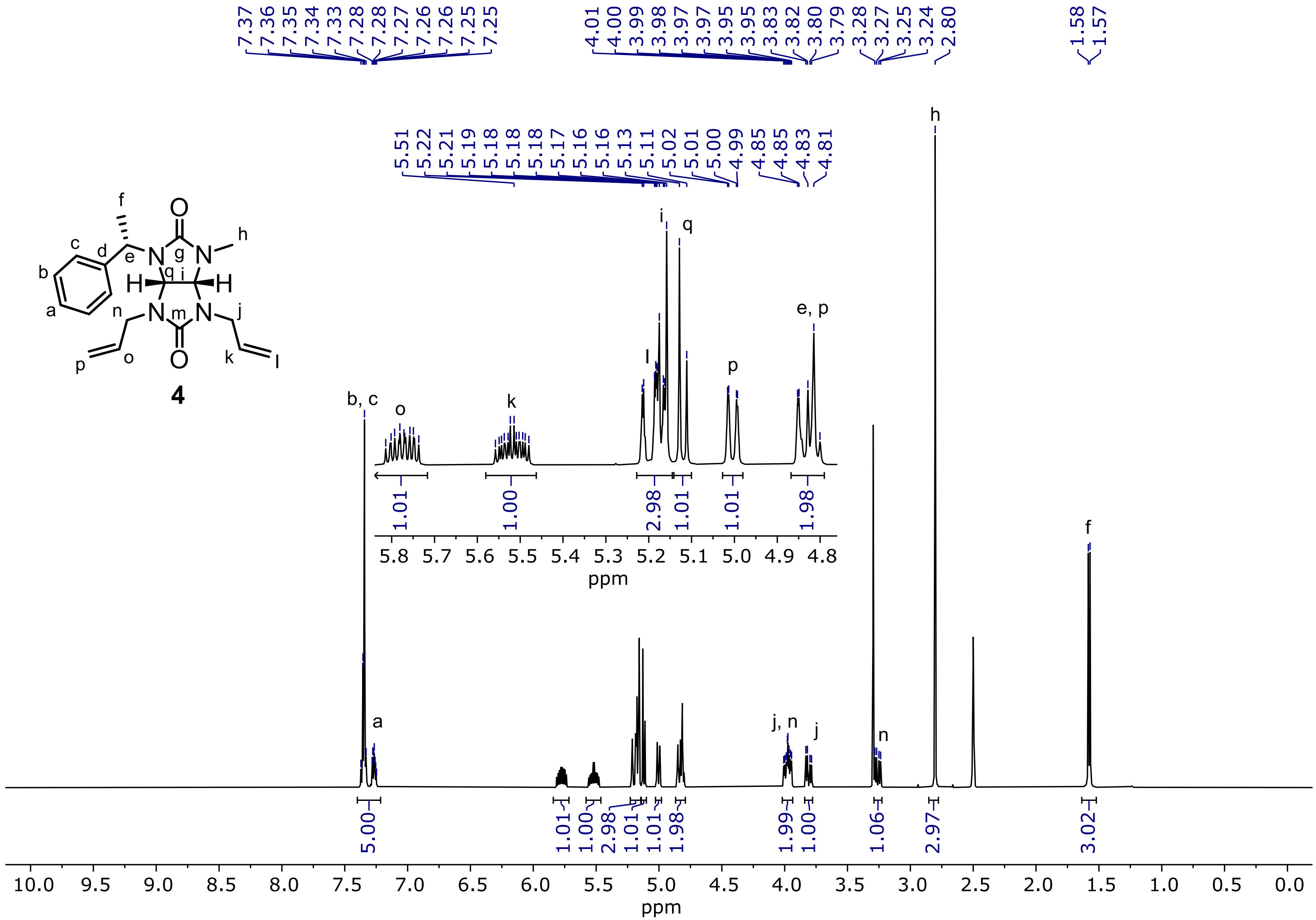

Figure S3. ^{1}H NMR spectrum (500 MHz, DMSO-d_6, 303 K) of glycoluril **4**.

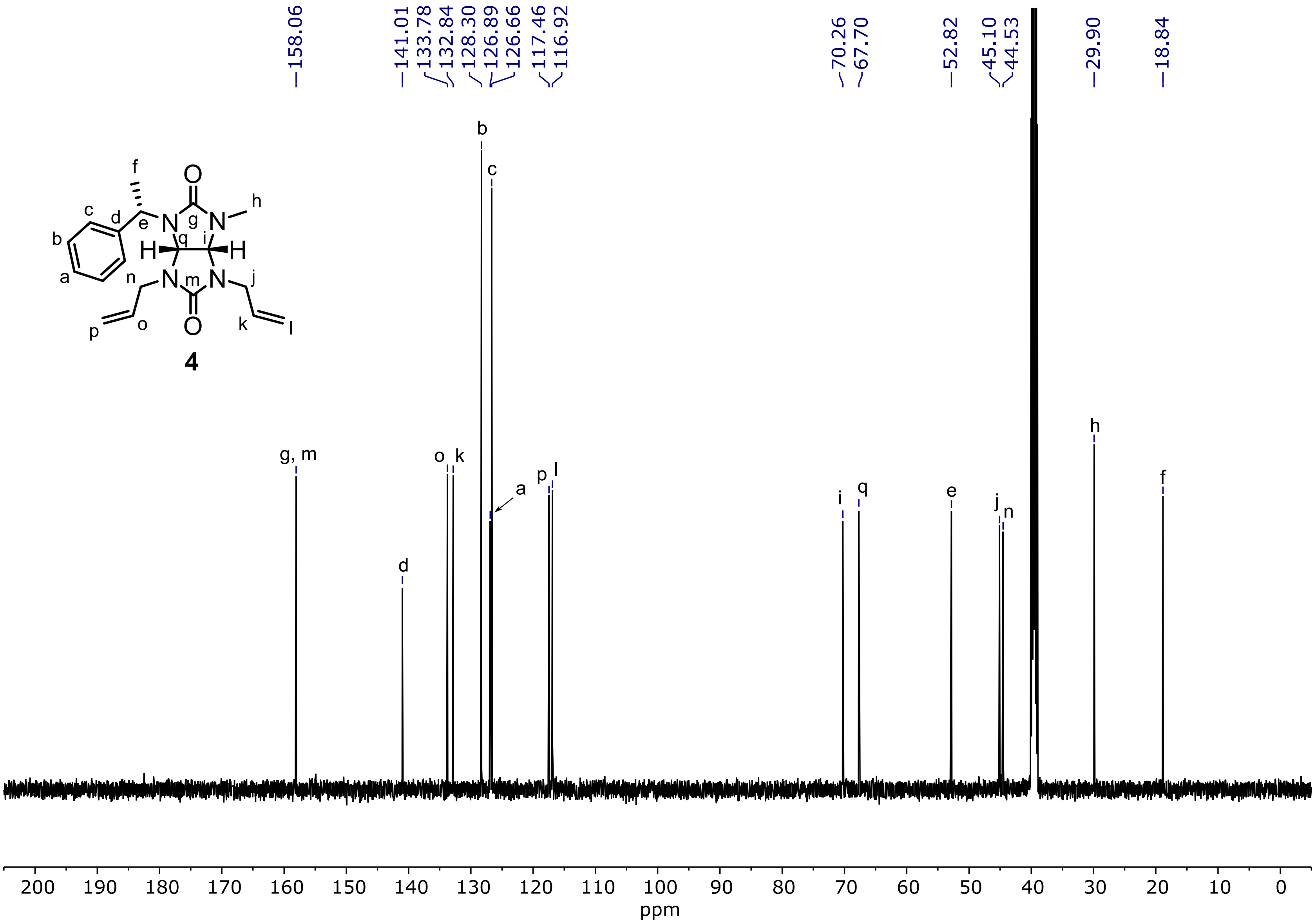

Figure S4. $^{13}C\{^{1}H\}$ NMR spectrum (126 MHz, DMSO-d_6, 303 K) of glycoluril **4**.

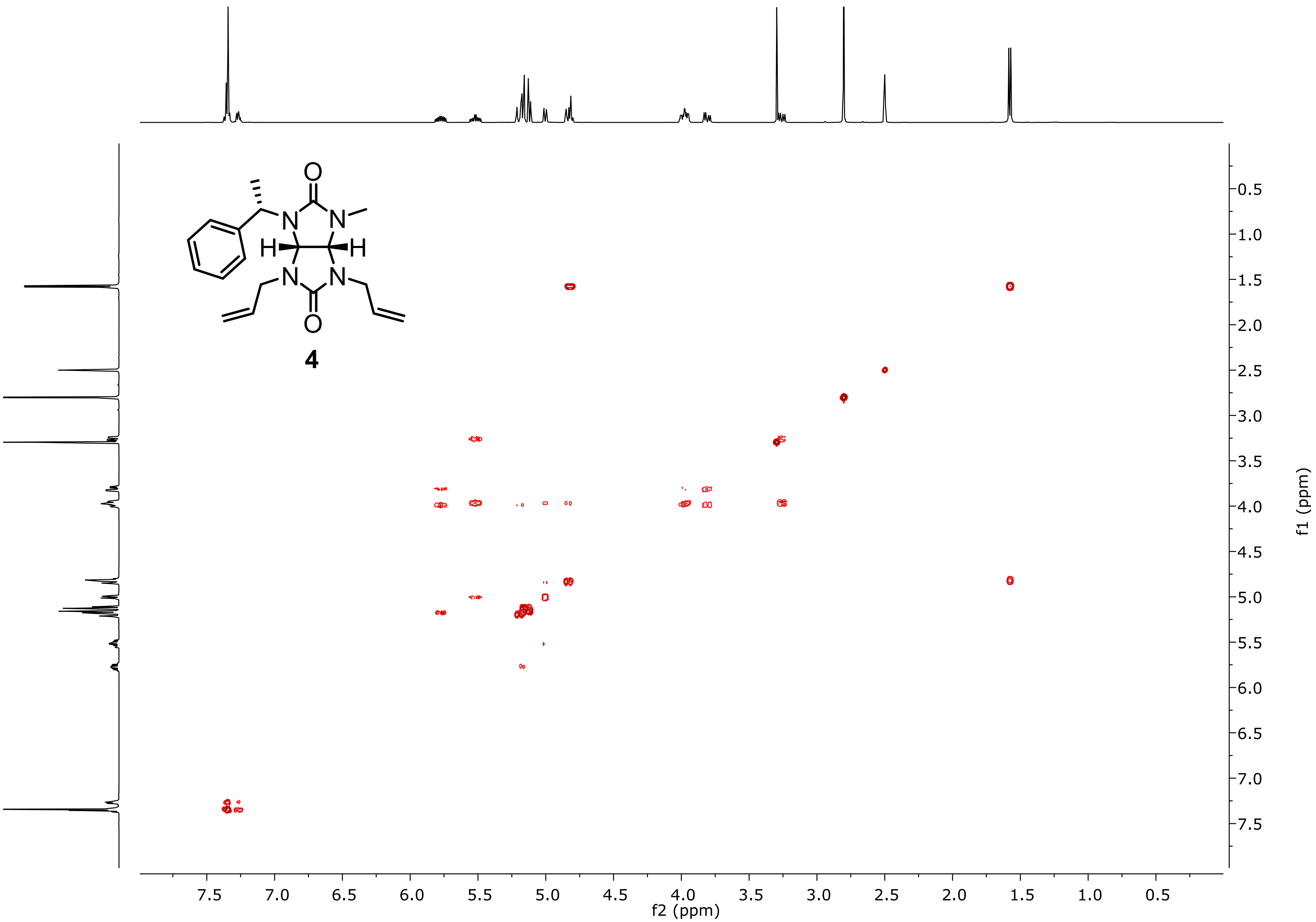

Figure S5. COSY NMR spectrum (500 MHz, DMSO-d_6, 303 K) of glycoluril **4**.

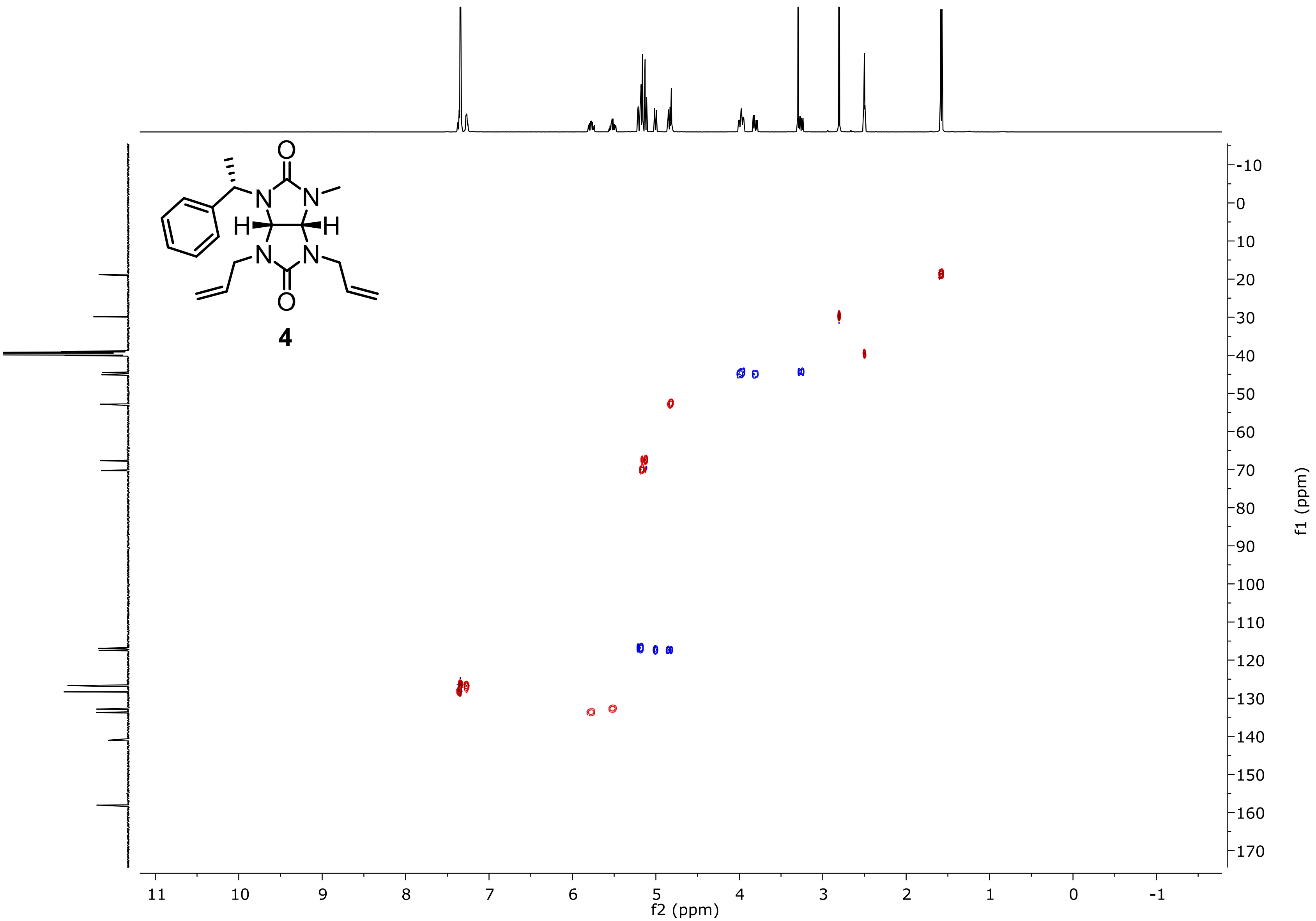

Figure S6. HSQC NMR spectrum (500 MHz, DMSO-d_6, 303 K) of glycoluril **4**.

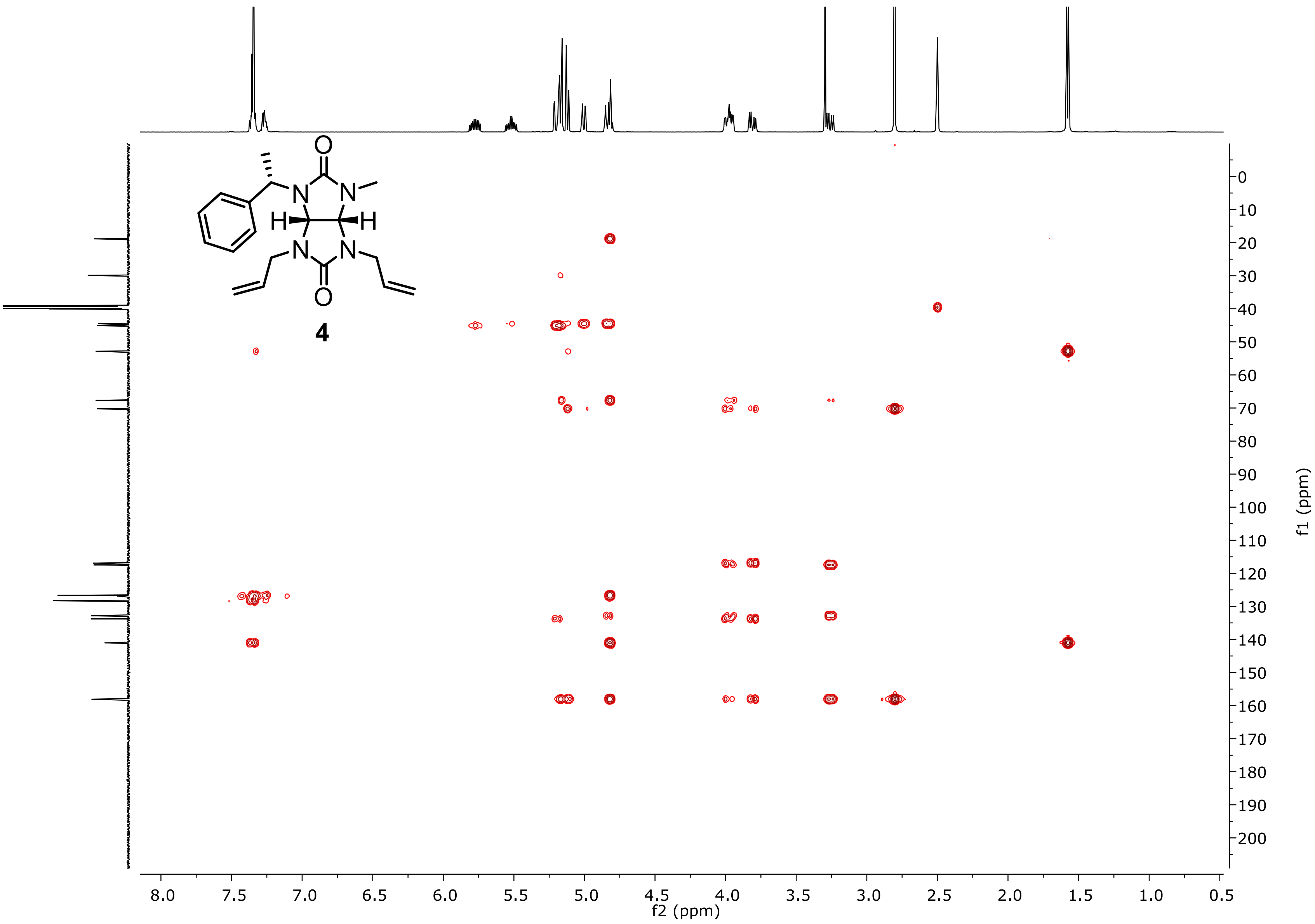

Figure S7. HMBC NMR spectrum (500 MHz, DMSO-d_6, 303 K) of glycoluril **4**.

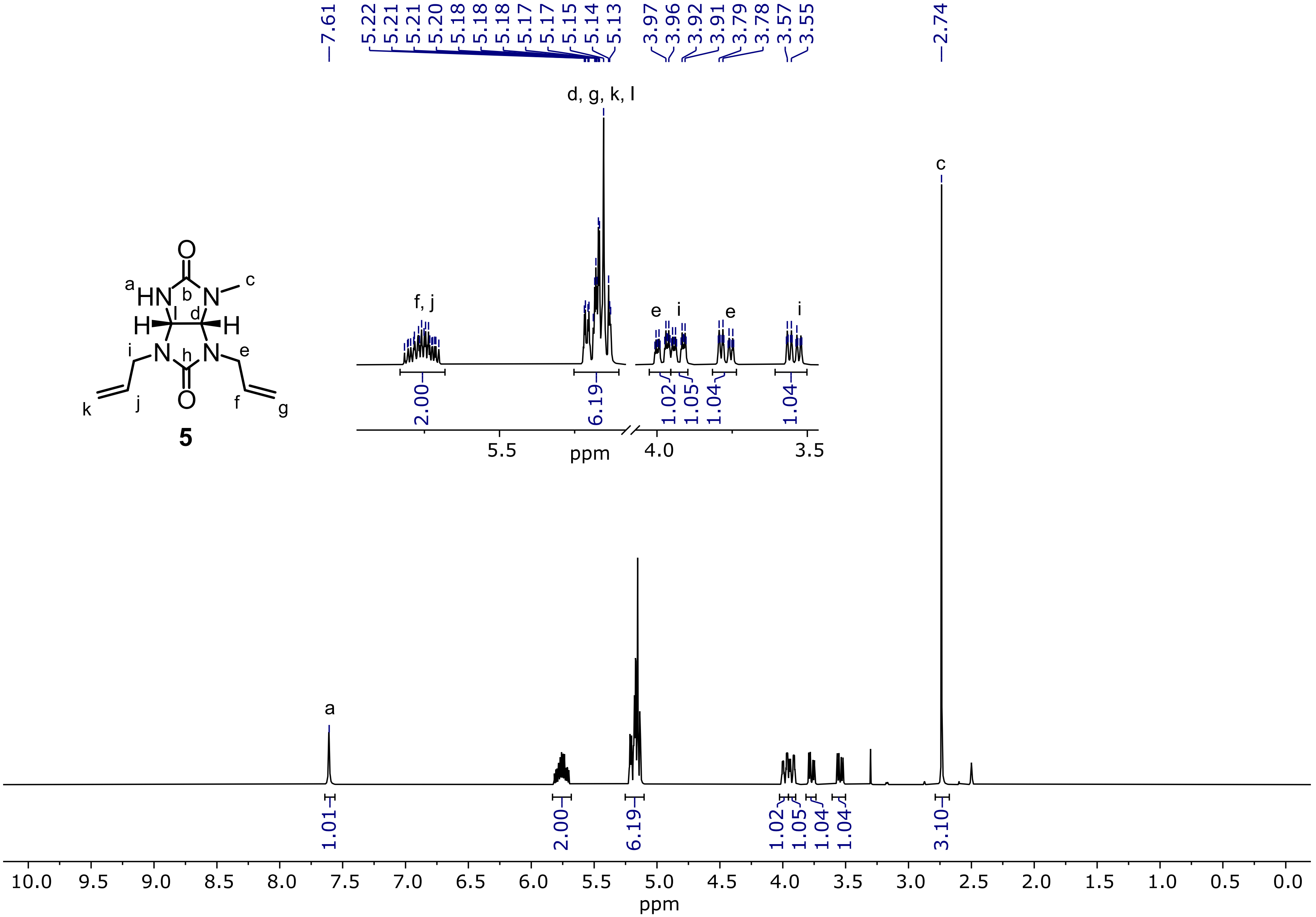

Figure S8. ^{1}H NMR spectrum (500 MHz, DMSO-d_6, 303 K) of glycoluril **5**.

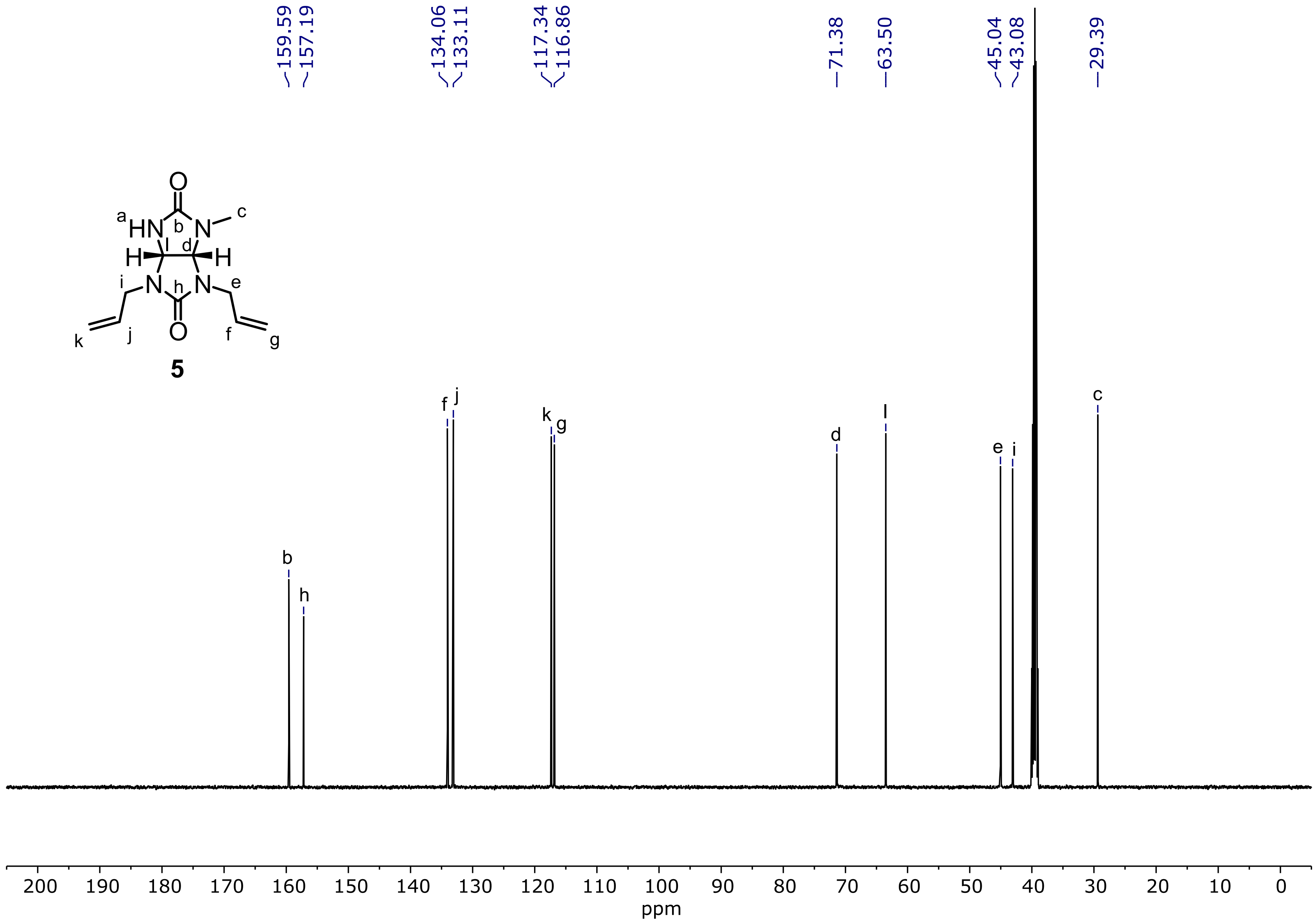

Figure S9. $^{13}C\{^{1}H\}$ NMR spectrum (126 MHz, DMSO-d_6, 303 K) of glycoluril **5**.

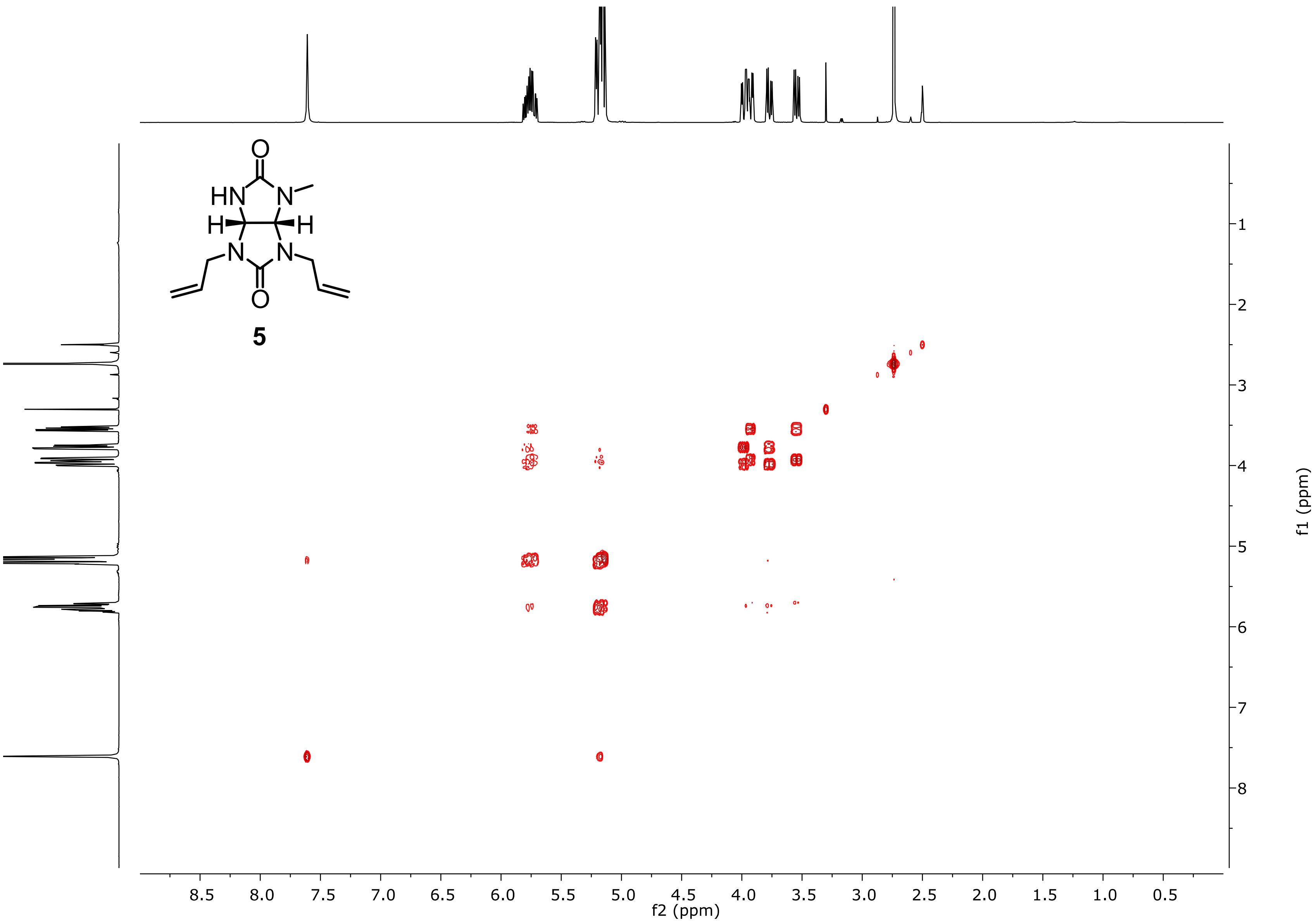

Figure S10. COSY NMR spectrum (500 MHz, DMSO-d_6, 303 K) of glycoluril **5**.

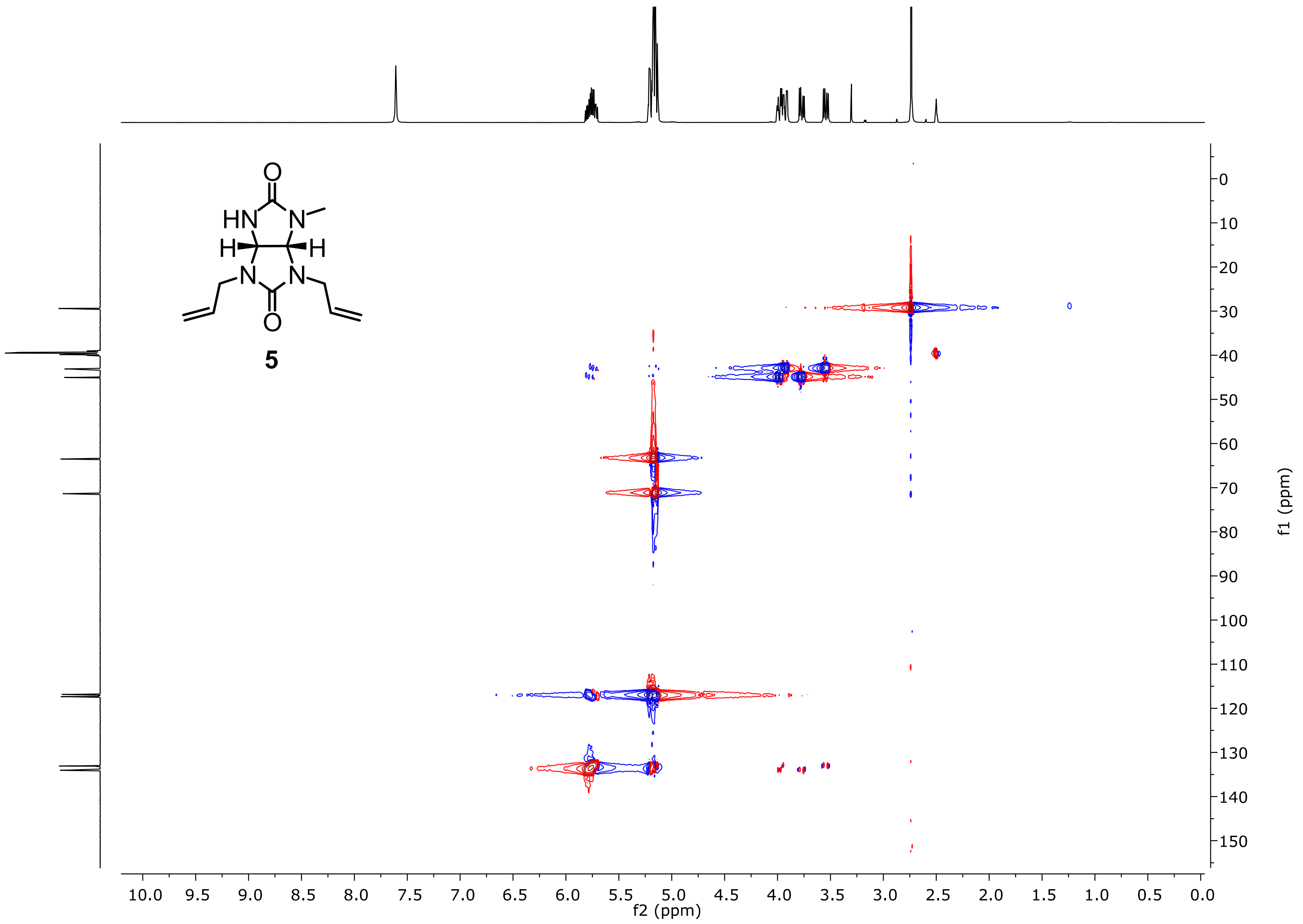

Figure S11. HSQC NMR spectrum (500 MHz, DMSO-d_6, 303 K) of glycoluril **5**.

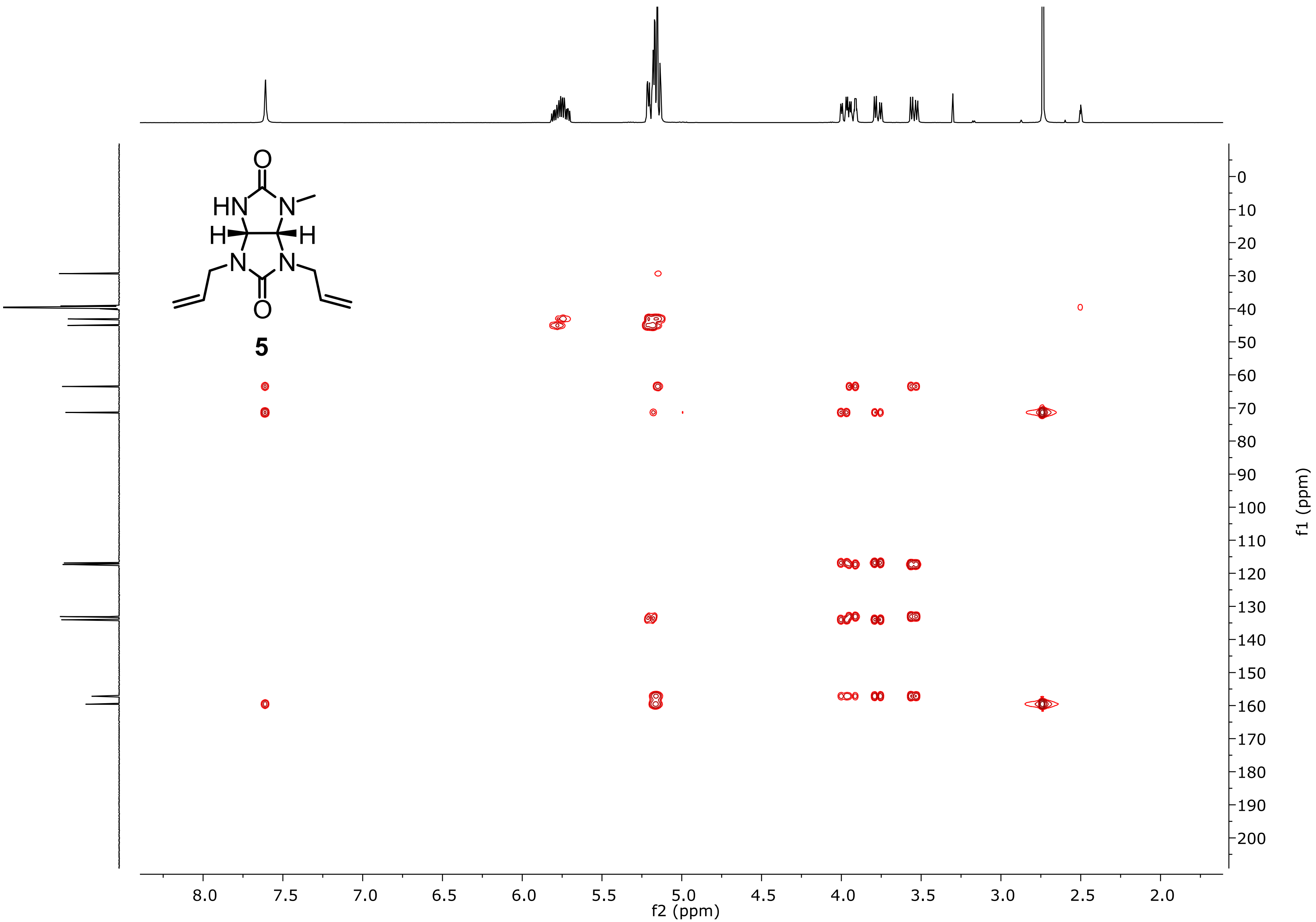

Figure S12. HMBC NMR spectrum (500 MHz, DMSO-d_6, 303 K) of glycoluril **5**.

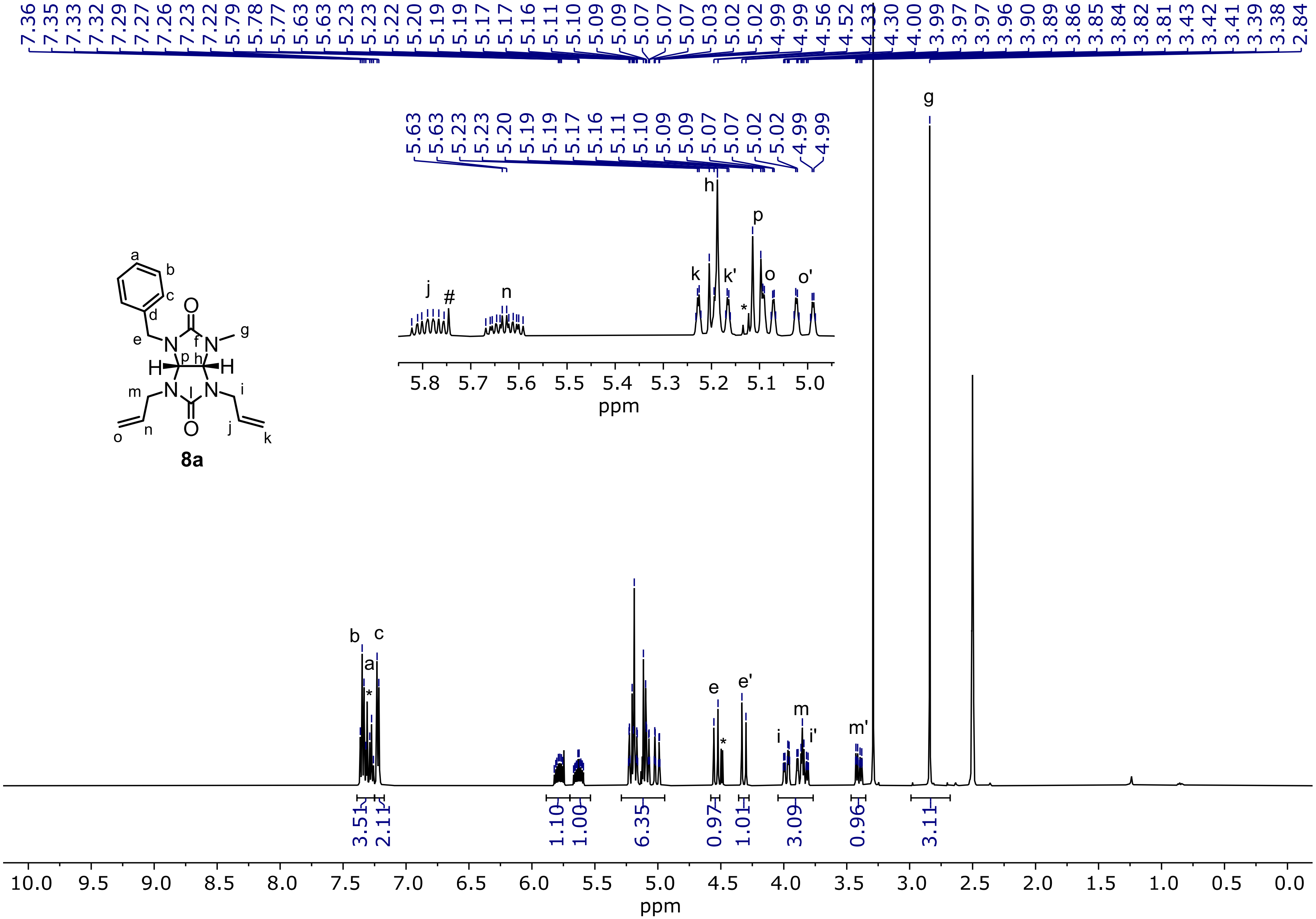

Figure S13. ^{1}H NMR spectrum (500 MHz, DMSO-d_6, 303 K) of glycoluril **8a** (*benzyl alcohol; #DCM).

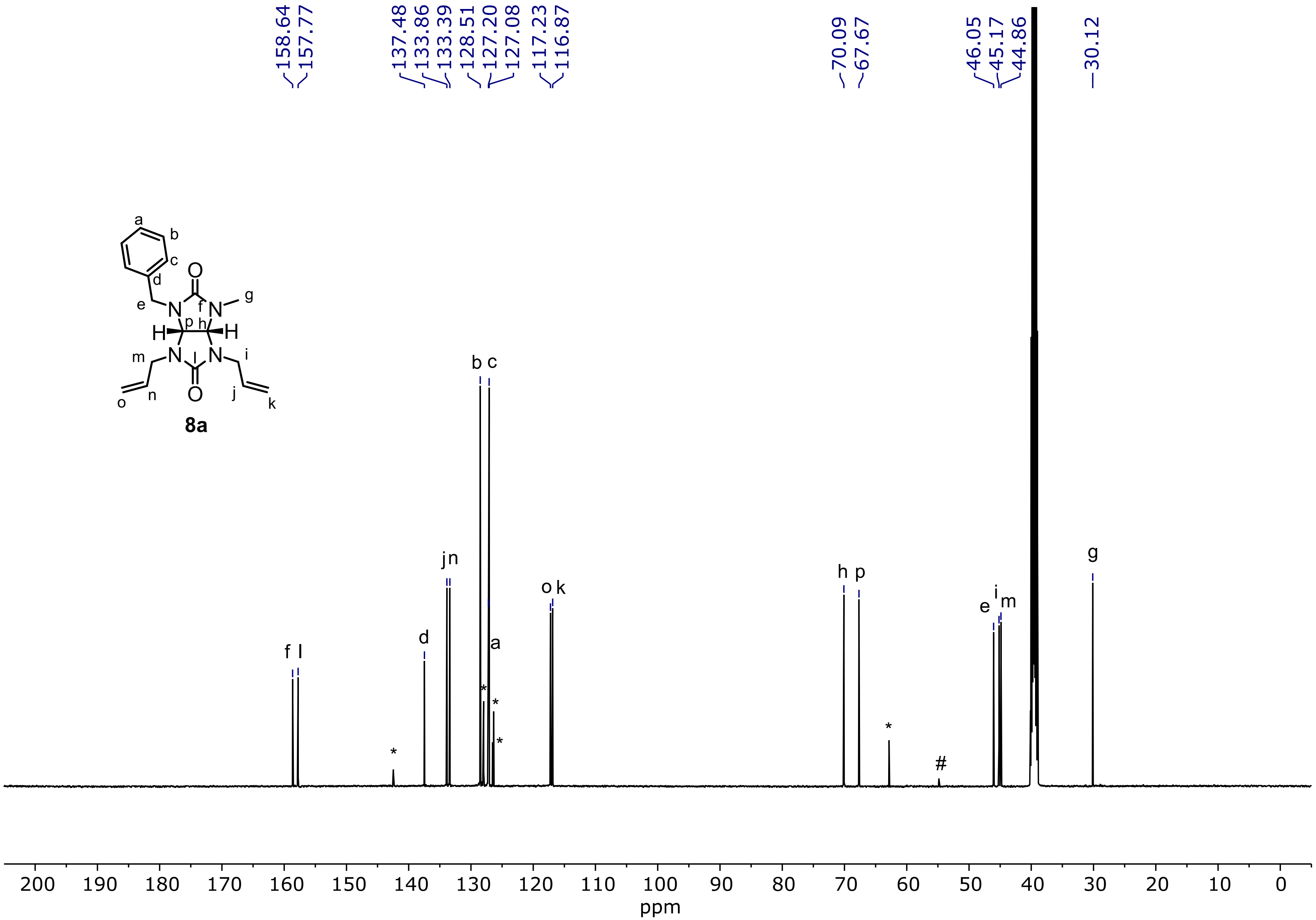

Figure S14. $^{13}C\{^{1}H\}$ NMR spectrum (126 MHz, DMSO-d_6, 303 K) of glycoluril **8a** (*benzyl alcohol; #DCM).

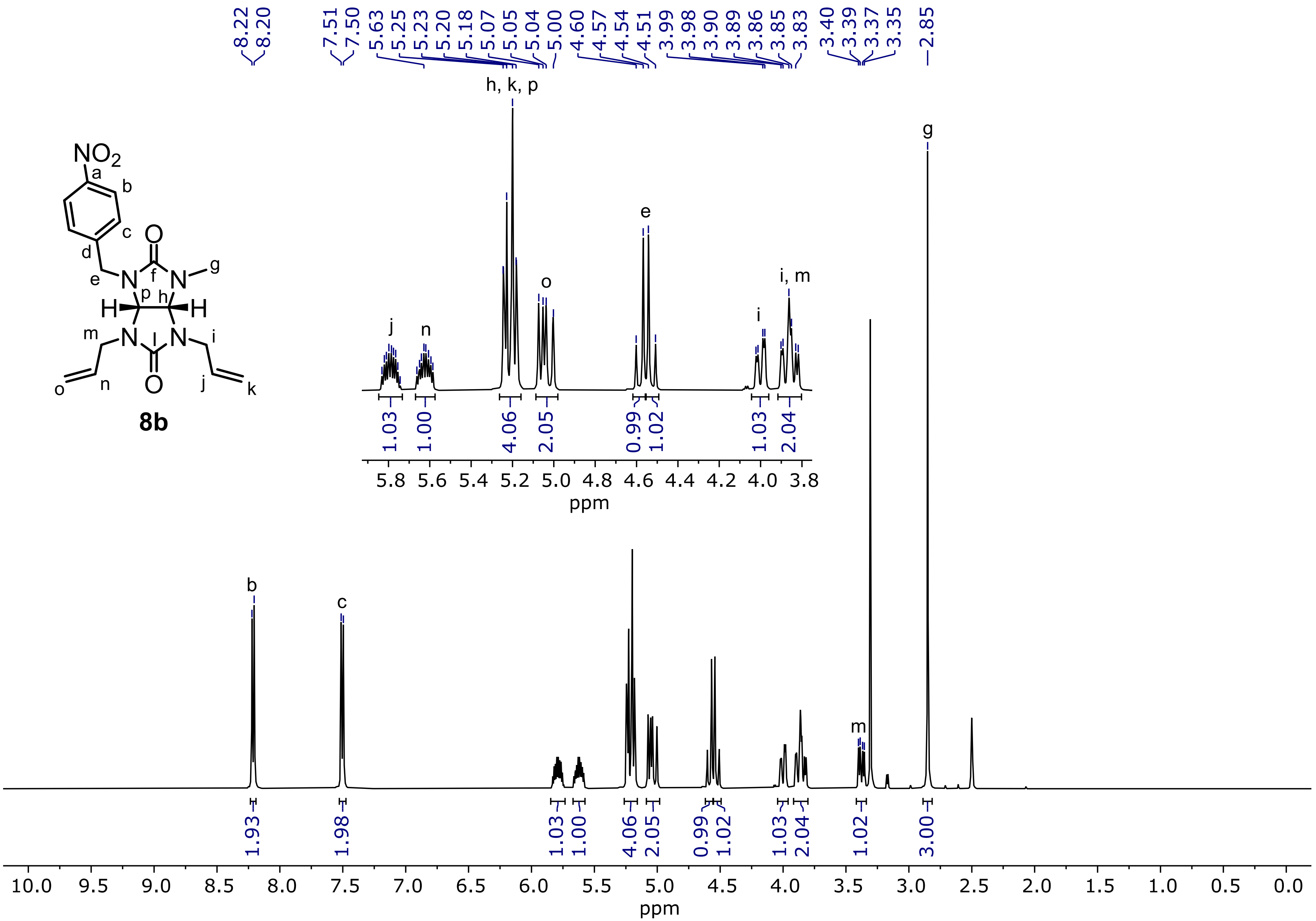

Figure S15. ^{1}H NMR spectrum (500 MHz, DMSO-d_6, 303 K) of glycoluril **8b**.

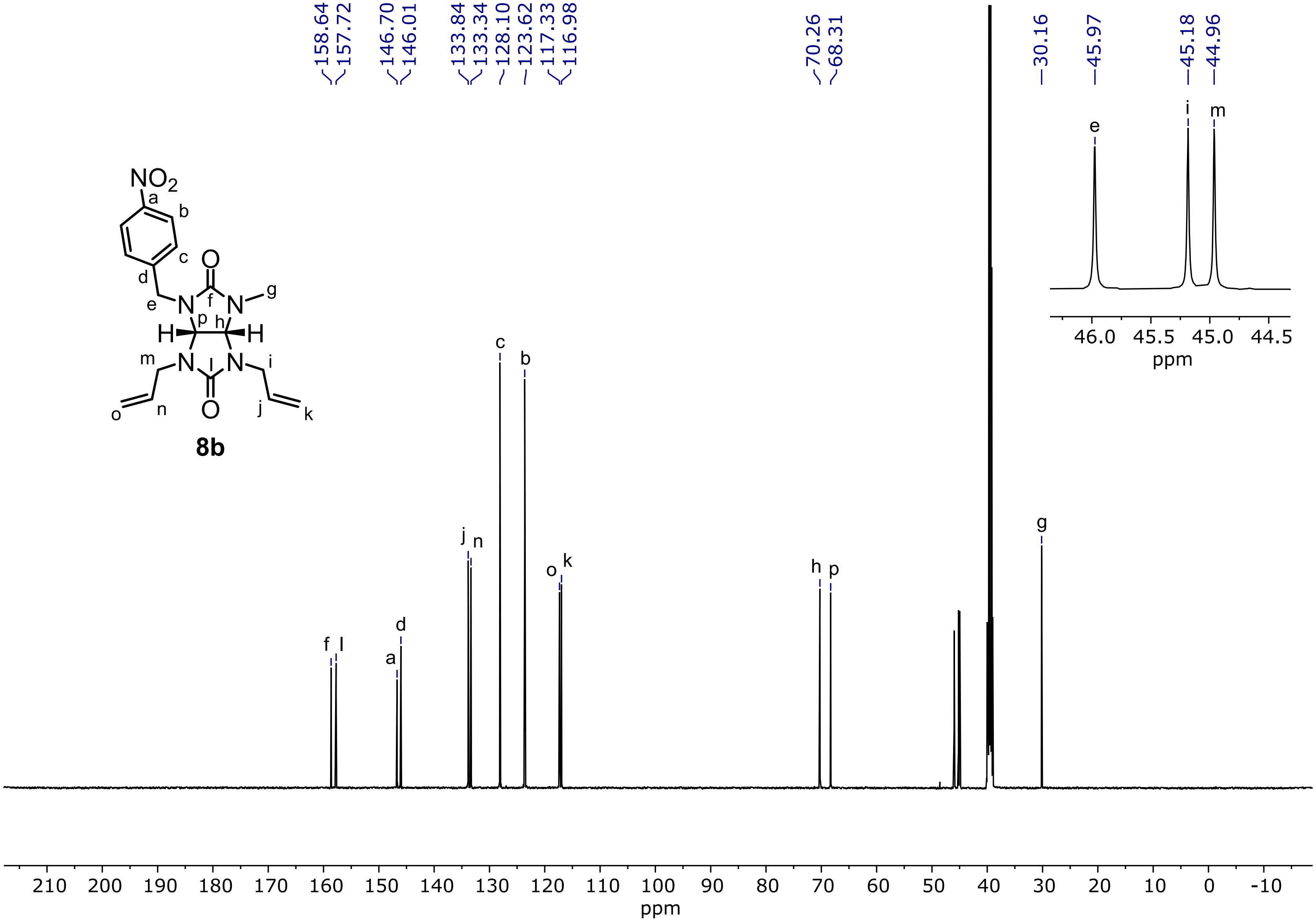

Figure S16. $^{13}C\{^{1}H\}$ NMR spectrum (126 MHz, DMSO-d_6, 303 K) of glycoluril **8b**.

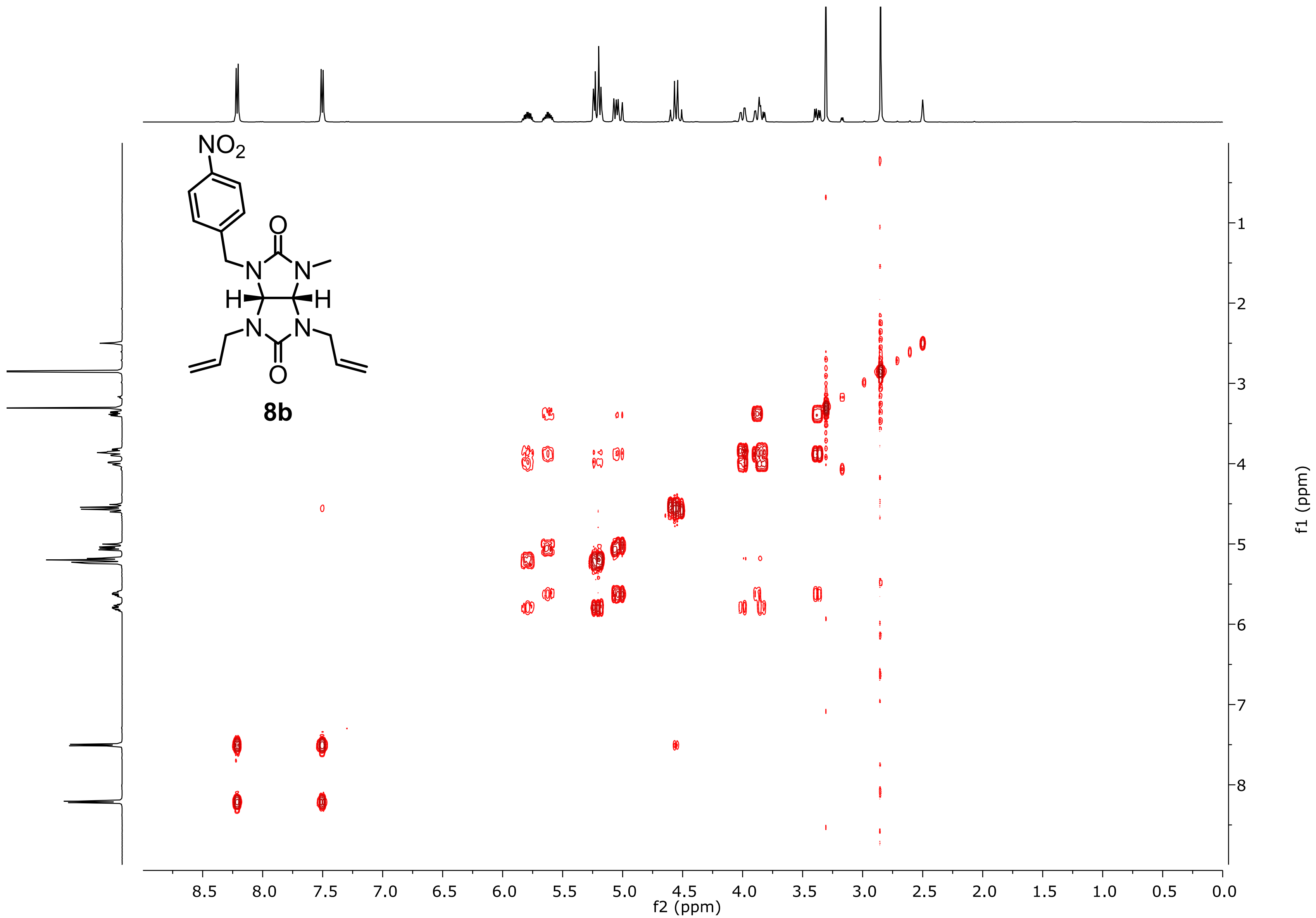

Figure S17. COSY NMR spectrum (500 MHz, DMSO-d_6, 303 K) of glycoluril **8b**.

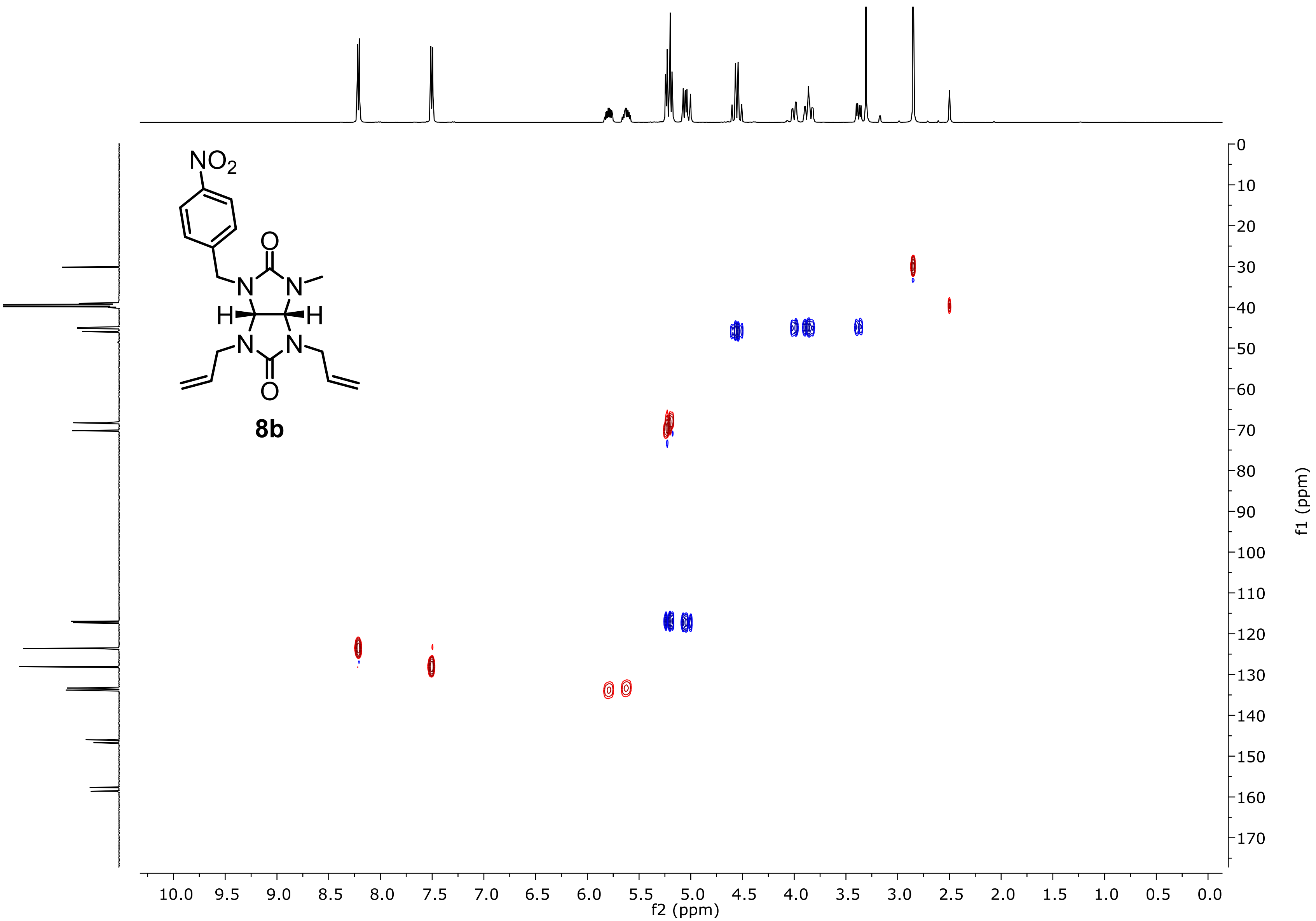

Figure S18. HSQC NMR spectrum (500 MHz, DMSO-d_6, 303 K) of glycoluril **8b**.

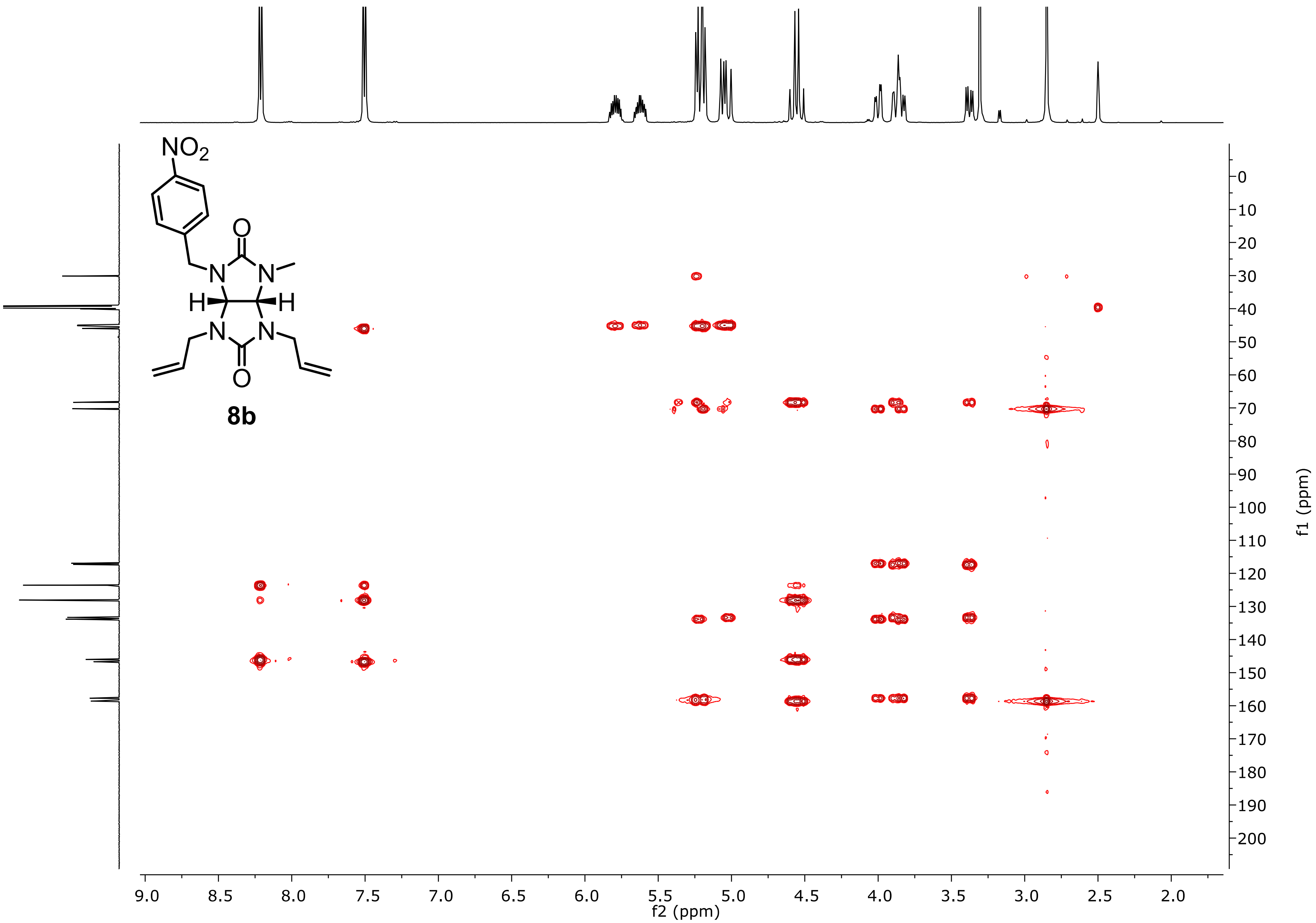

Figure S19. HMBC NMR spectrum (500 MHz, DMSO-d_6, 303 K) of glycoluril **8b**.

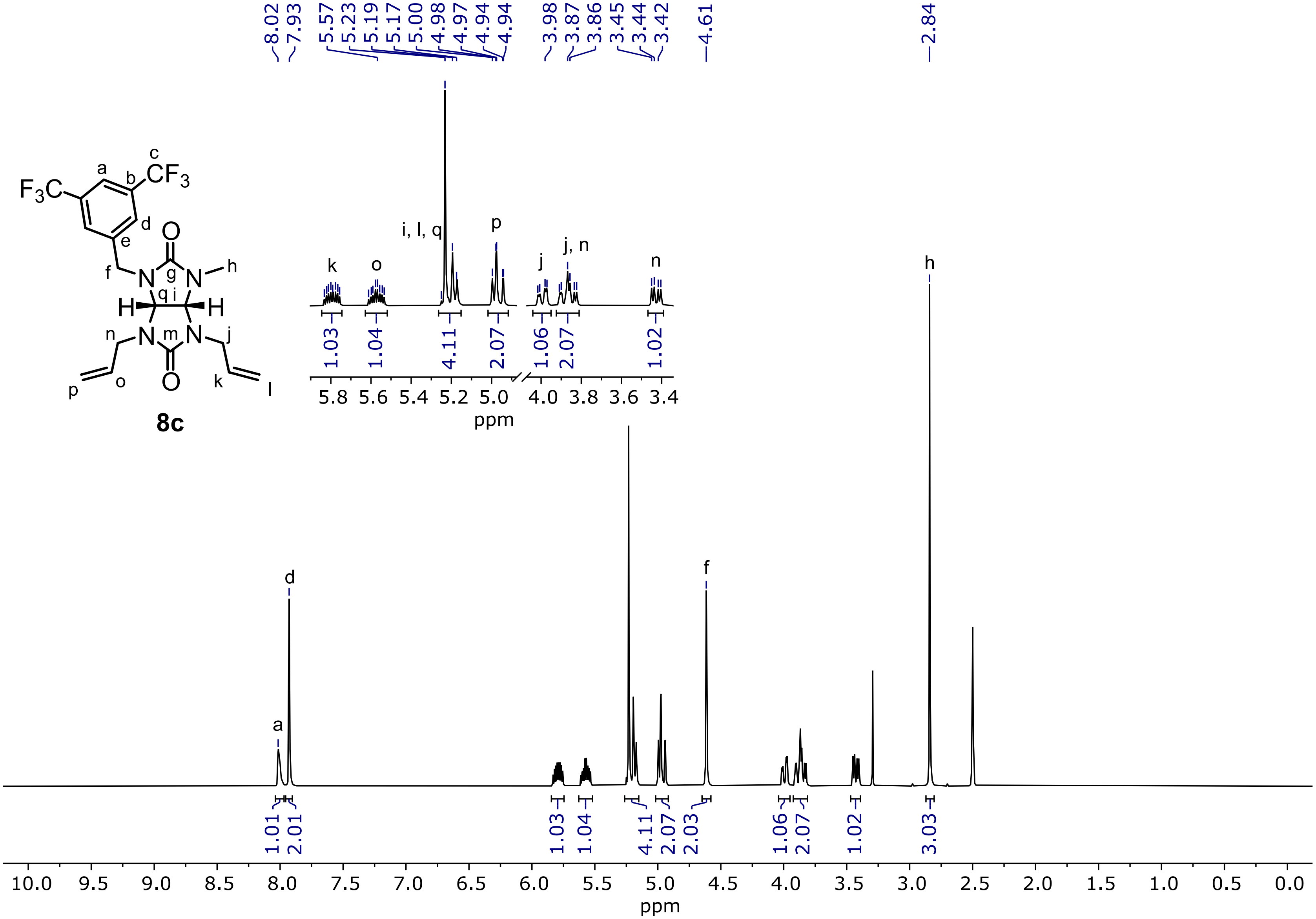

Figure S20. ^{1}H NMR spectrum (500 MHz, DMSO-d_6, 303 K) of glycoluril **8c**.

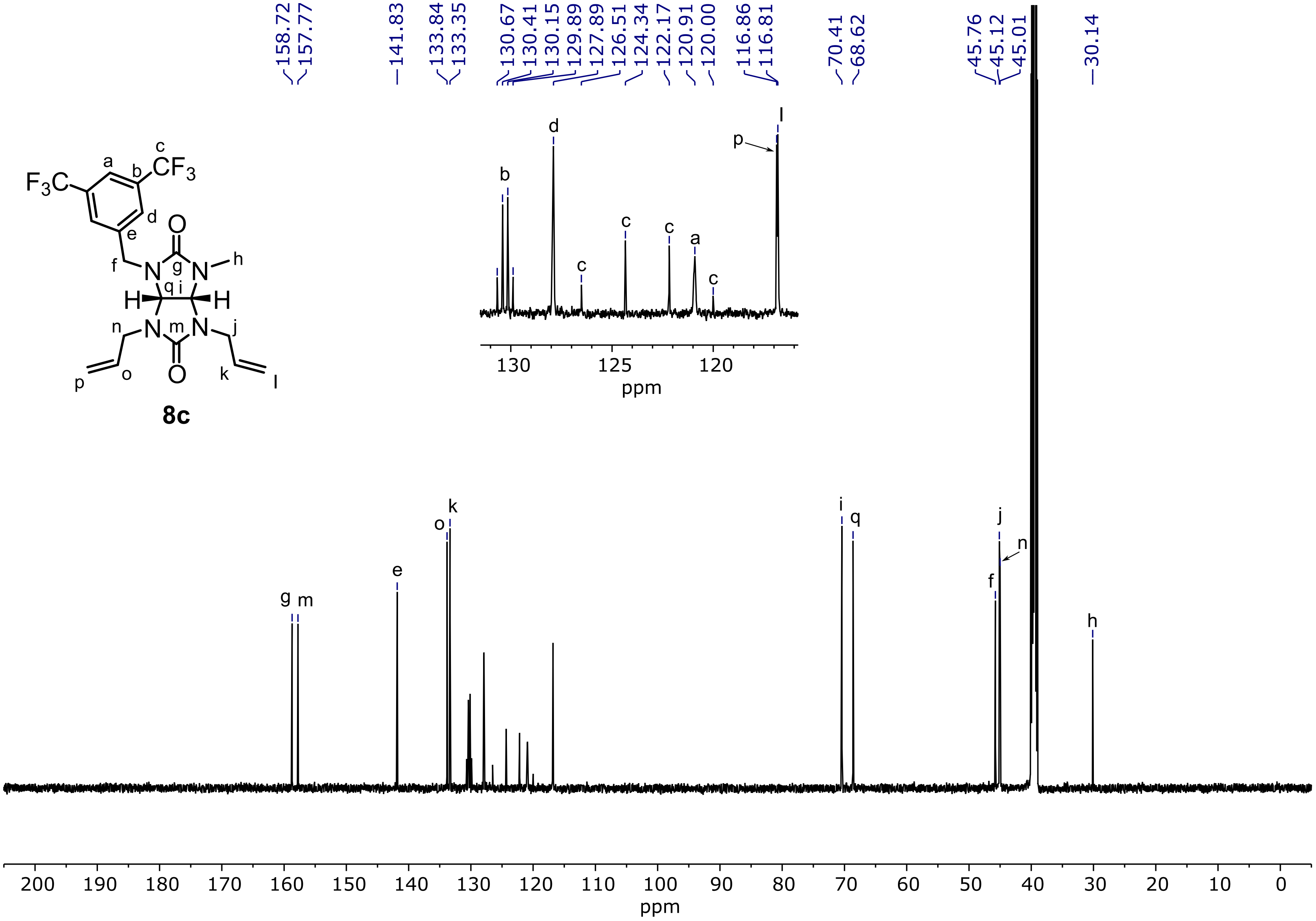

Figure S21. $^{13}C\{^{1}H\}$ NMR spectrum (126 MHz, DMSO-d_6, 303 K) of glycoluril **8c**.

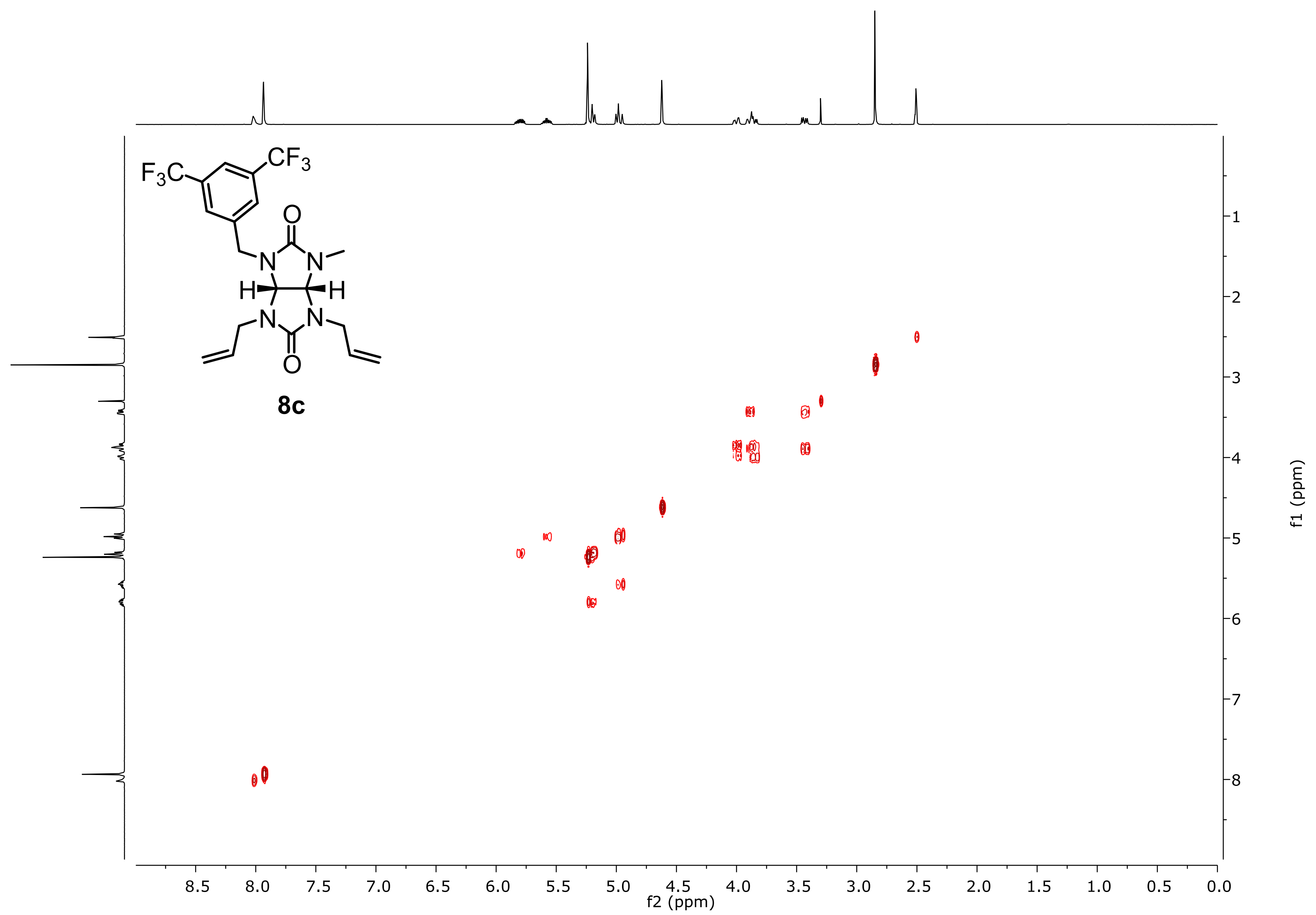

Figure S22. COSY NMR spectrum (500 MHz, DMSO-d_6, 303 K) of glycoluril **8c**.

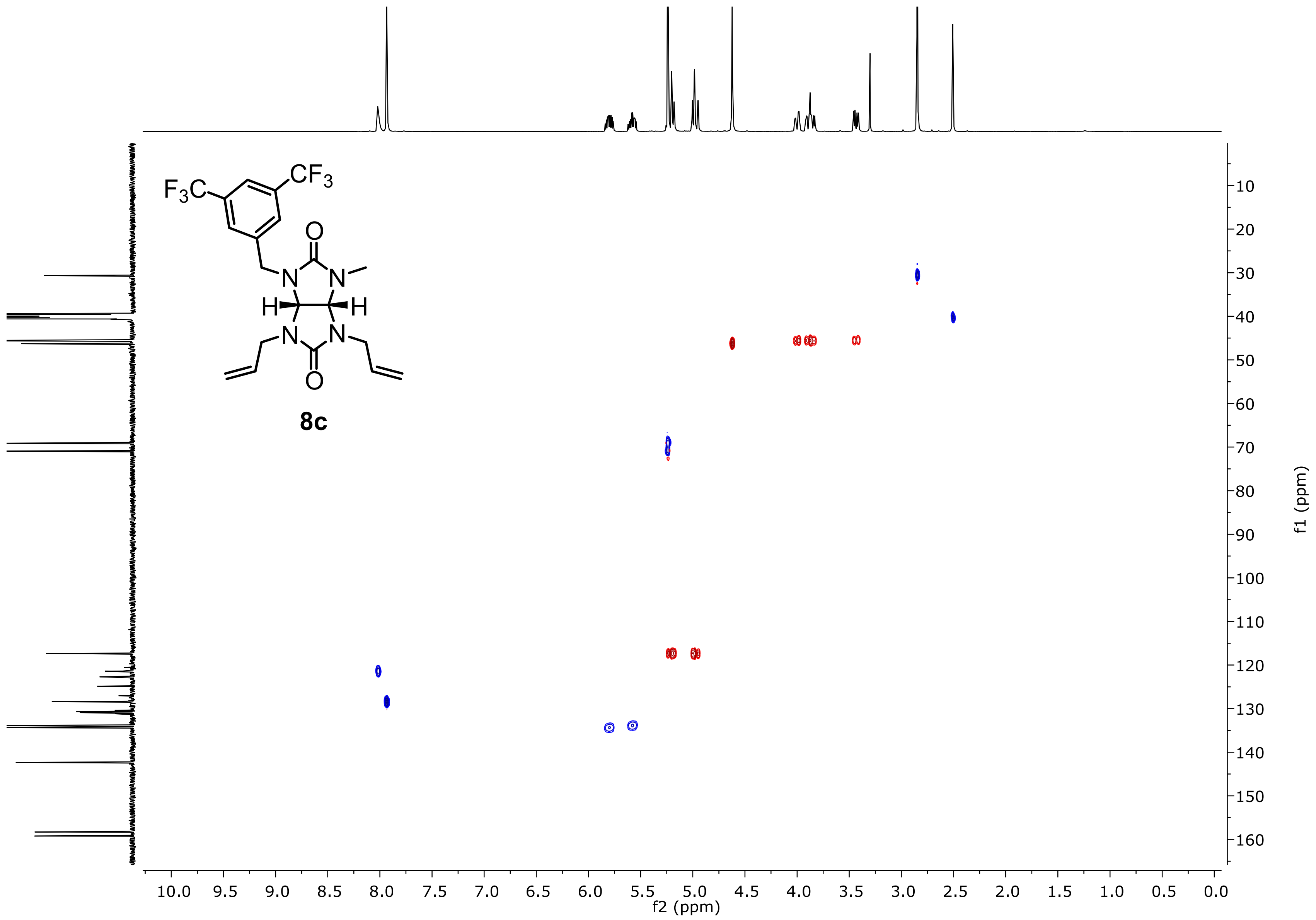

Figure S23. HSQC NMR spectrum (500 MHz, DMSO-d_6, 303 K) of glycoluril **8c**.

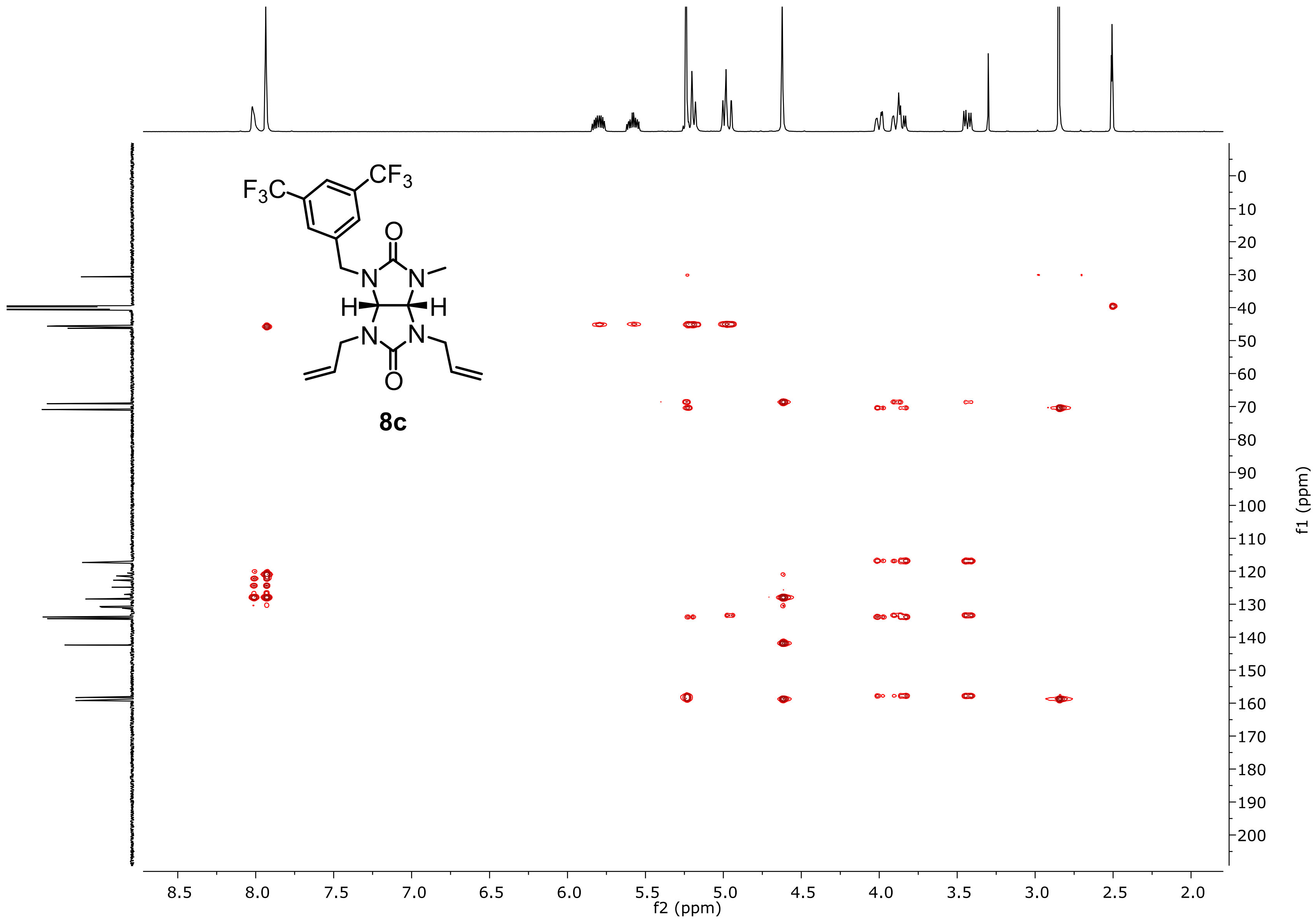

Figure S24. HMBC NMR spectrum (500 MHz, DMSO-d_6, 303 K) of glycoluril **8c**.

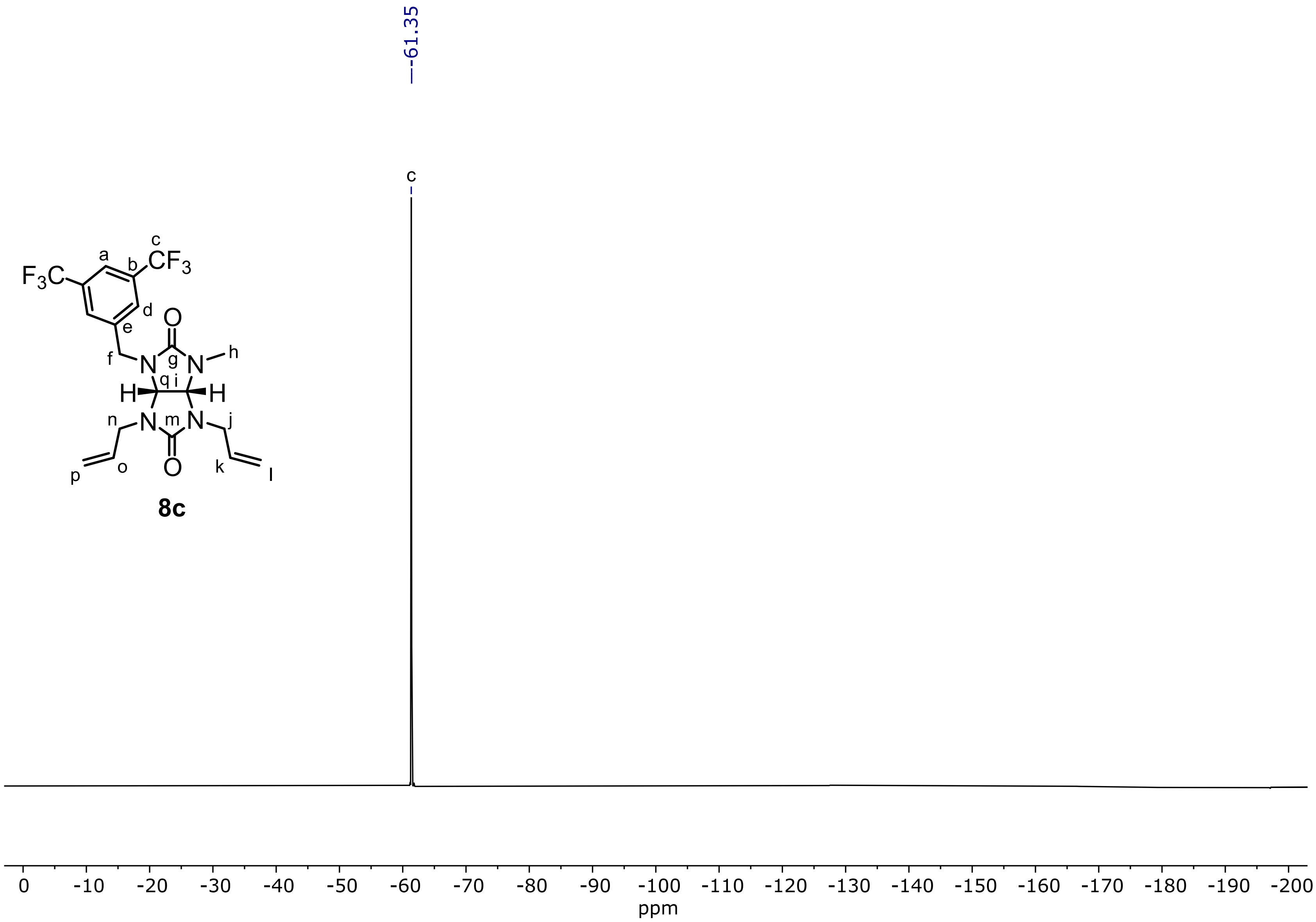

Figure S25. $^{19}F\{^{1}H\}$ NMR spectrum (471 MHz, DMSO-d_6, 303 K) of glycoluril **8c**.

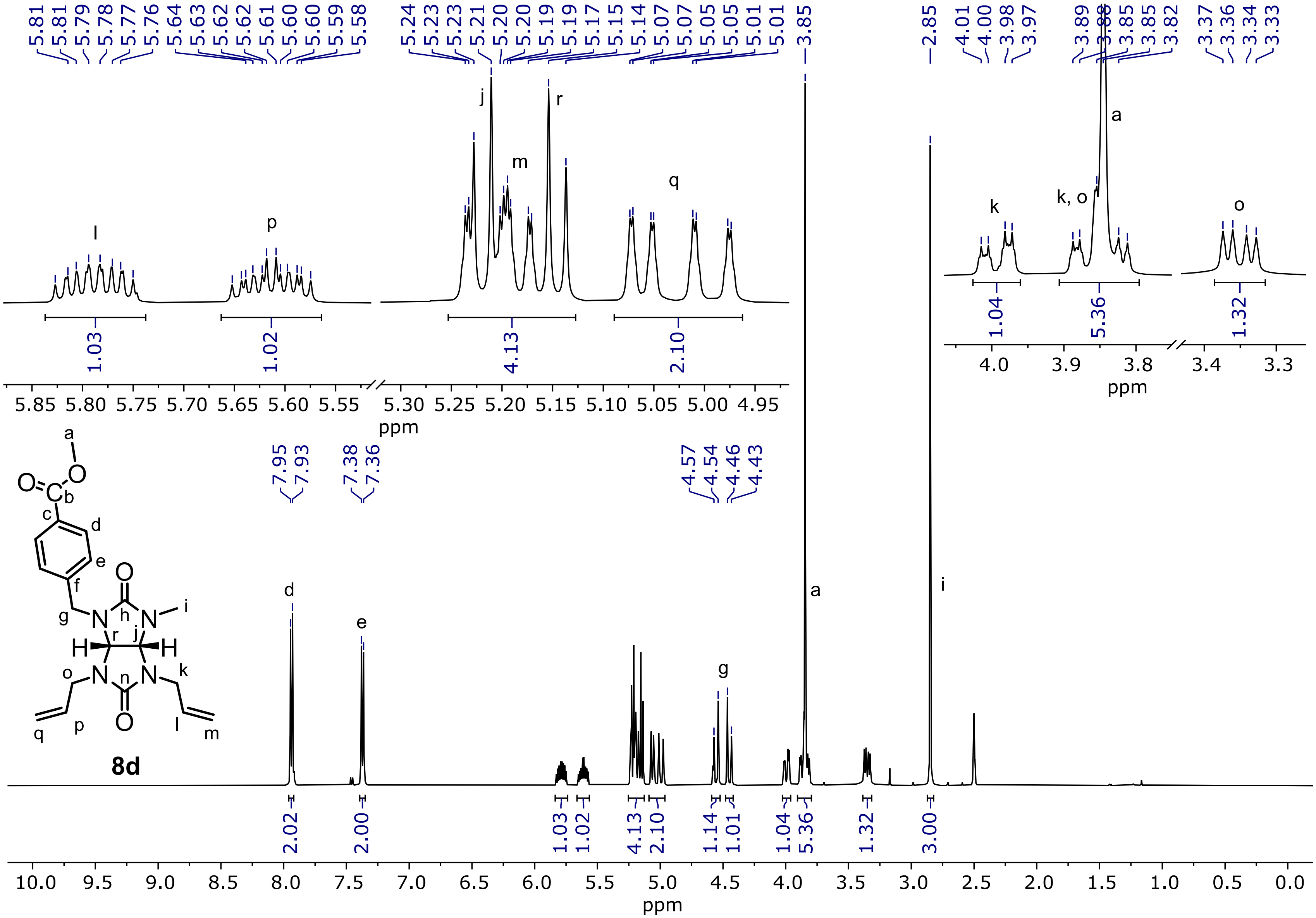

Figure S62. ^{1}H NMR spectrum (500 MHz, DMSO-d_6, 303 K) of glycoluril **8d**.

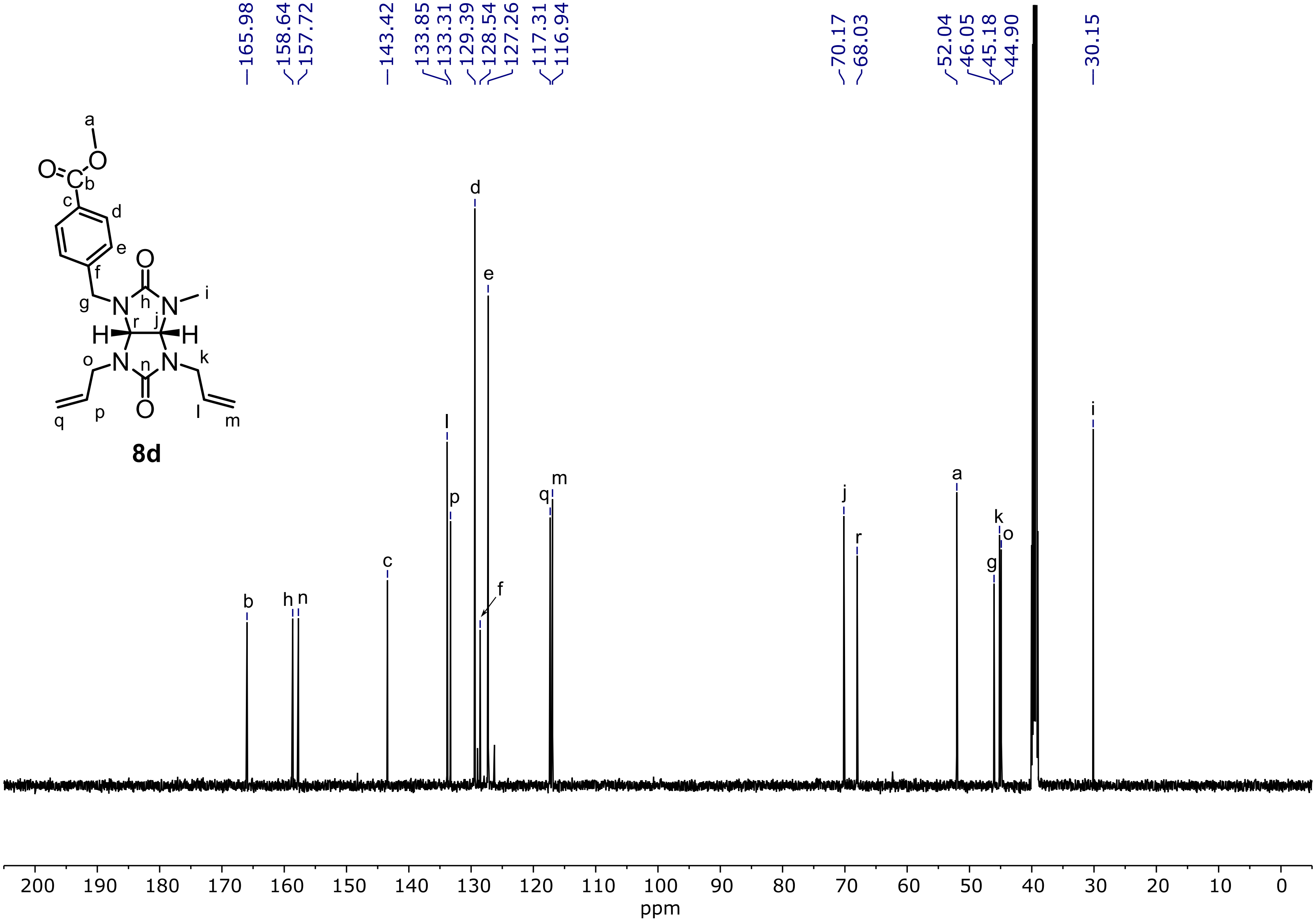

Figure S27. $^{13}C\{^{1}H\}$ NMR spectrum (126 MHz, DMSO-d_6, 303 K) of glycoluril **8d**.

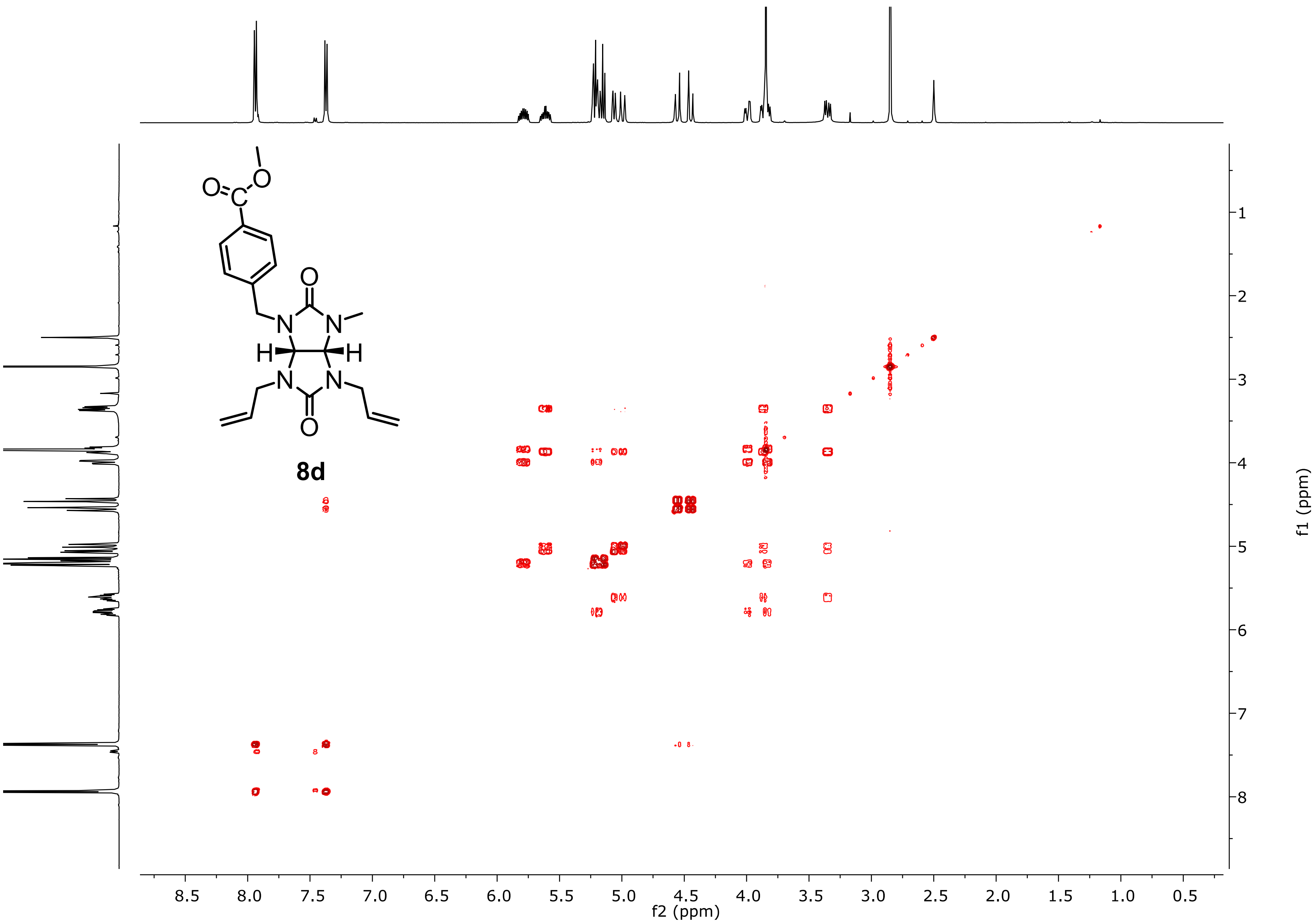

Figure S28. COSY NMR spectrum (500 MHz, DMSO-d_6, 303 K) of glycoluril **8d**.

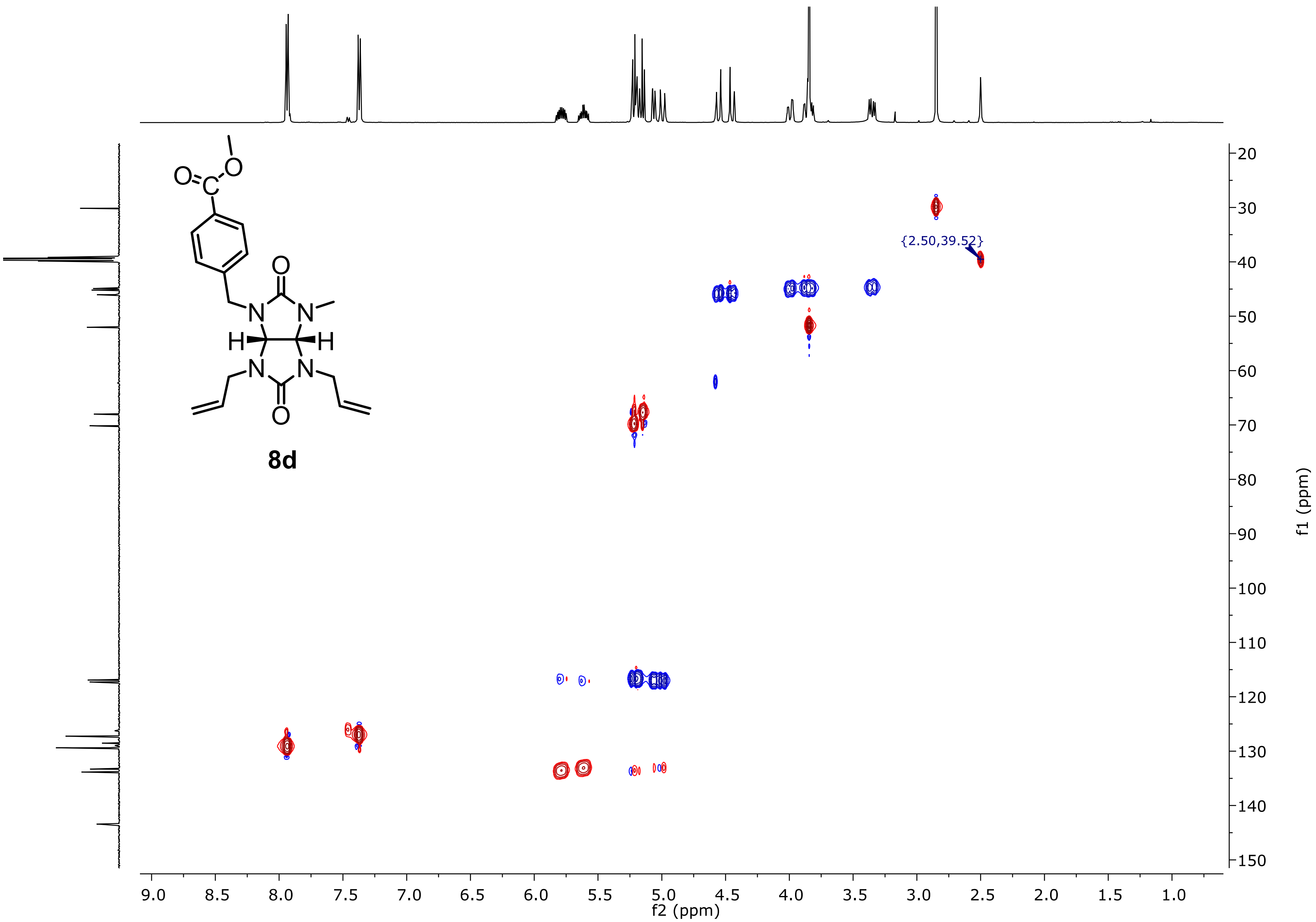

Figure S29. HSQC NMR spectrum (500 MHz, DMSO-d_6, 303 K) of glycoluril **8d**.

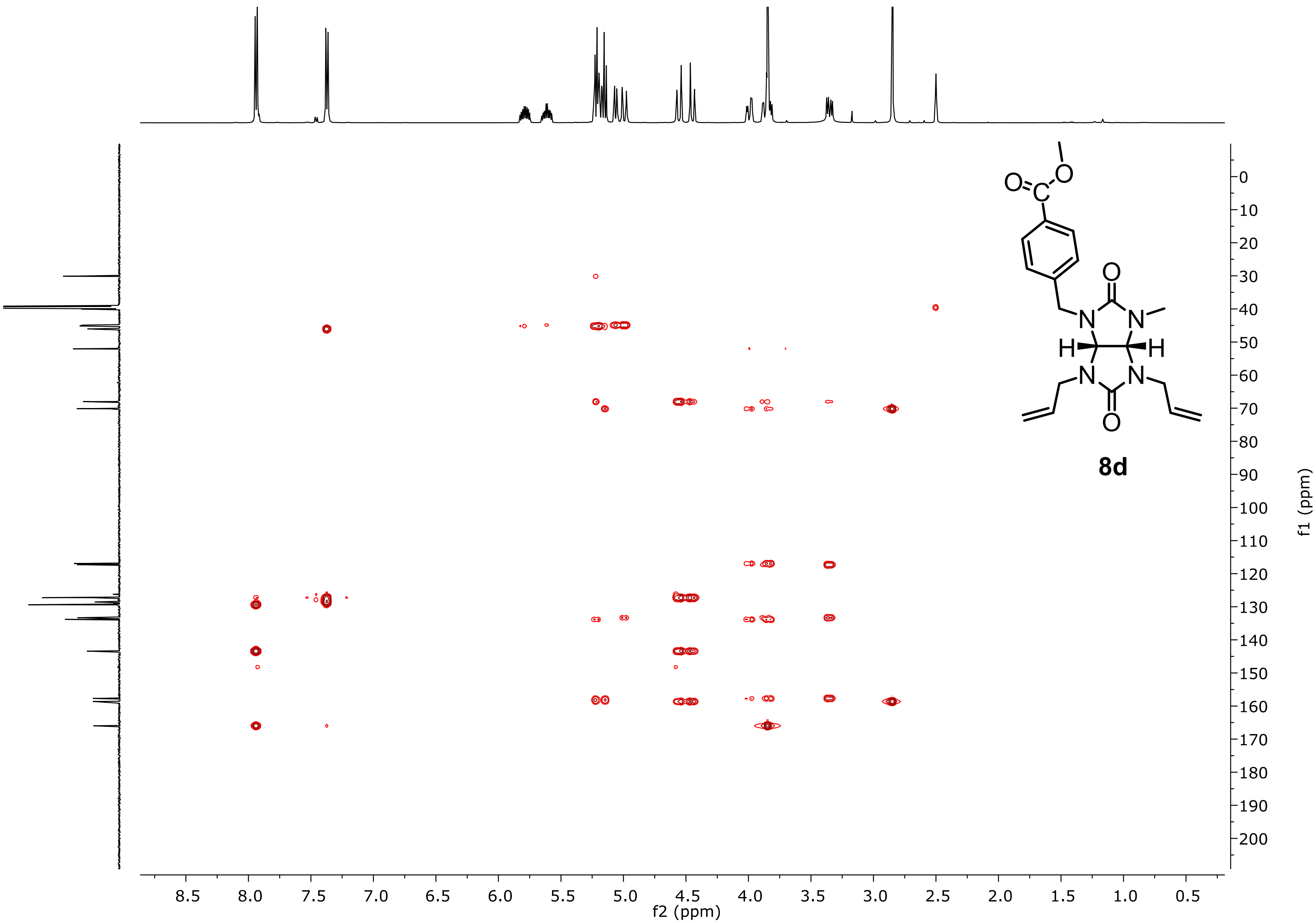

Figure S30. HMBC NMR spectrum (500 MHz, DMSO-d_6, 303 K) of glycoluril **8d**.

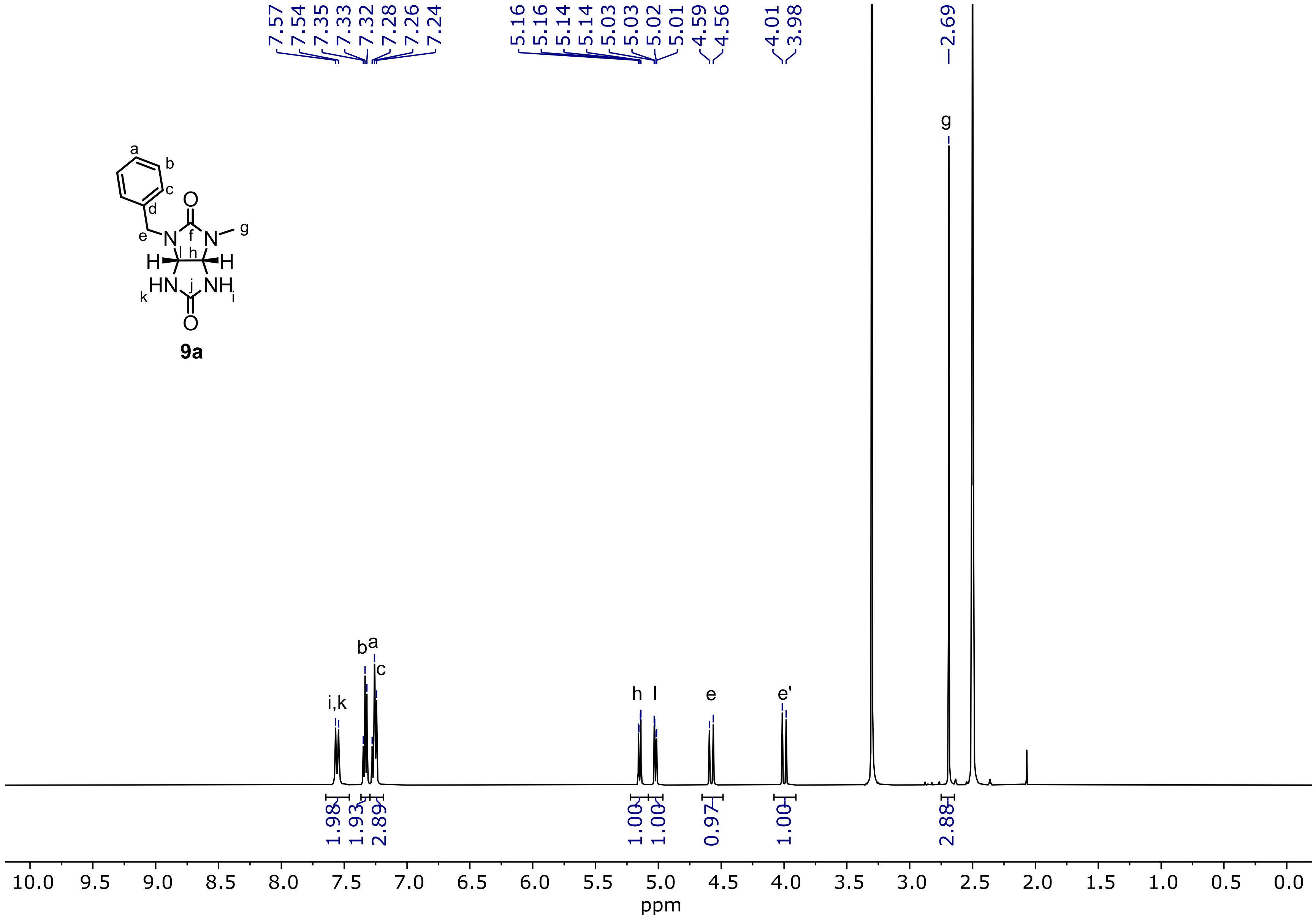

Figure S31. ^{1}H NMR spectrum (500 MHz, DMSO-d_6, 303 K) of glycoluril **9a**.

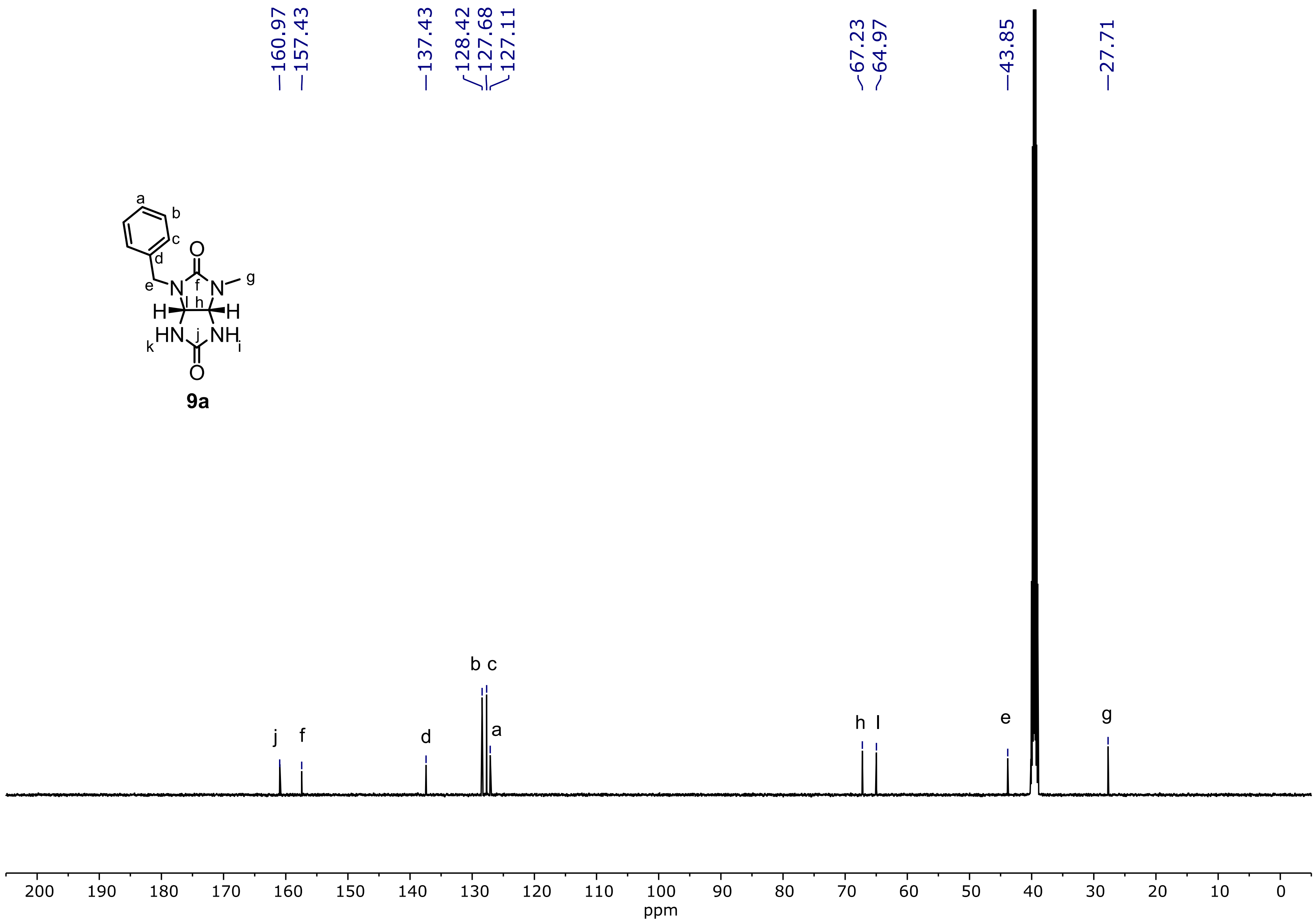

Figure S32. $^{13}C\{^{1}H\}$ NMR spectrum (126 MHz, DMSO-d_6, 303 K) of glycoluril **9a**.

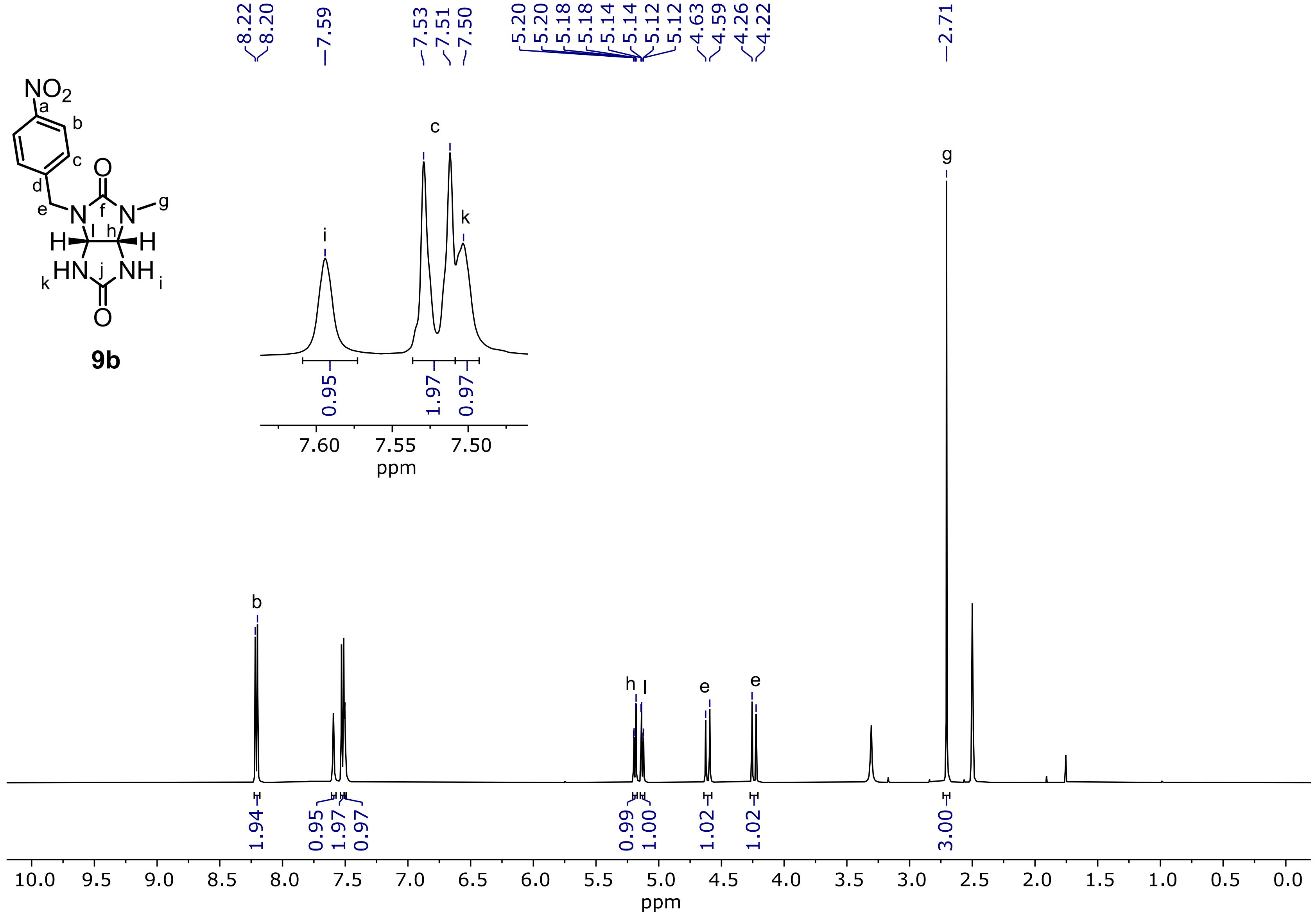

Figure S33. ^{1}H NMR spectrum (500 MHz, DMSO-d_6, 303 K) of glycoluril **9b**.

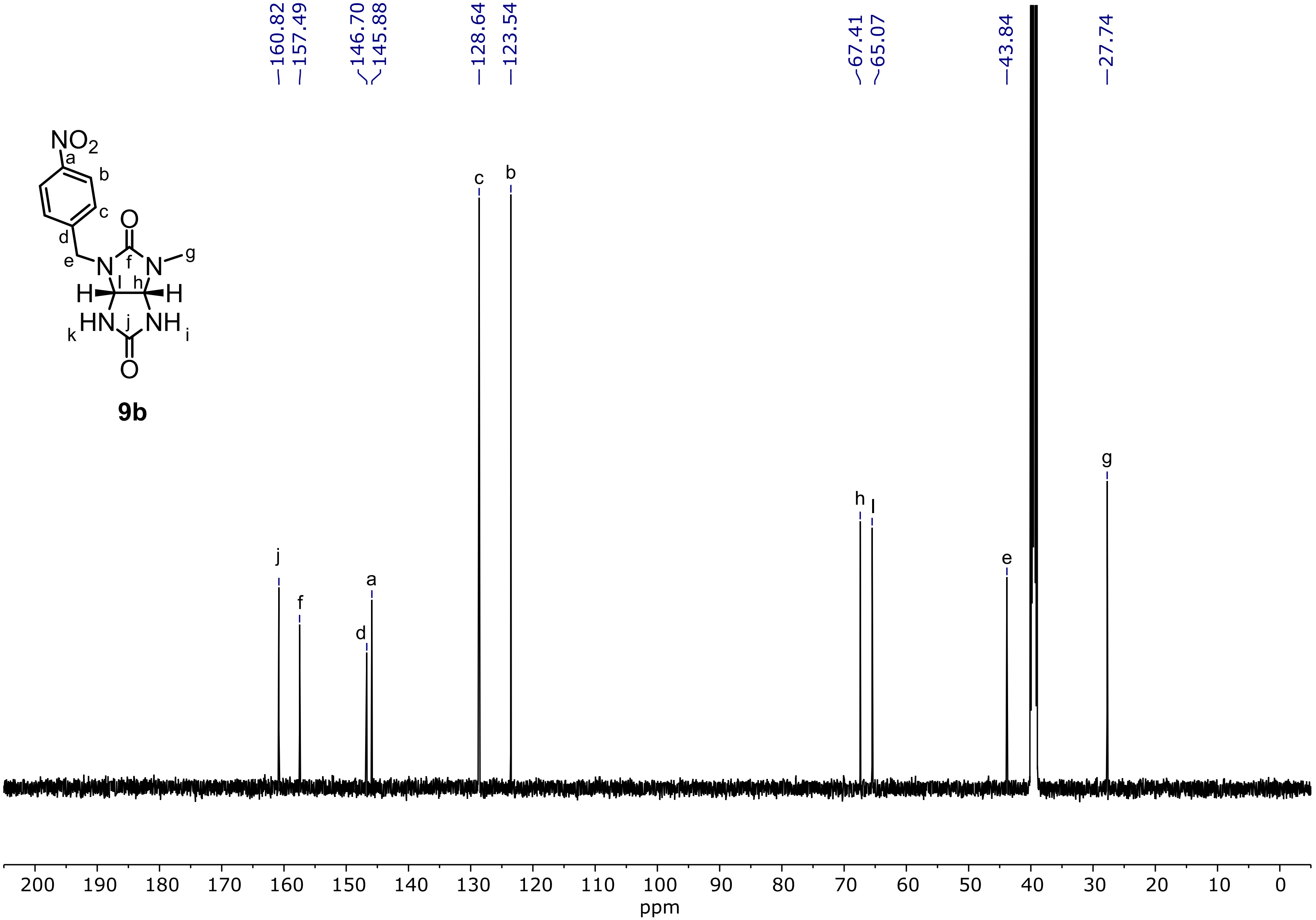

Figure S34. $^{13}C\{^{1}H\}$ NMR spectrum (126 MHz, DMSO-d_6, 303 K) of glycoluril **9b**.

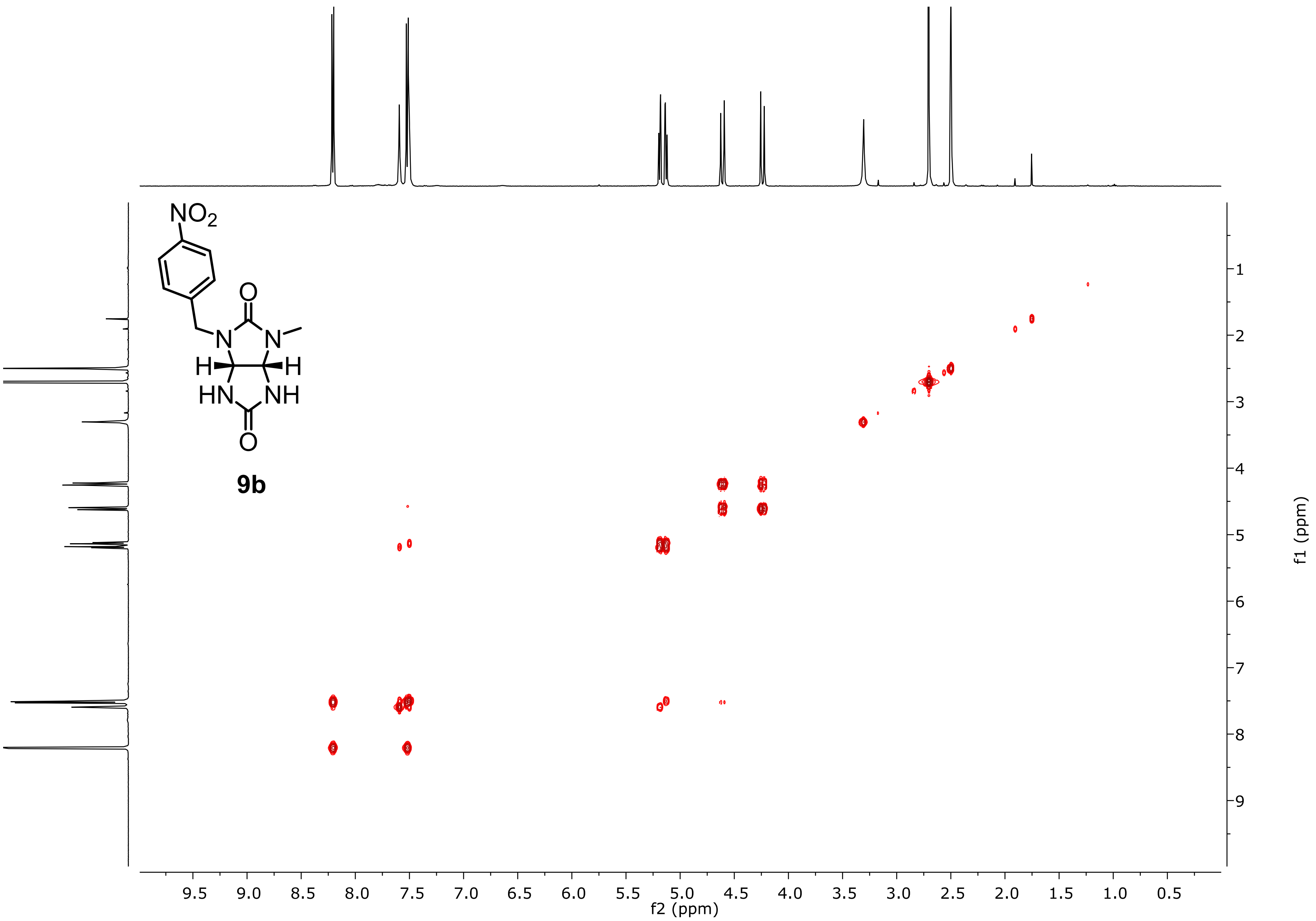

Figure S34. COSY NMR spectrum (500 MHz, DMSO-d_6, 303 K) of glycoluril **9b**.

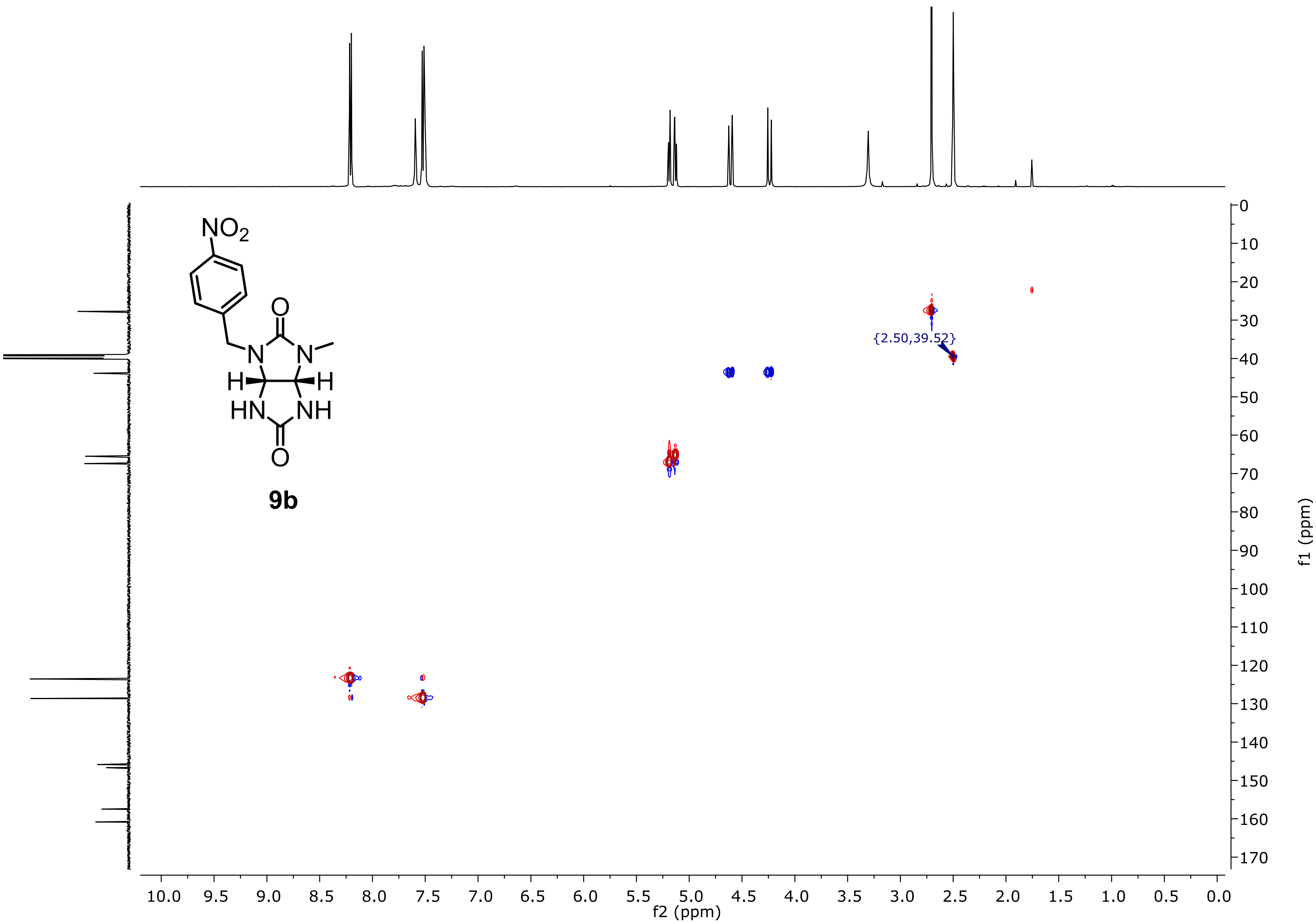

Figure S35. HSQC NMR spectrum (500 MHz, DMSO-d_6, 303 K) of glycoluril **9b**.

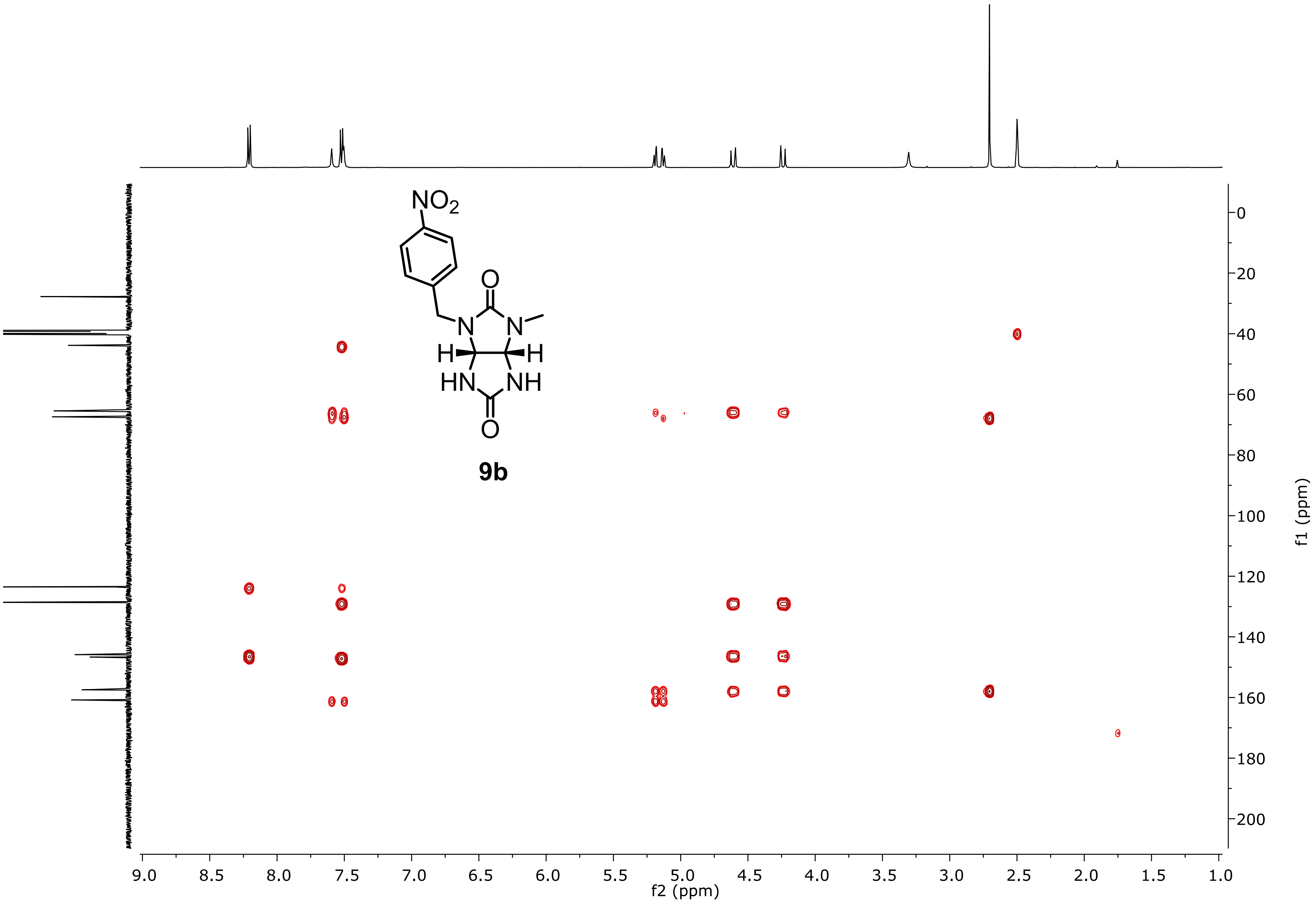

Figure S36. HMBC NMR spectrum (500 MHz, DMSO-d_6, 303 K) of glycoluril **9b**.

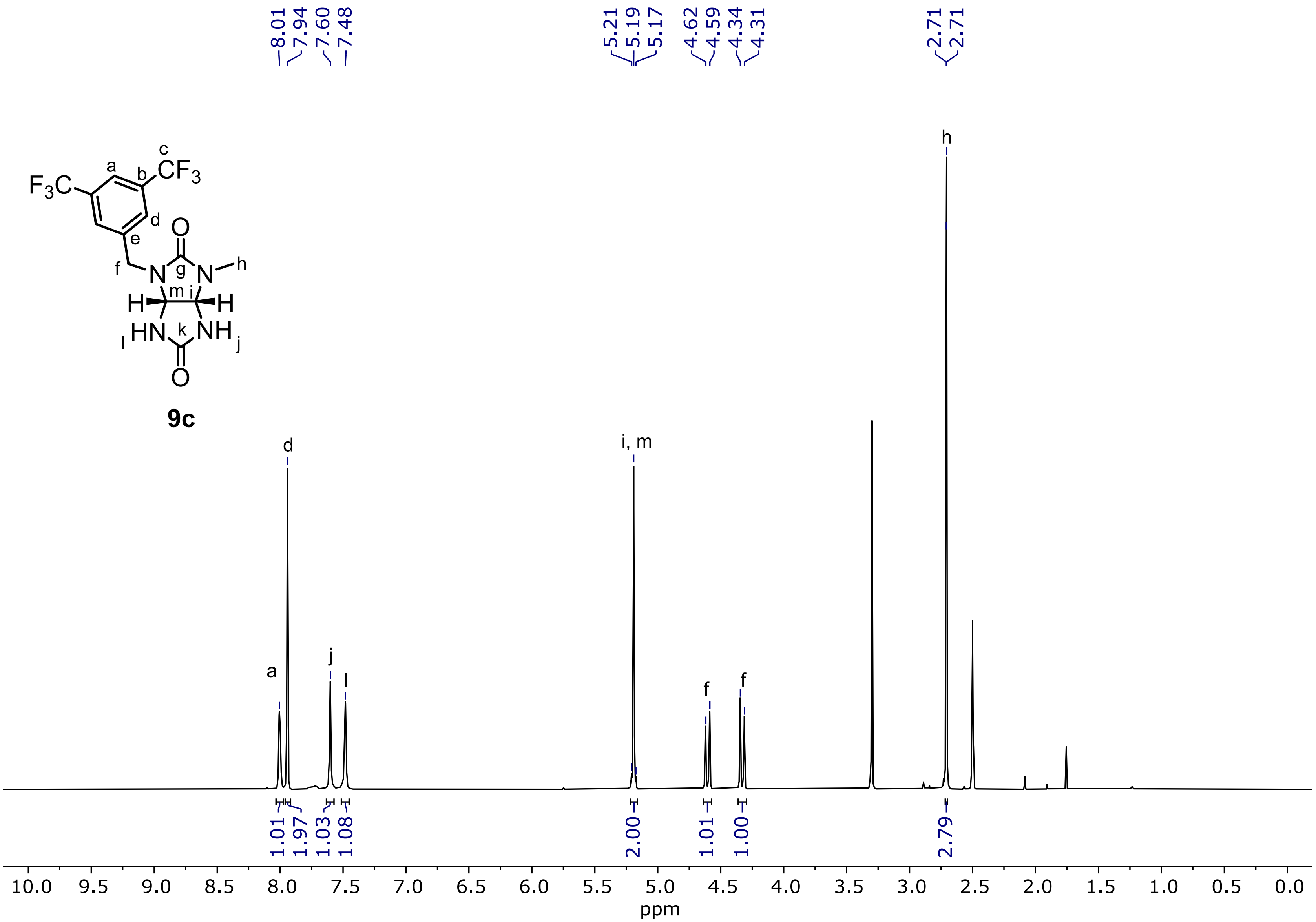

Figure S37. ^{1}H NMR spectrum (500 MHz, DMSO-d_6, 303 K) of glycoluril **9c**.

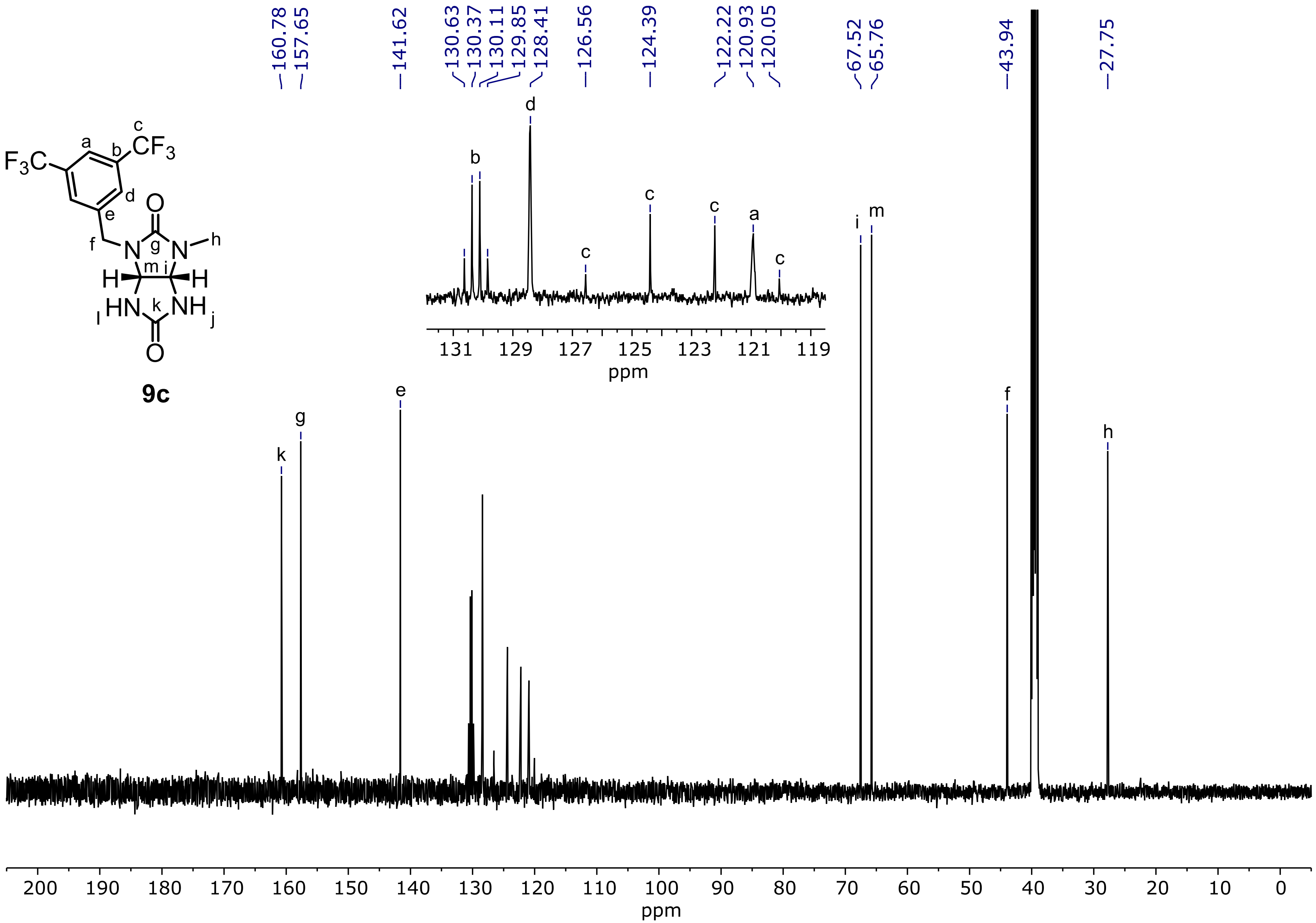

Figure S38. $^{13}C\{^{1}H\}$ NMR spectrum (126 MHz, DMSO-d_6, 303 K) of glycoluril **9c**.

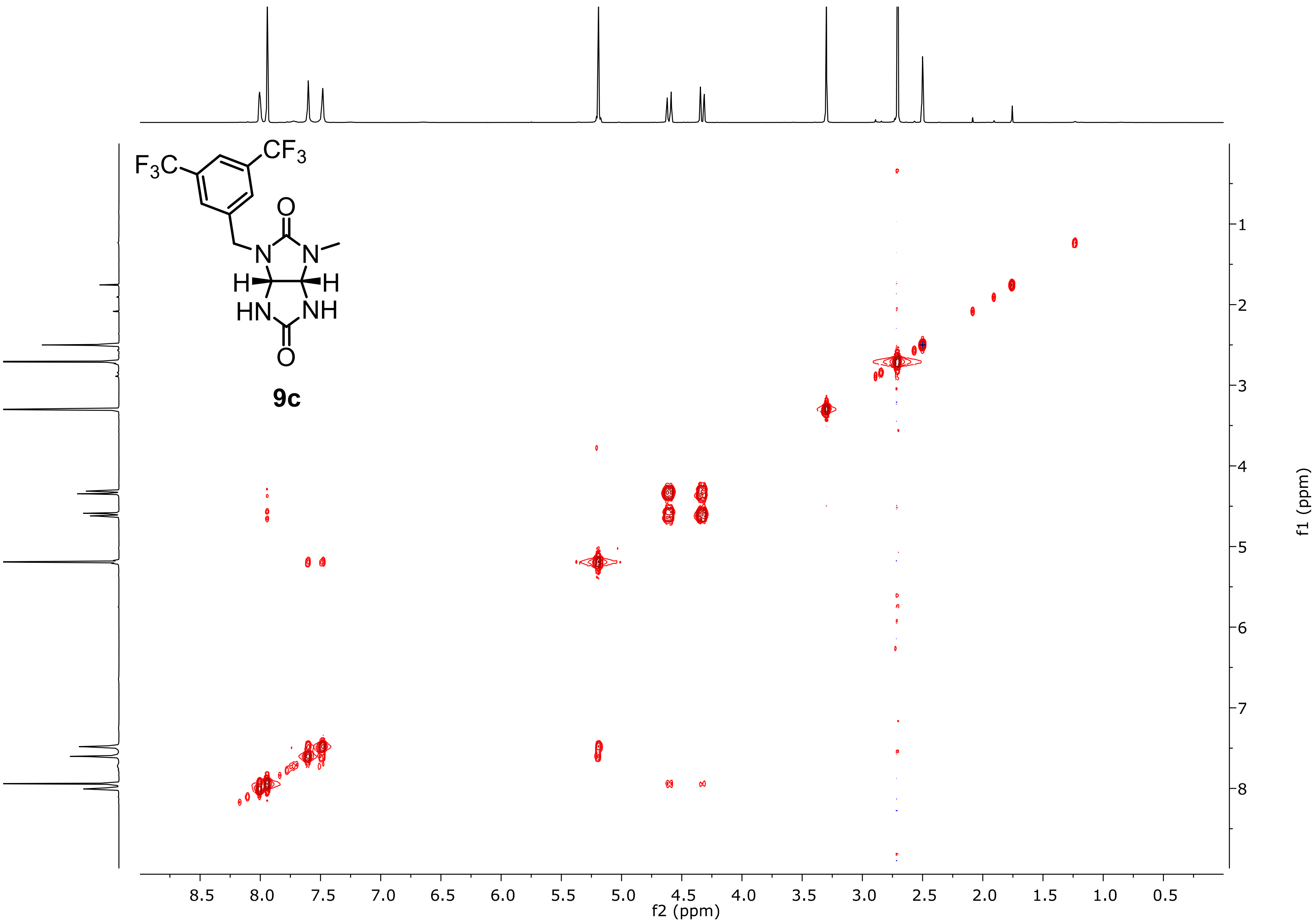

Figure S39. COSY NMR spectrum (500 MHz, DMSO-d_6, 303 K) of glycoluril **9c**.

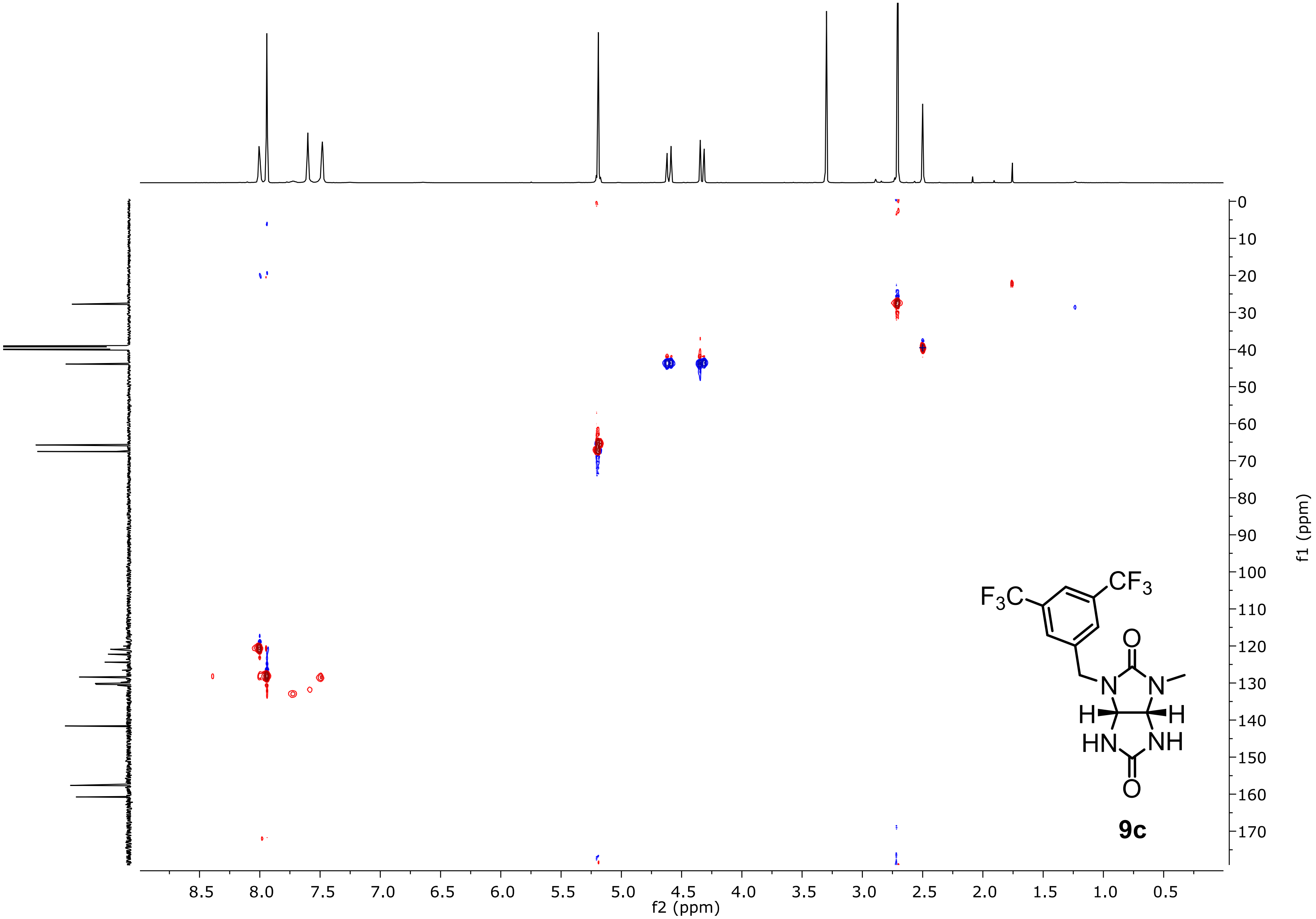

Figure S40. HSQC NMR spectrum (500 MHz, DMSO-d_6, 303 K) of glycoluril **9c**.

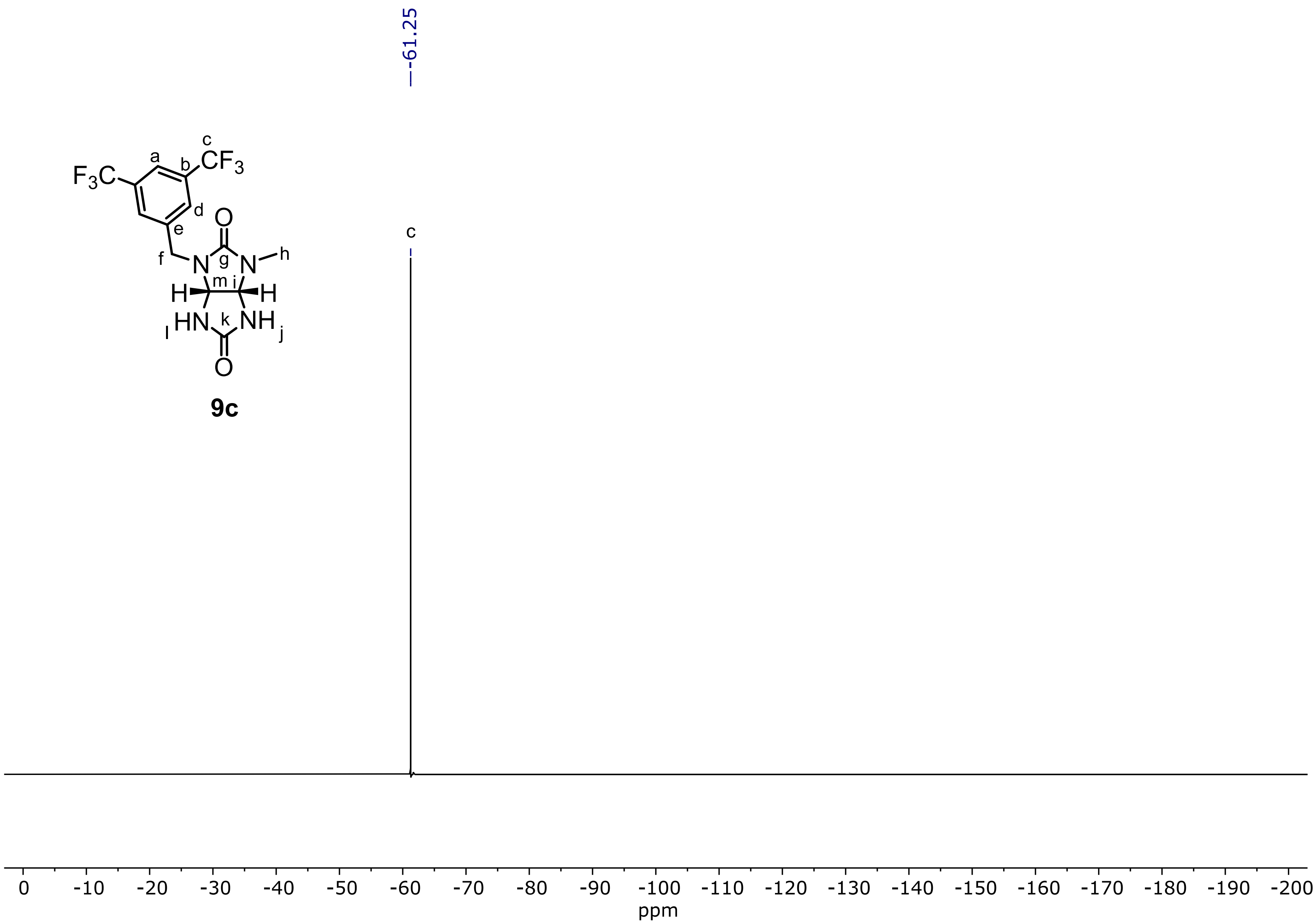

Figure S41. $^{19}F\{^{1}H\}$ NMR spectrum (471 MHz, DMSO-d_6, 303 K) of glycoluril **9c**.

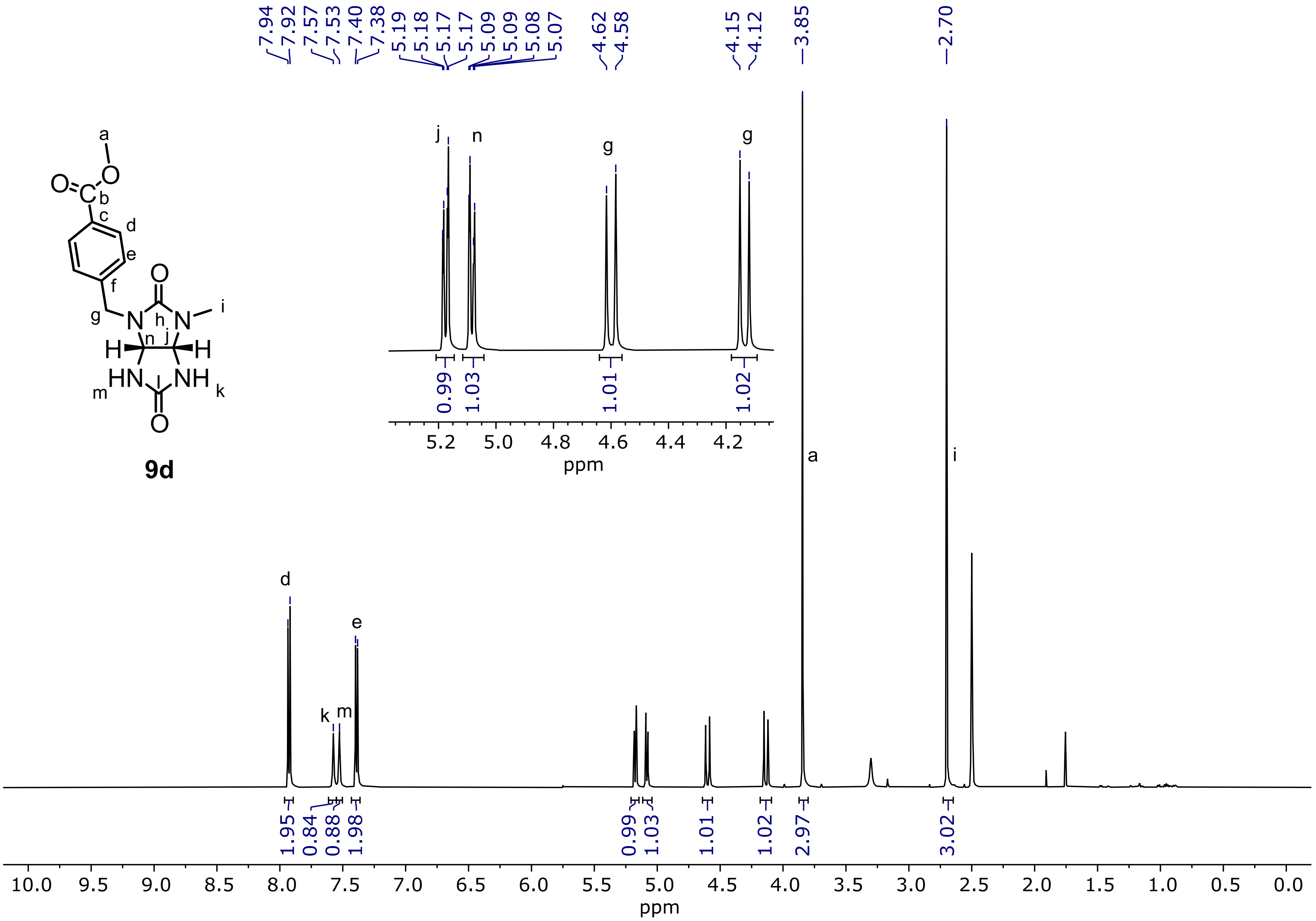

Figure S42. ^{1}H NMR spectrum (500 MHz, DMSO-d_6, 303 K) of glycoluril **9d**.

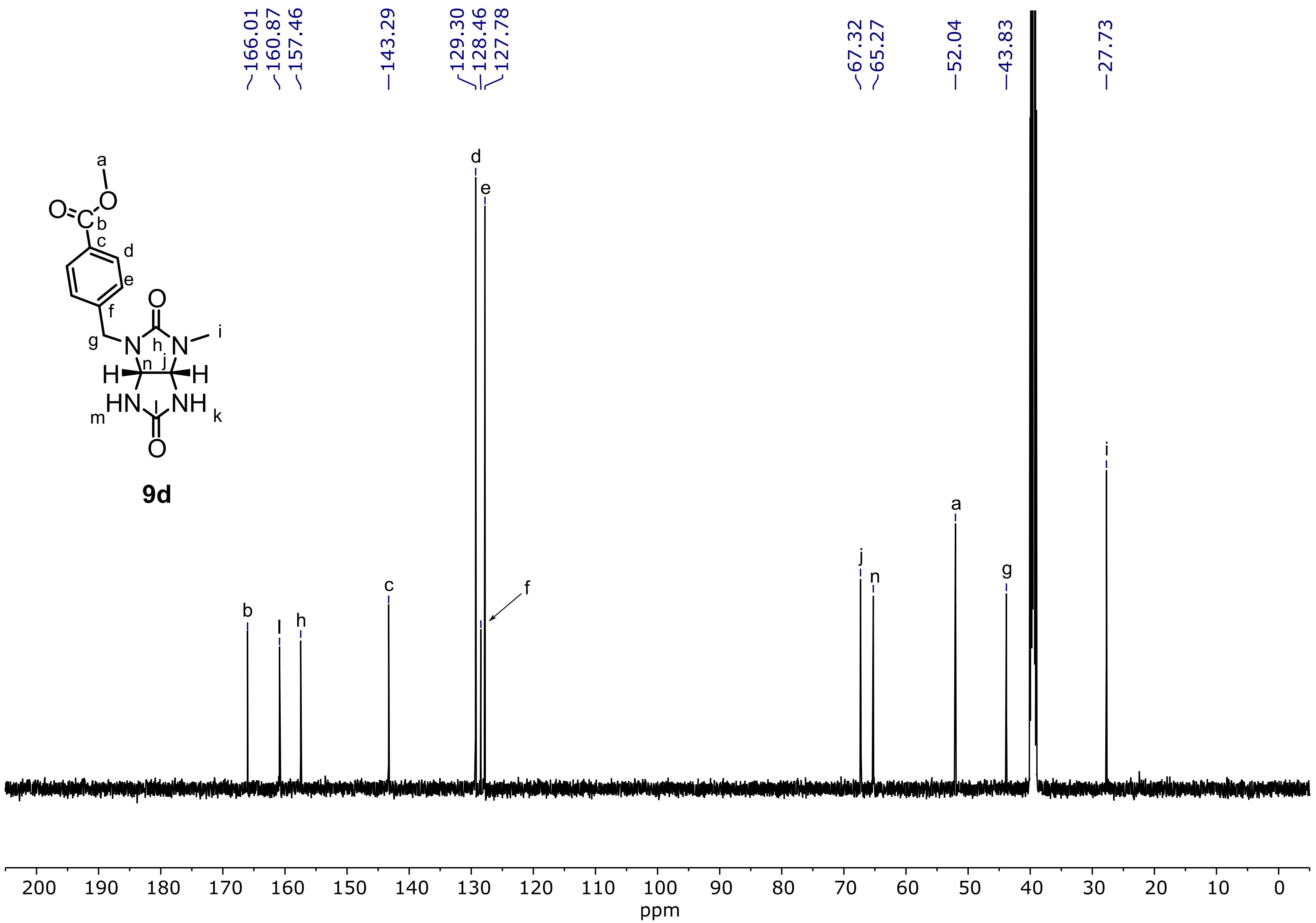

Figure S43. $^{13}C\{^{1}H\}$ NMR spectrum (126 MHz, DMSO-d_6, 303 K) of glycoluril **9d**.

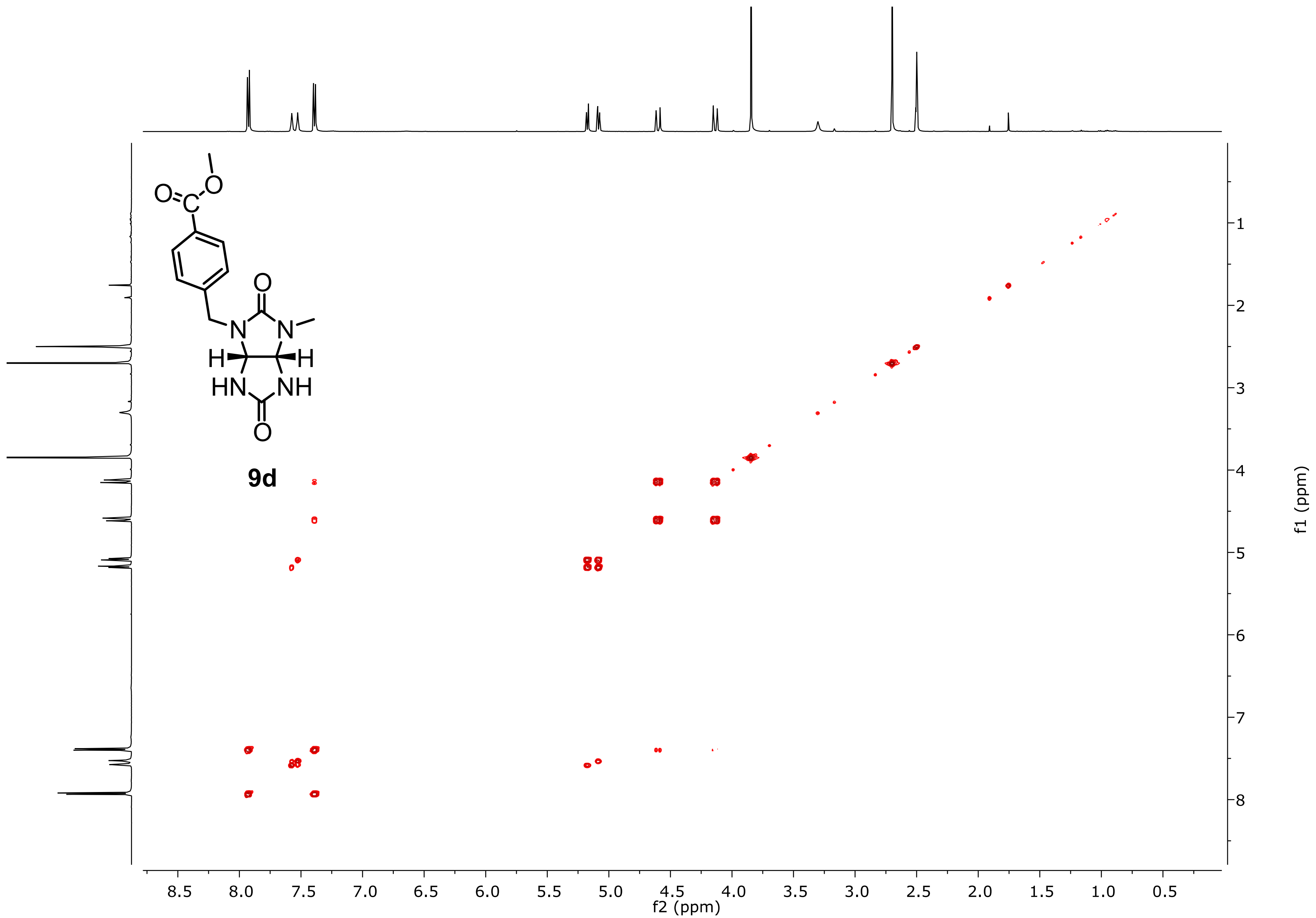

Figure S43. COSY NMR spectrum (500 MHz, DMSO-d_6, 303 K) of glycoluril **9d**.

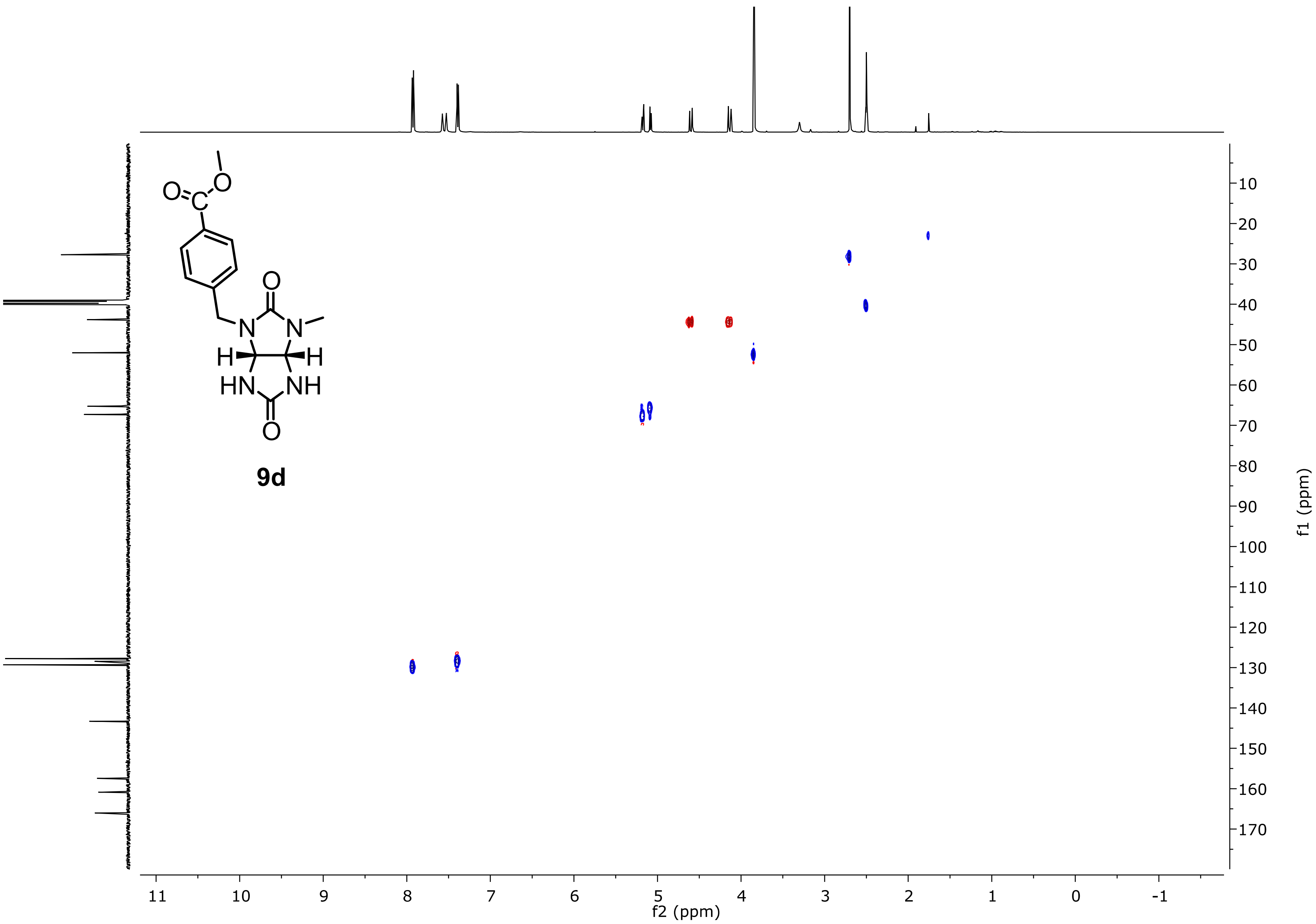

Figure S44. HSQC NMR spectrum (500 MHz, DMSO-d_6, 303 K) of glycoluril **9d**.

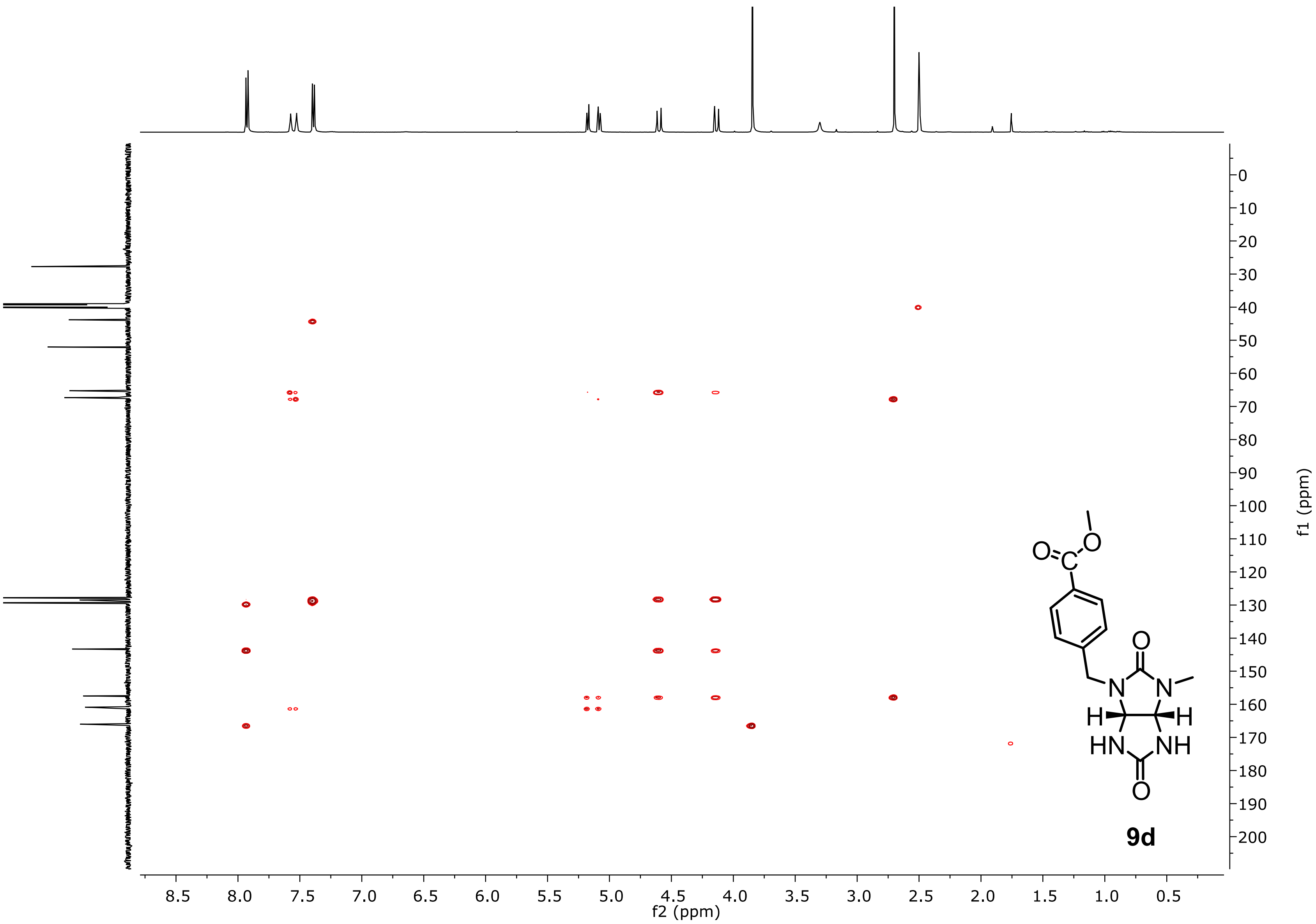

Figure S44. HMBC NMR spectrum (500 MHz, DMSO-d_6, 303 K) of glycoluril **9d**.

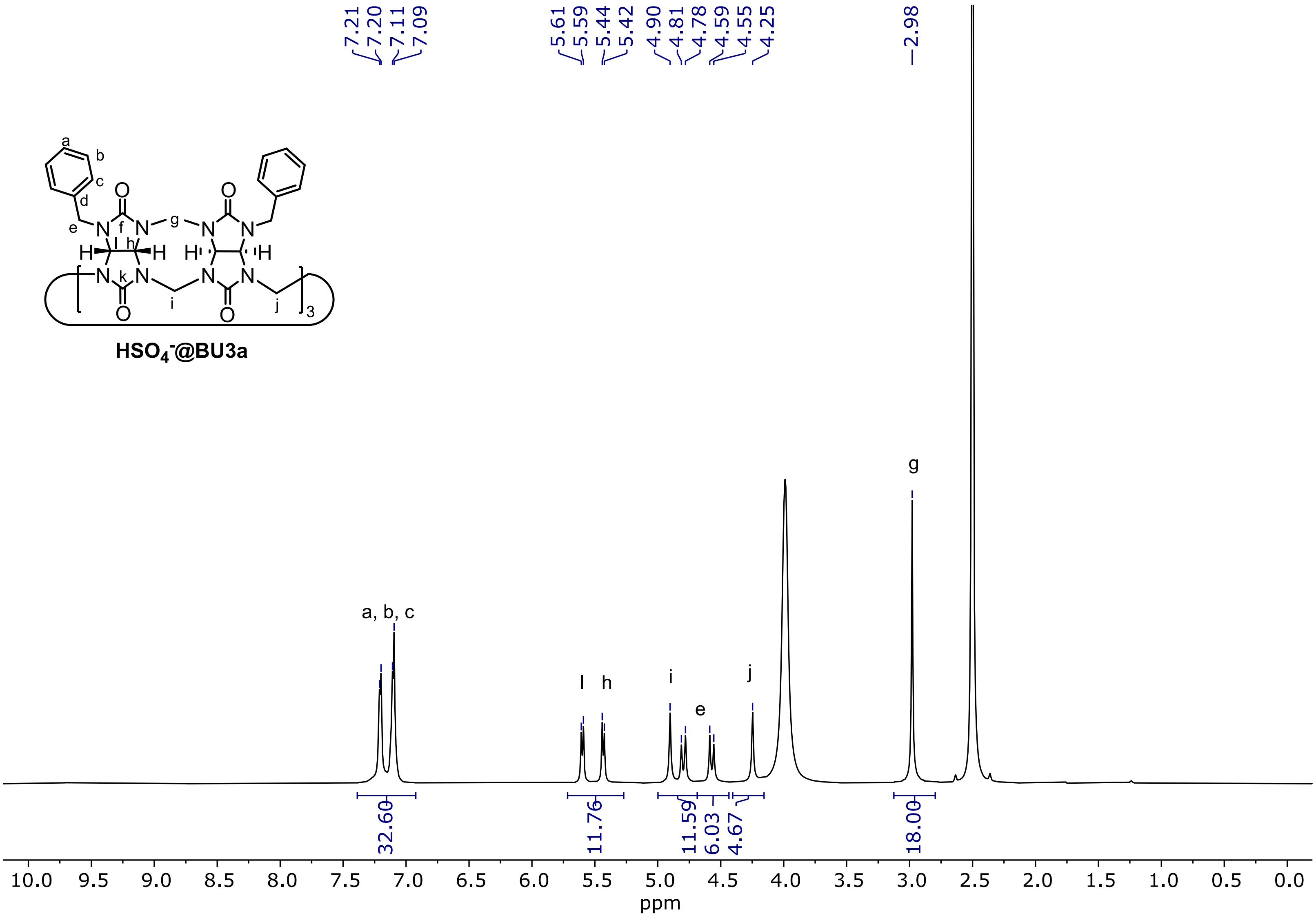

Figure S45. ^{1}H NMR spectrum (500 MHz, DMSO-d_6, 303 K) of bambus[6]uril **HSO_4^-@BU3a**.

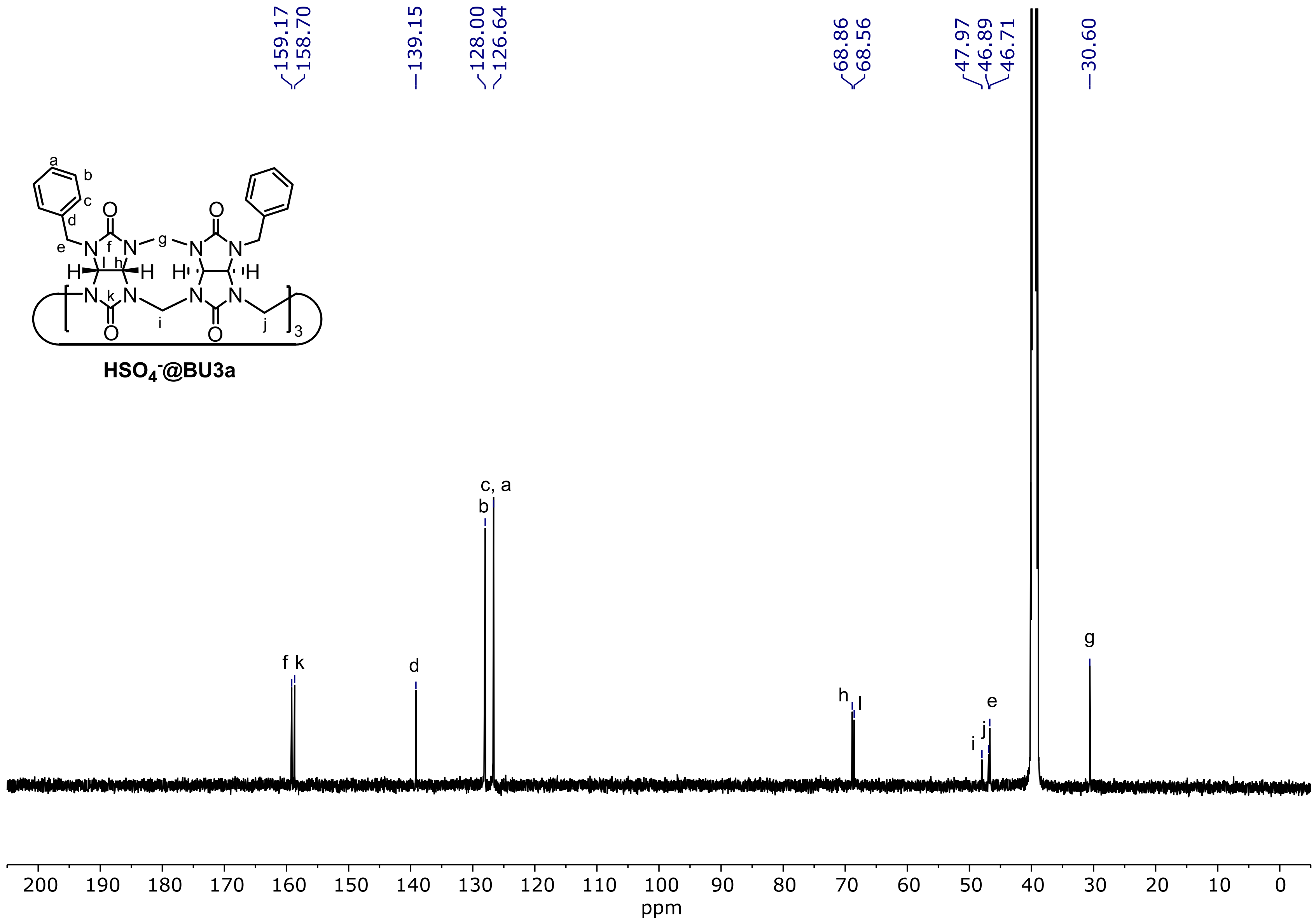

Figure S46. $^{13}C\{^{1}H\}$ NMR spectrum (126 MHz, DMSO-d_6, 303 K) of bambus[6]uril **HSO_4^-@BU3a**

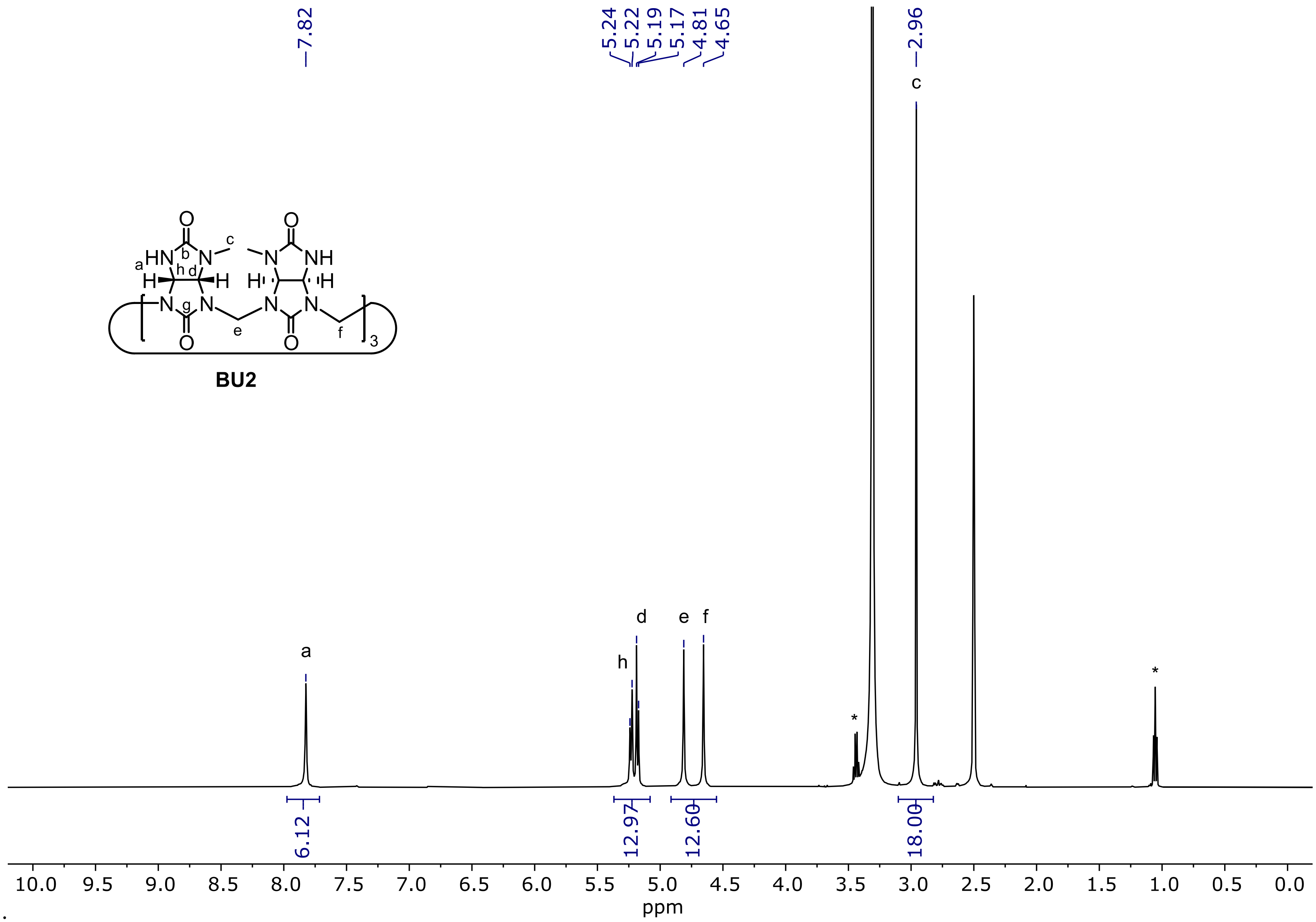

Figure S47. ^{1}H NMR spectrum (500 MHz, DMSO-d_6, 303 K) of bambus[6]uril **BU2** (*EtOH).

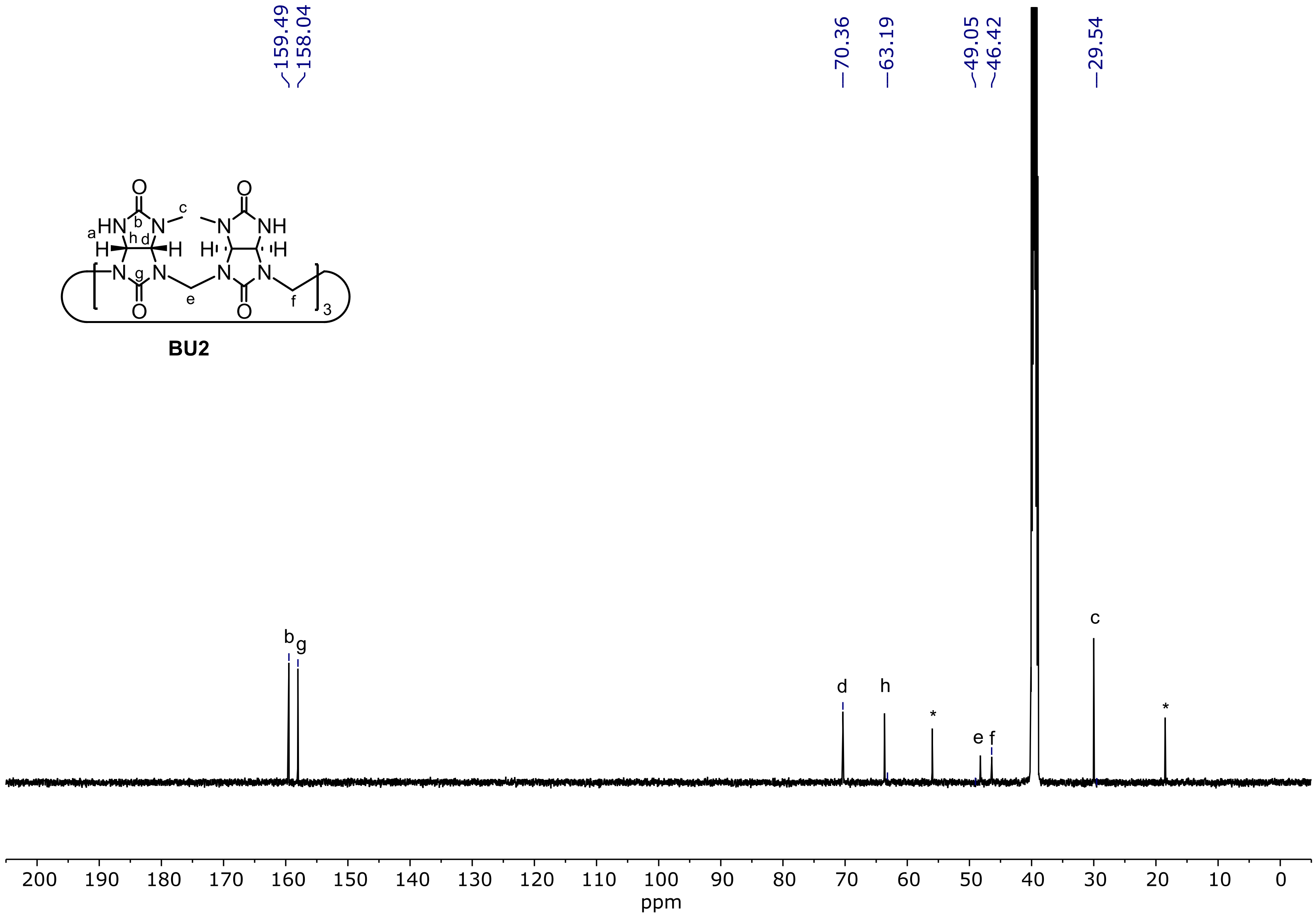

Figure S48. $^{13}C\{^{1}H\}$ NMR spectrum (126 MHz, DMSO-d_6, 303 K) of bambus[6]uril **BU2** (*EtOH).

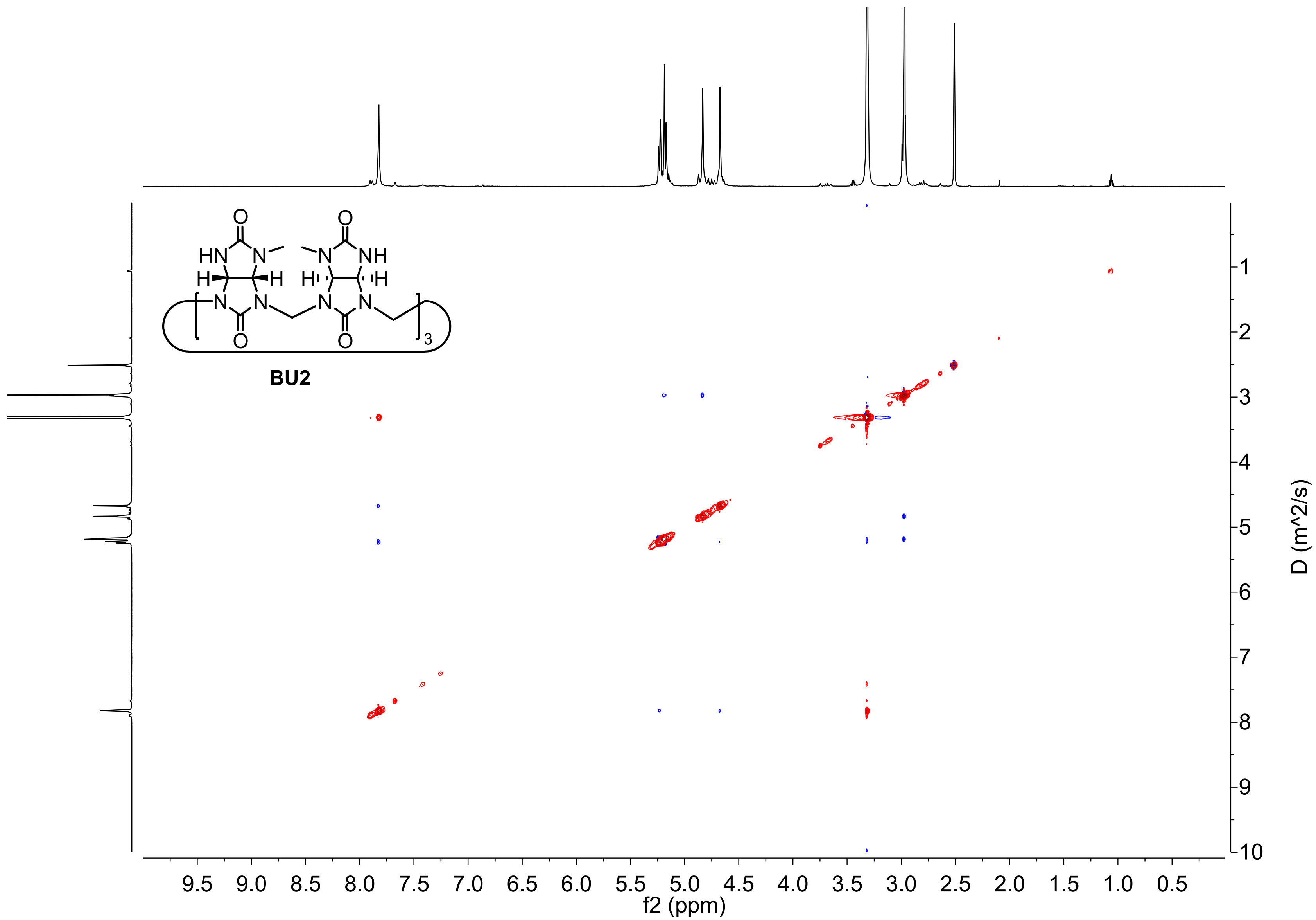

Figure S49. COSY NMR spectrum (500 MHz, DMSO-d_6, 303 K) of glycoluril **BU2**.

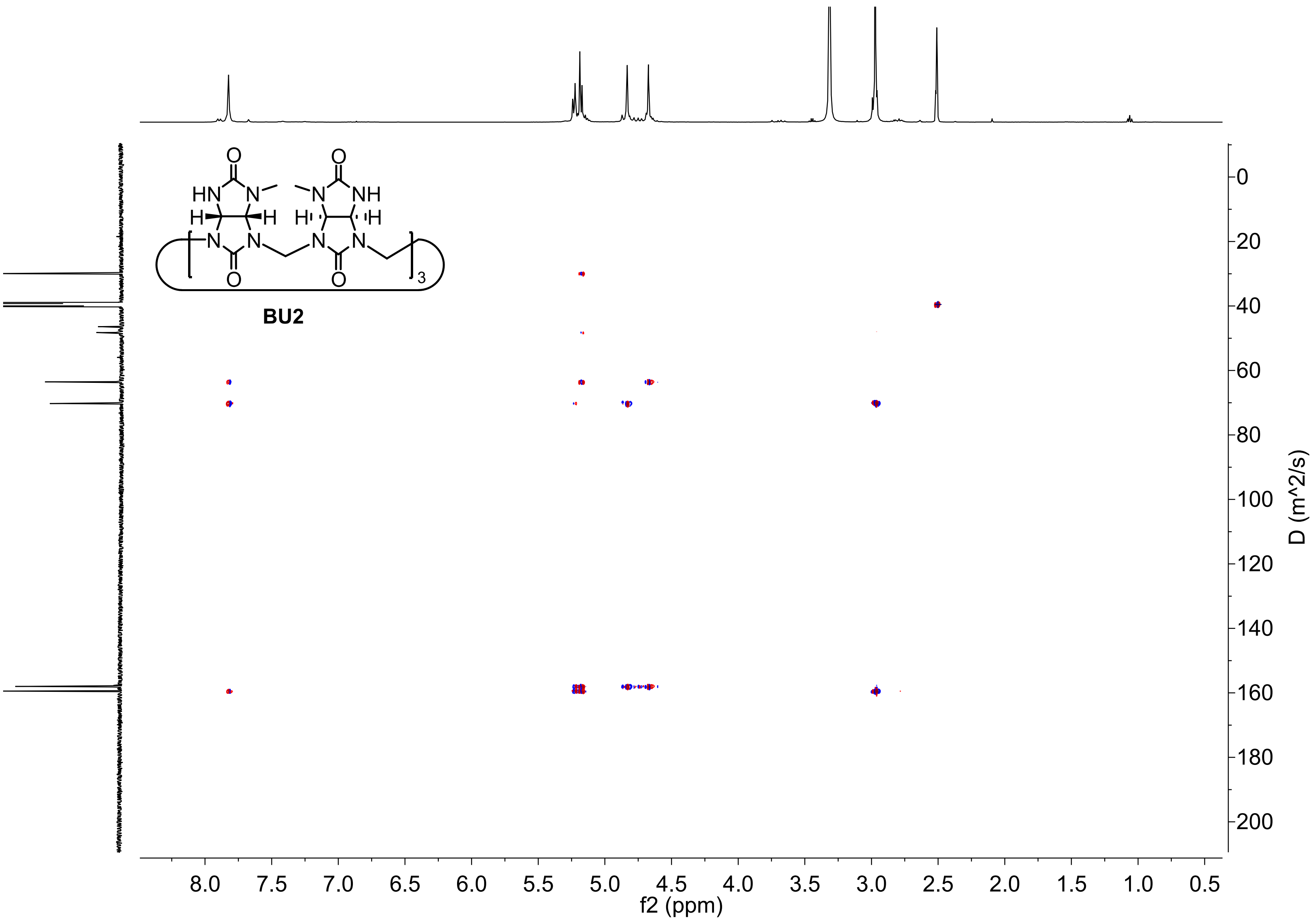

Figure S50. HSQC NMR spectrum (500 MHz, DMSO-d_6, 303 K) of glycoluril **BU2**.

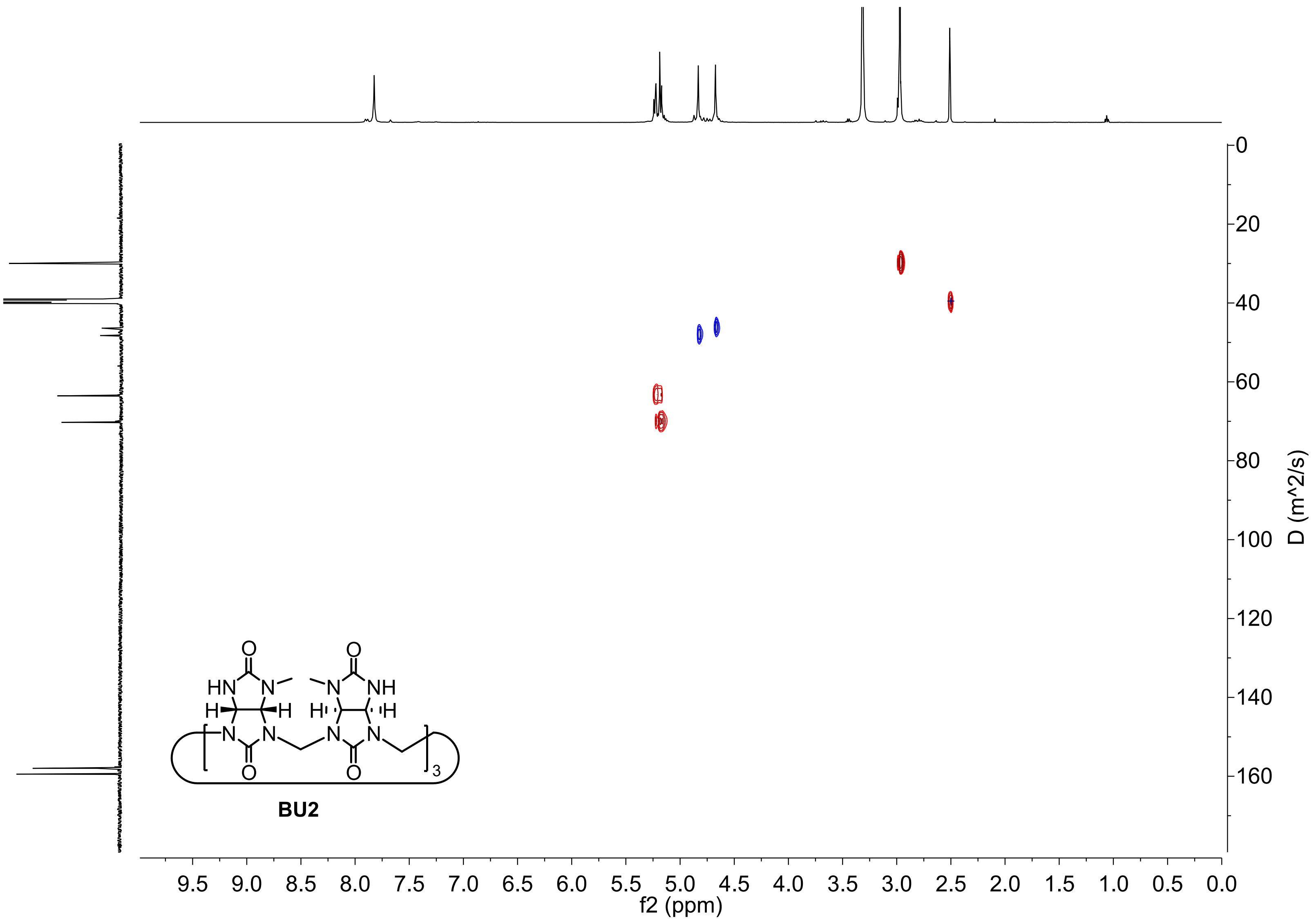

Figure S51. HMBC NMR spectrum (500 MHz, DMSO-d_6, 303 K) of glycoluril **BU2**.

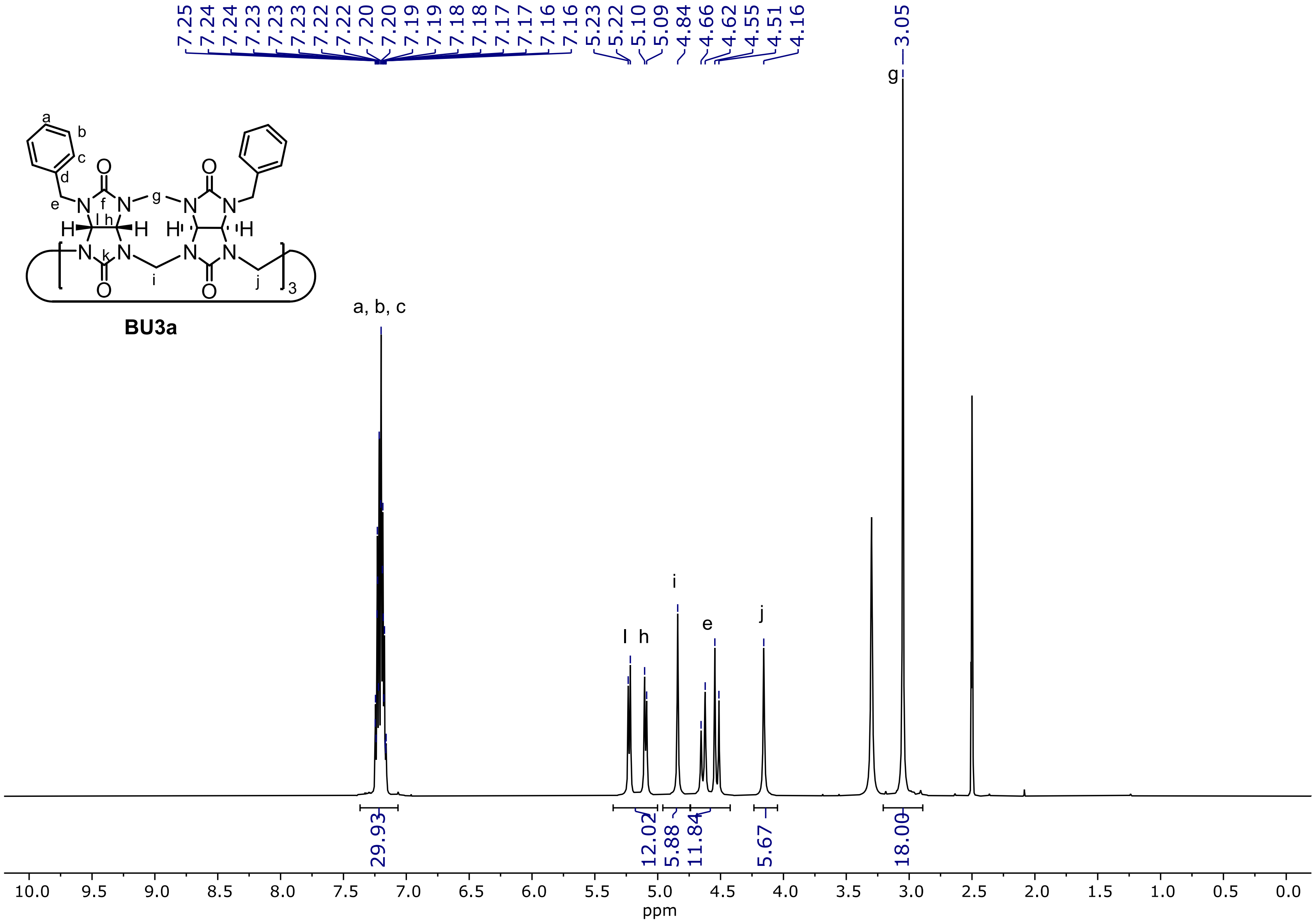

Figure S52. ^{1}H NMR spectrum (500 MHz, DMSO-d_6, 303 K) of bambus[6]uril **BU3a**.

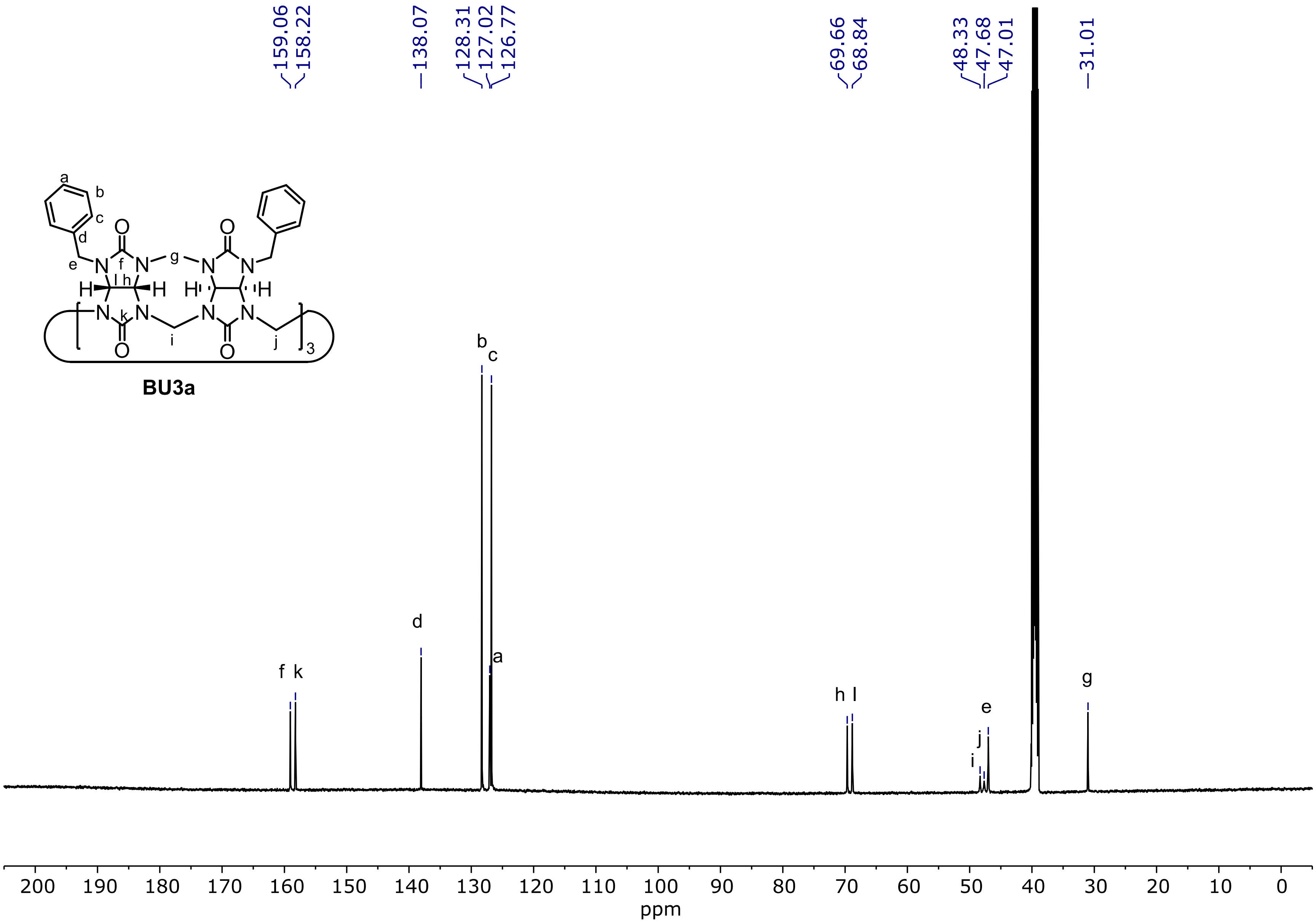

Figure S53. $^{13}C\{^{1}H\}$ NMR spectrum (126 MHz, DMSO-d_6, 303 K) of bambus[6]uril **BU3a**.

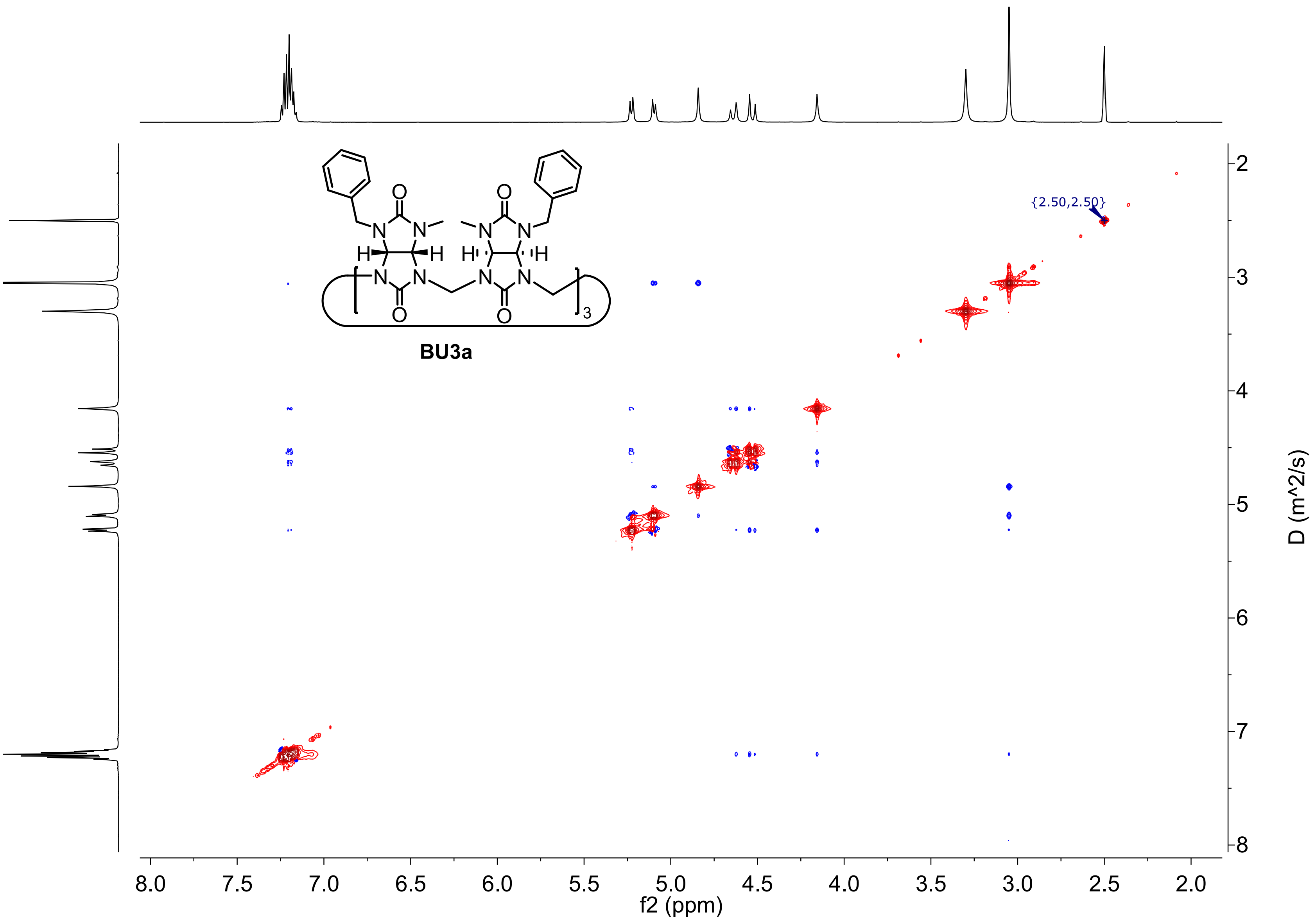

Figure S54. COSY NMR spectrum (500 MHz, DMSO-d_6, 303 K) of glycoluril **BU3a**.

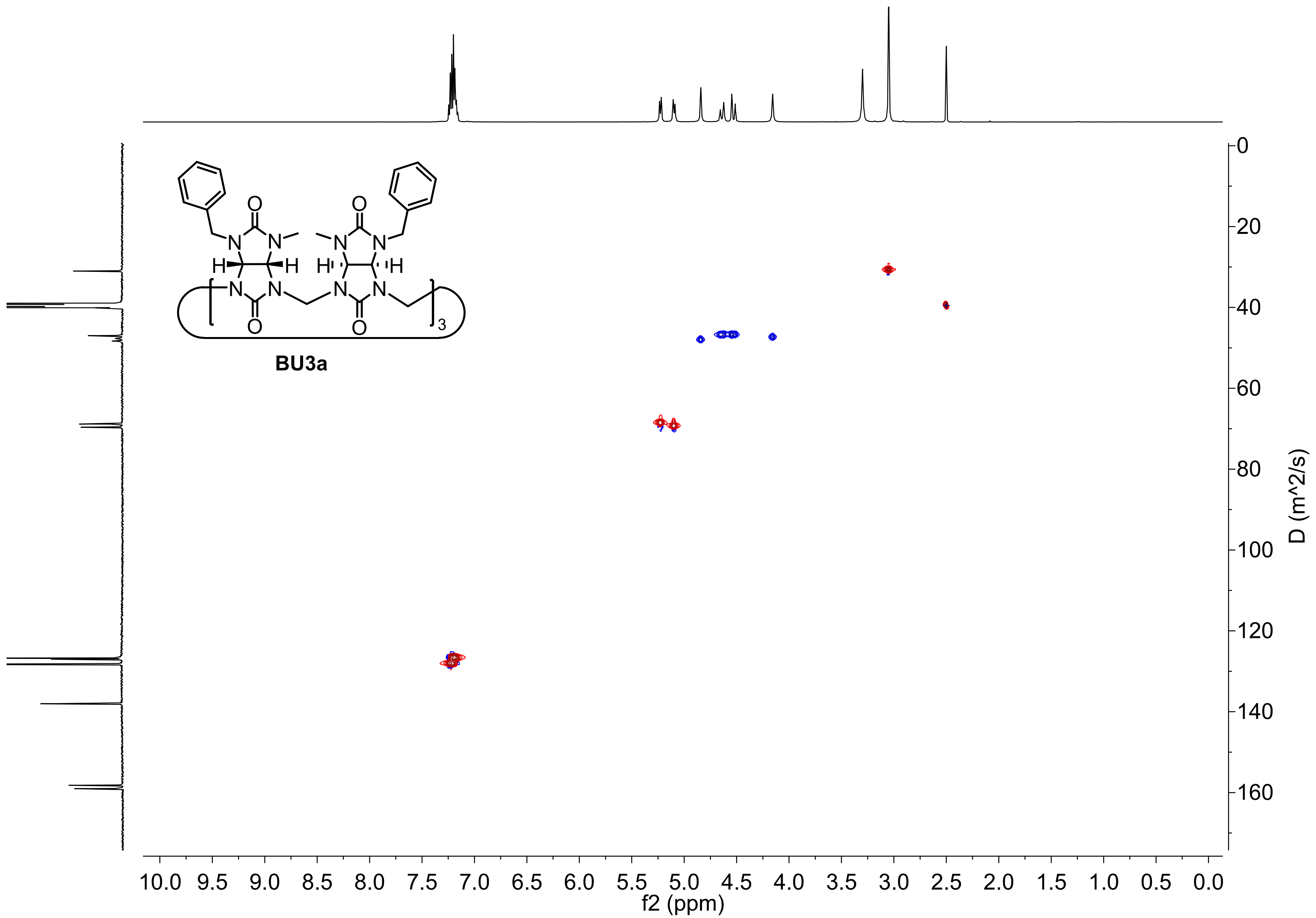

Figure S55. HSQC NMR spectrum (500 MHz, DMSO-d_6, 303 K) of glycoluril **BU3a**.

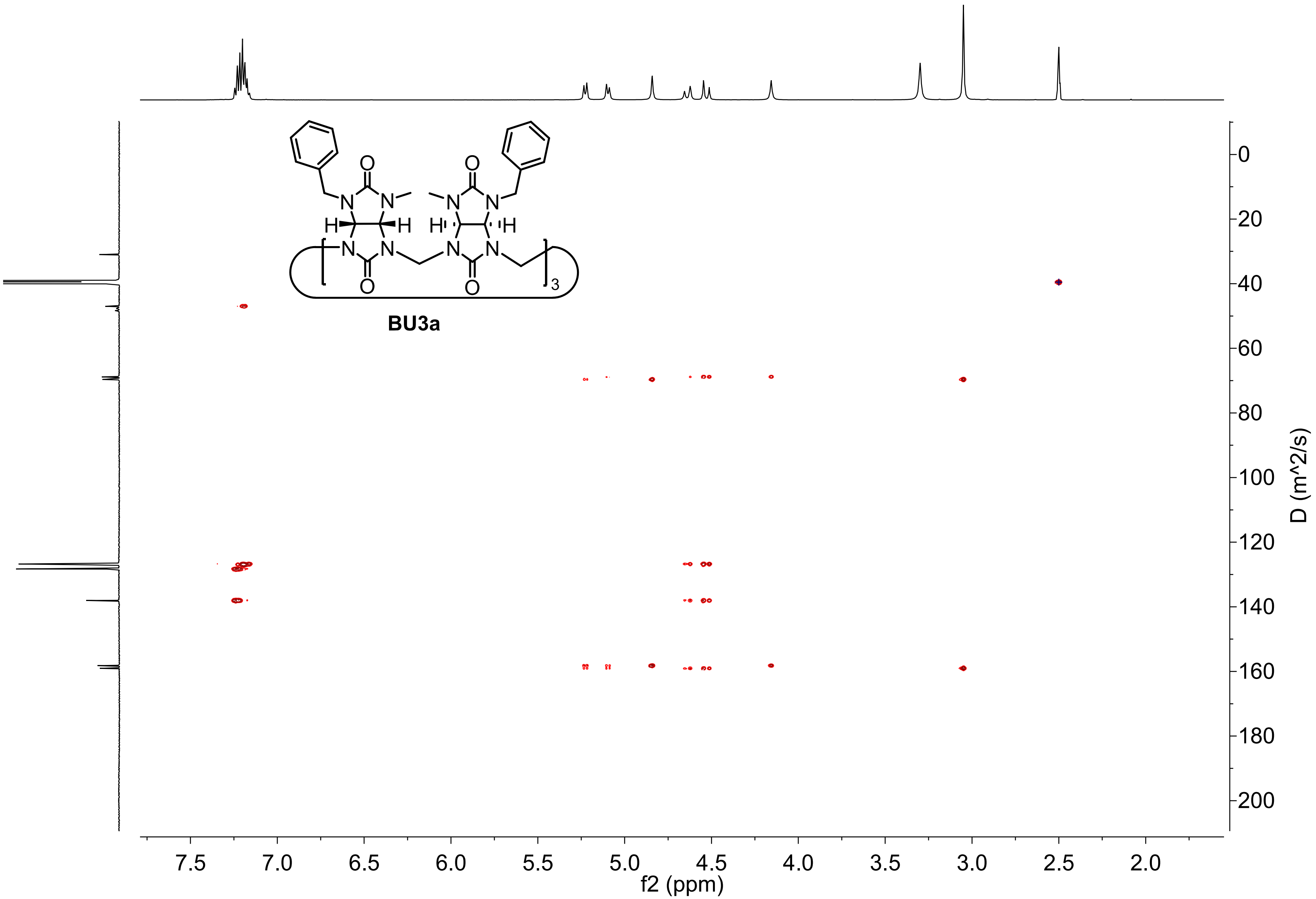

Figure S56. HMBC NMR spectrum (500 MHz, DMSO-d_6, 303 K) of glycoluril **BU3a**.

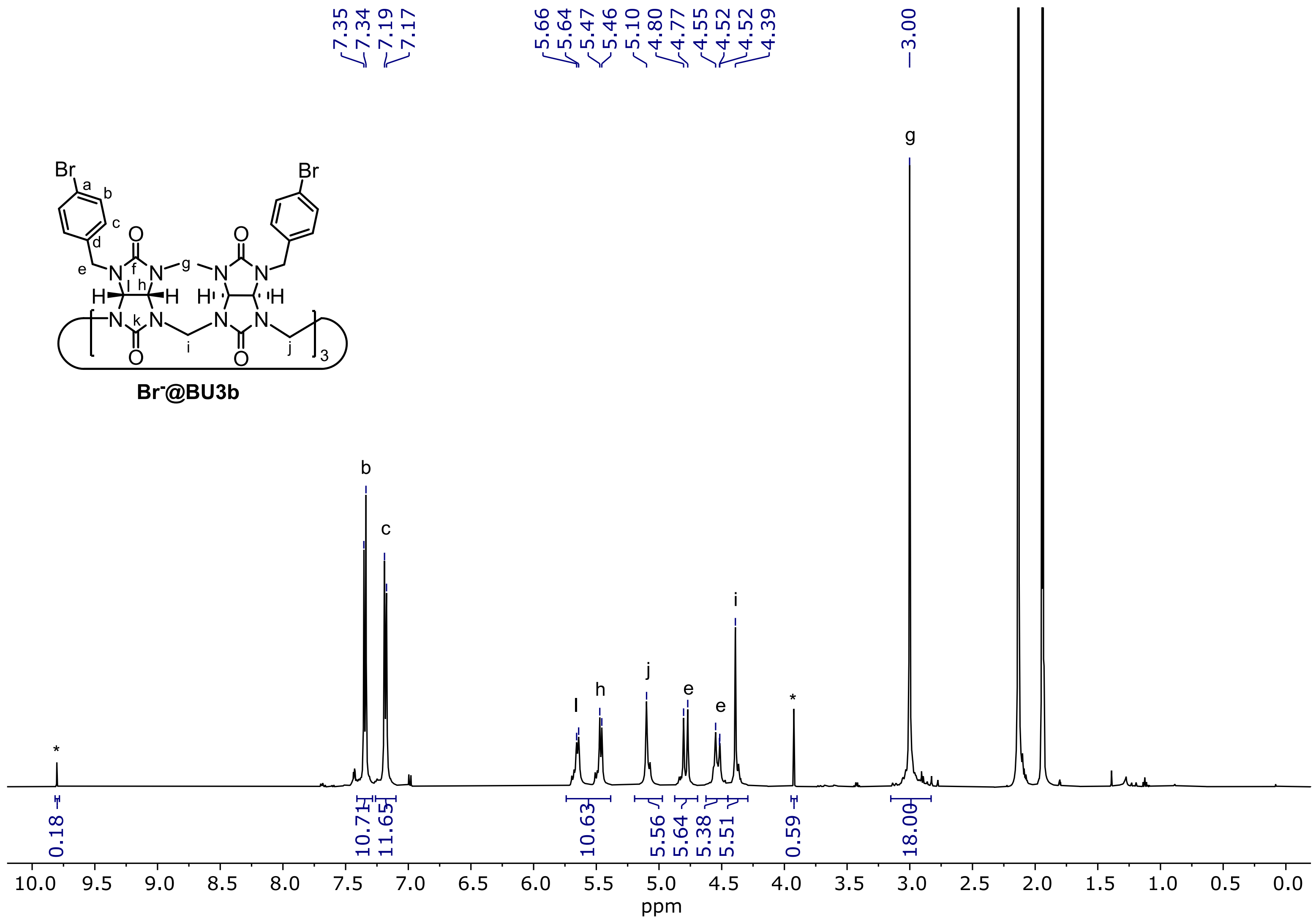

Figure S57. ^{1}H NMR spectrum (500 MHz, CD_3CN, 303 K) of bambus[6]uril **Br$^-$@BU3b** (*impurity; shoulder next to product peaks represents **BU3b** with unknown anion inside).

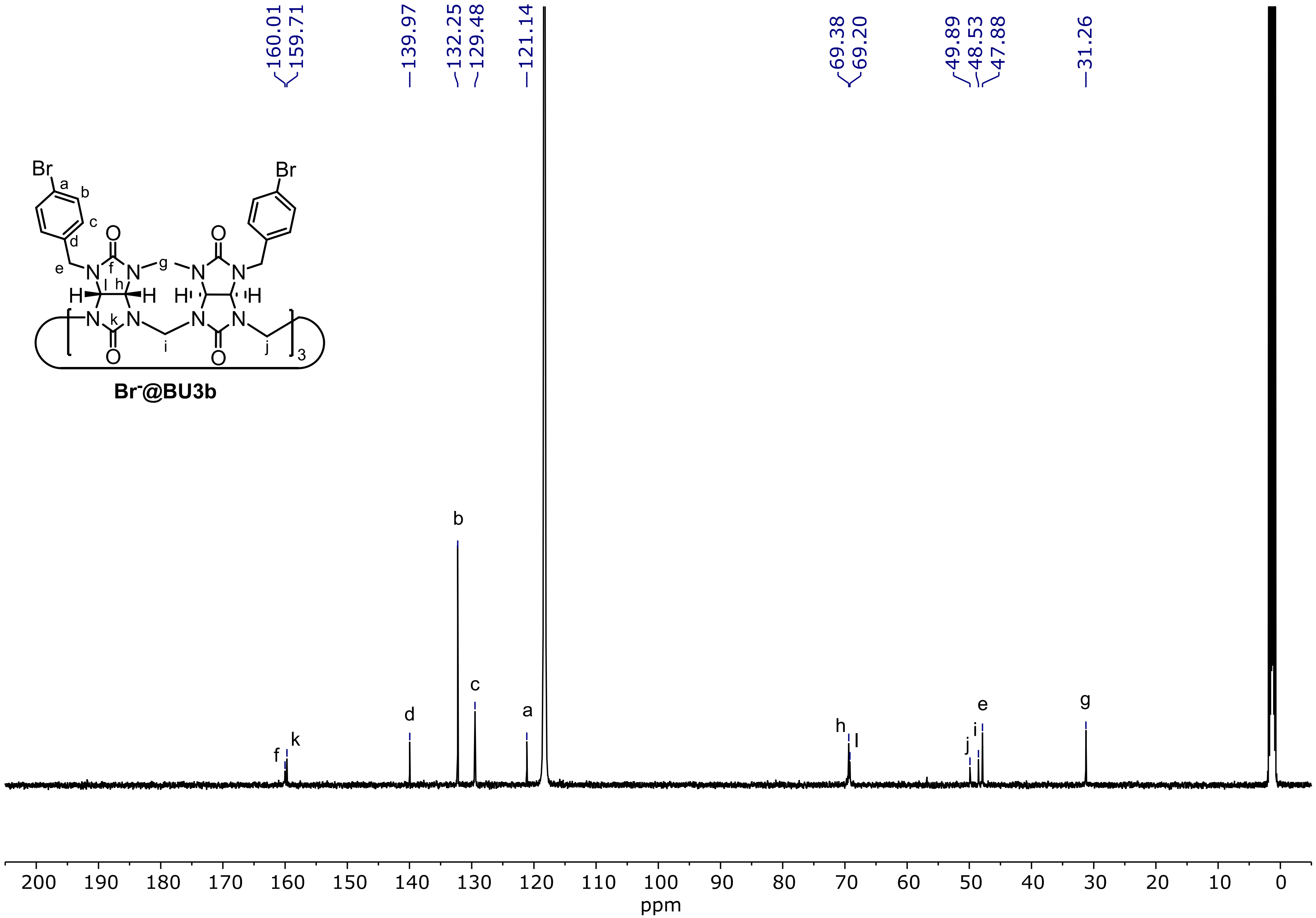

Figure S58. $^{13}C\{^{1}H\}$ NMR spectrum (126 MHz, CD_3CN, 303 K) of bambus[6]uril **Br⁻@BU3b**.

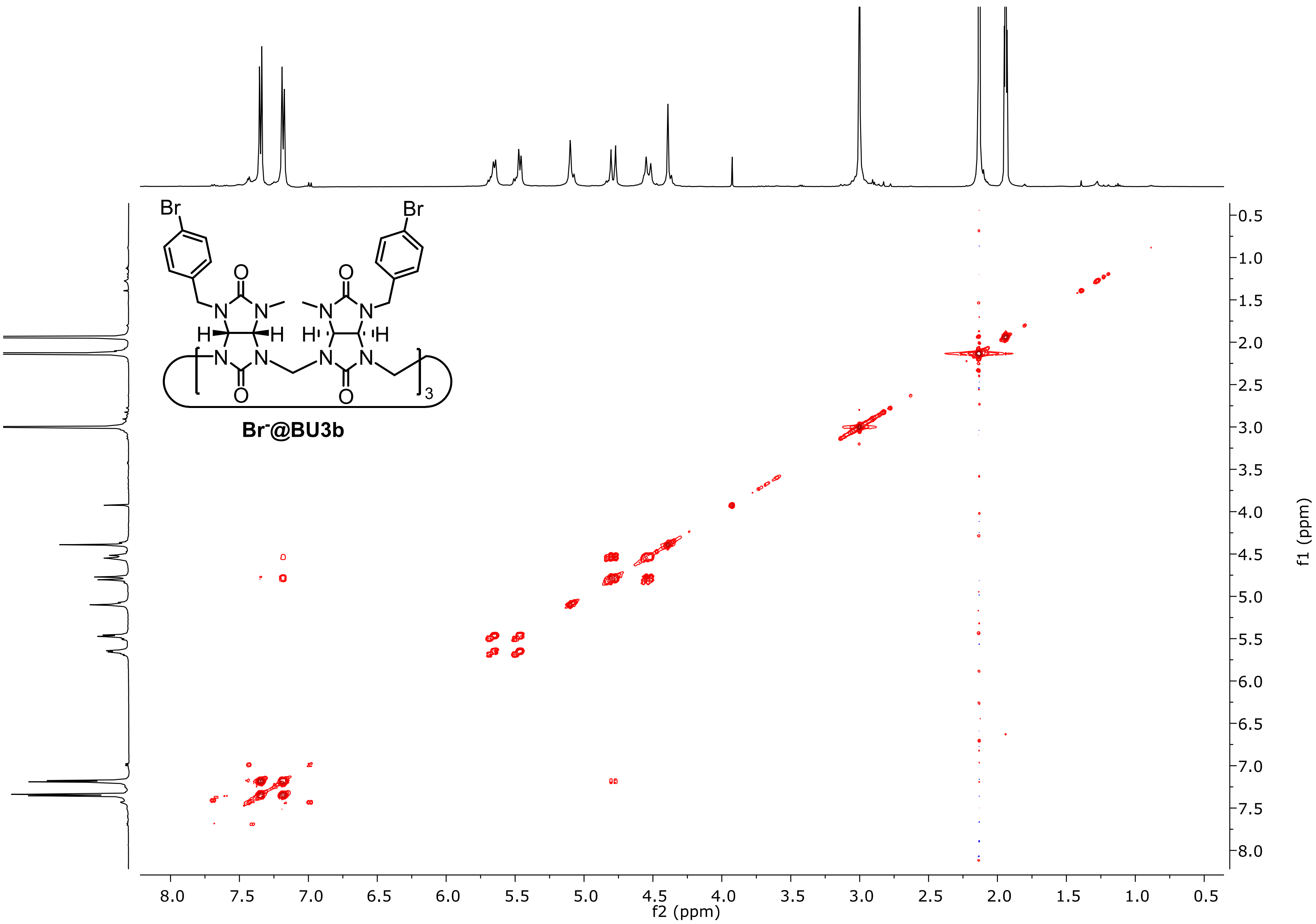

Figure S59. COSY NMR spectrum (500 MHz, CD_3CN, 303 K) of glycoluril **Br^-@BU3b**.

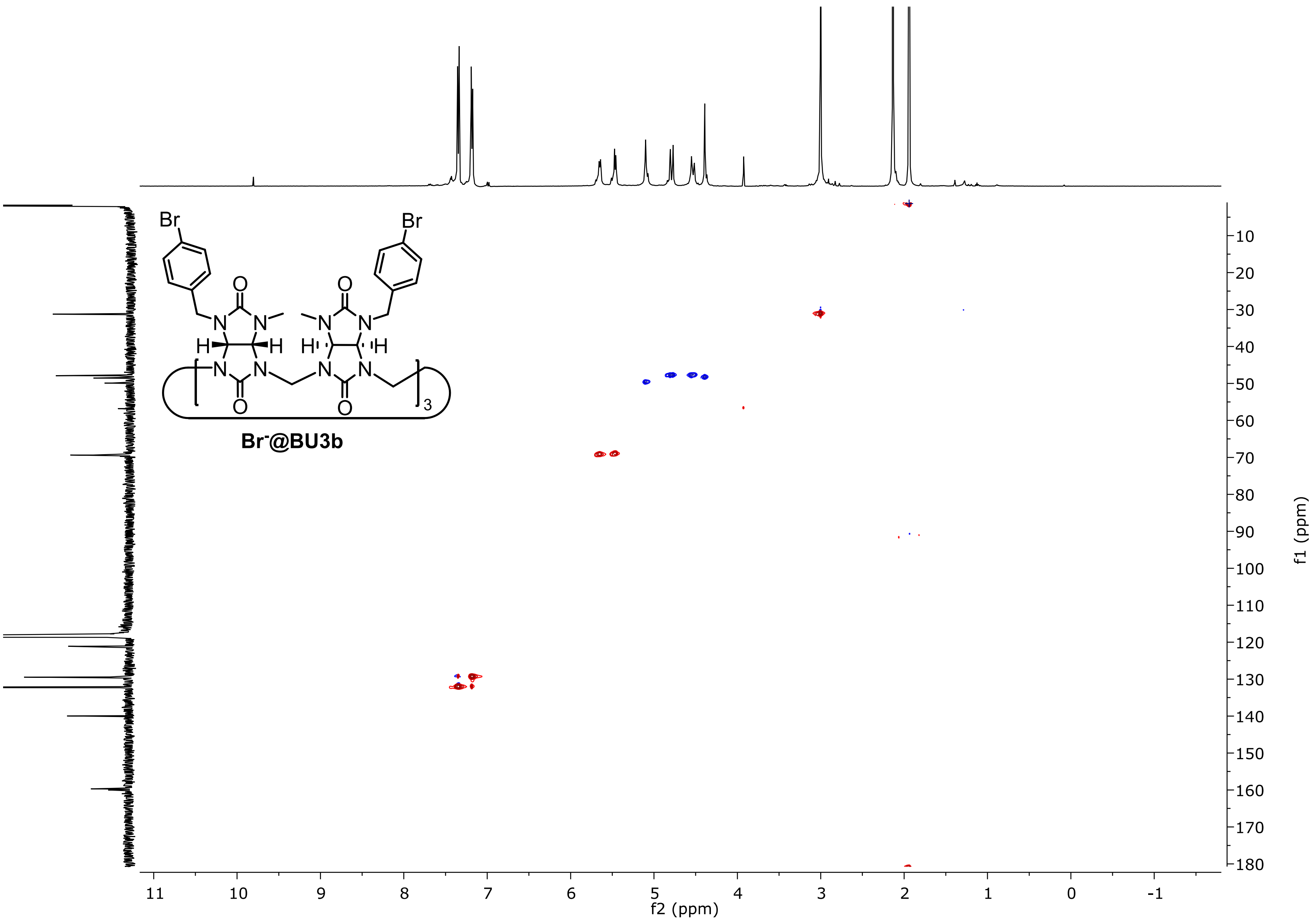

Figure S60. HSQC NMR spectrum (500 MHz, CD_3CN, 303 K) of glycoluril **Br^-@BU3b**.

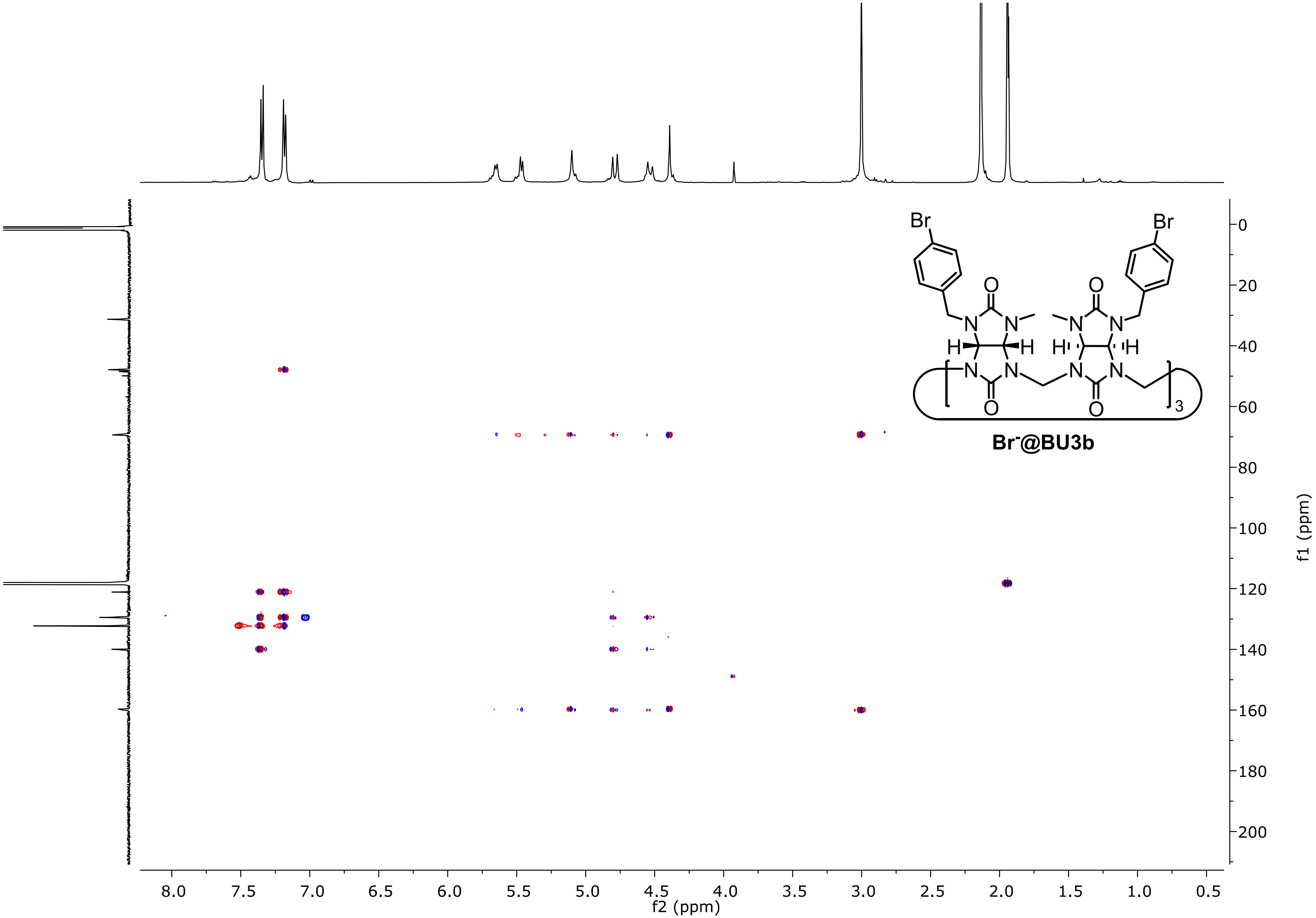

Figure S61. HMBC NMR spectrum (500 MHz, CD_3CN, 303 K) of glycoluril **Br⁻@BU3b**.

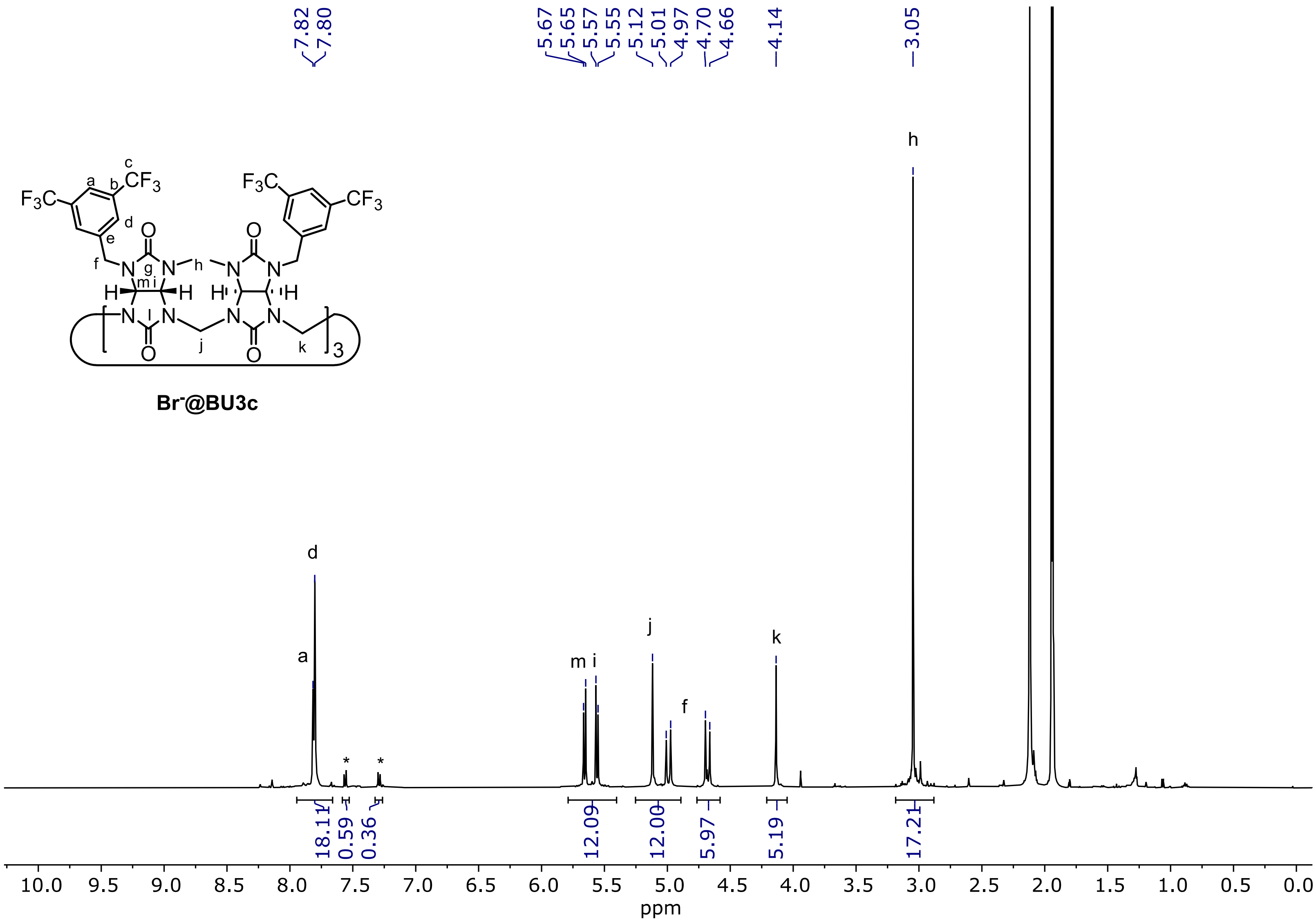

Figure S62. ^{1}H NMR spectrum (500 MHz, CD_3CN, 303 K) of bambus[6]uril **Br⁻@BU3c** (*impurity).

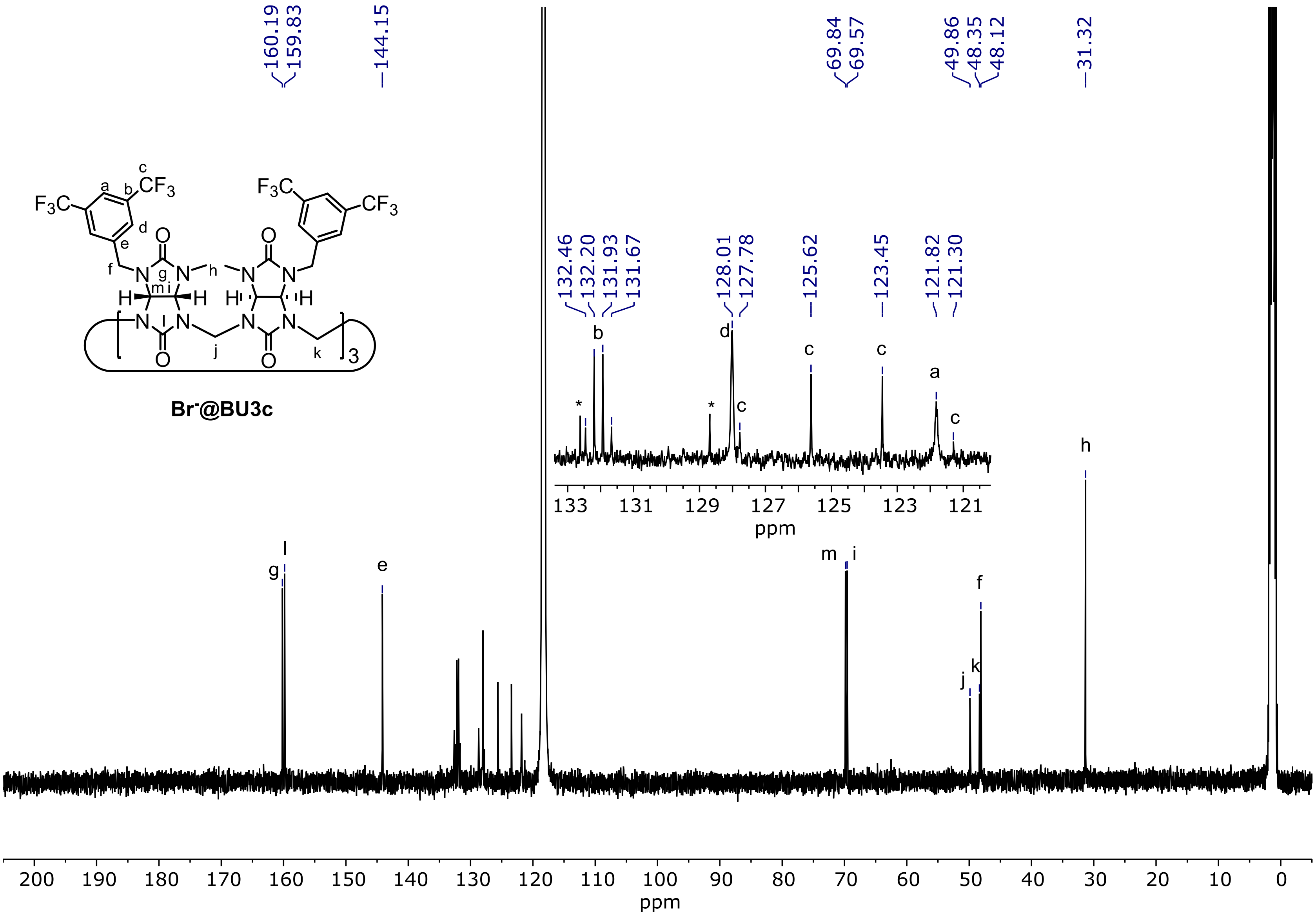

Figure S63. $^{13}C\{^1H\}$ NMR spectrum (126 MHz, CD_3CN, 303 K) of bambus[6]uril **Br⁻@BU3c** (*impurity).

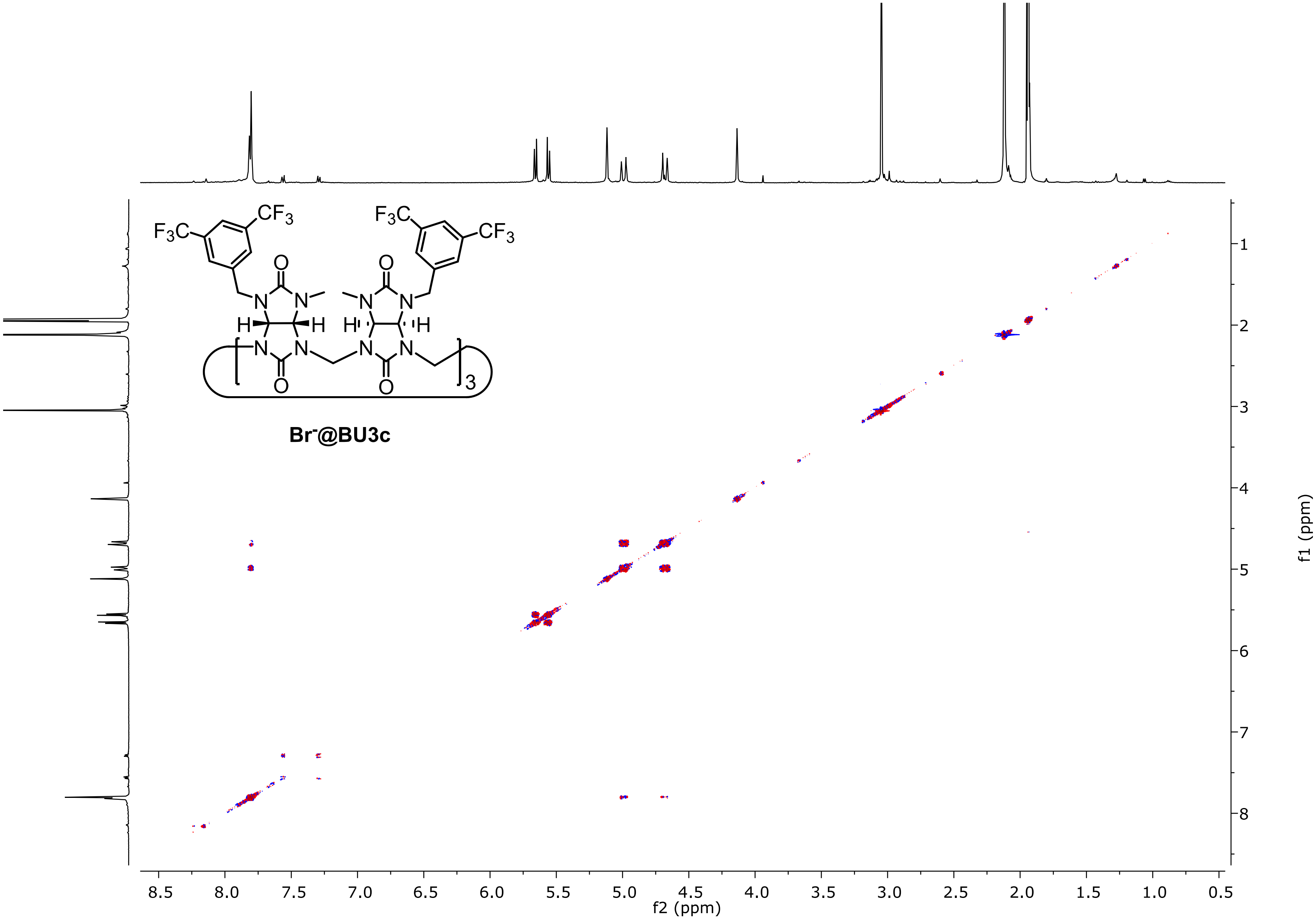

Figure S64. COSY NMR spectrum (500 MHz, CD_3CN, 303 K) of glycoluril **Br$^-$@BU3c**.

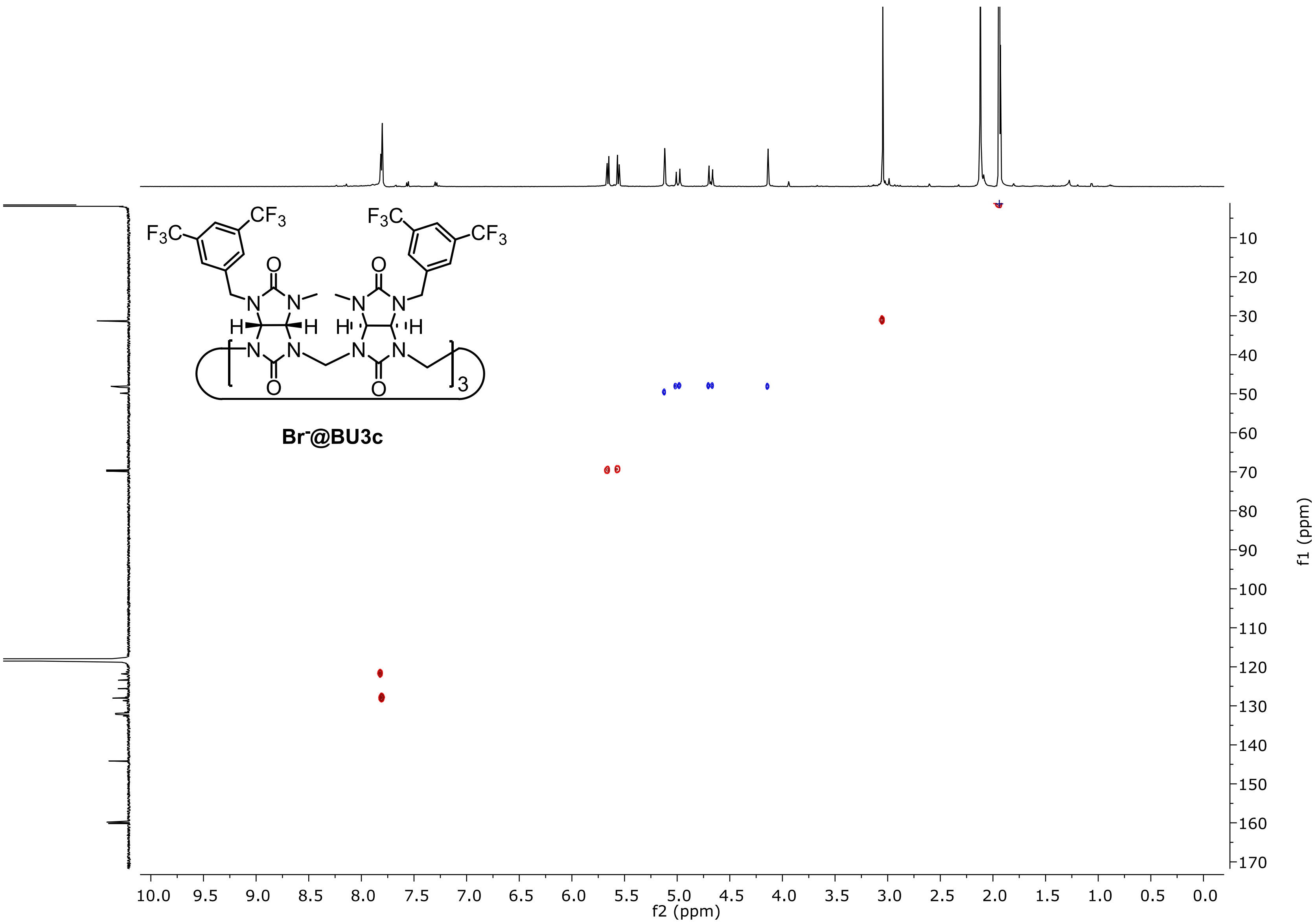

Figure S65. HSQC NMR spectrum (500 MHz, CD_3CN, 303 K) of glycoluril **Br⁻@BU3c**.

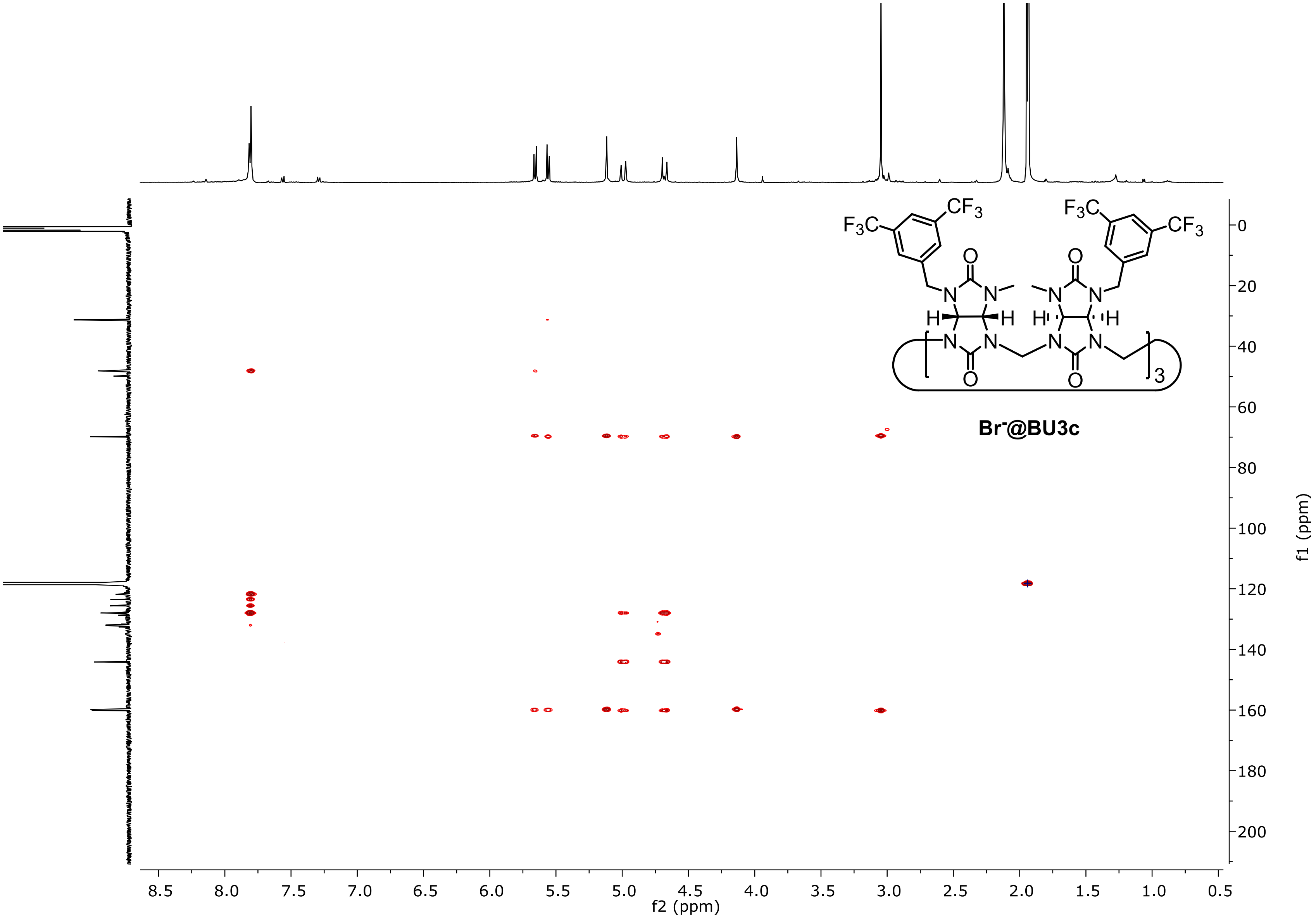

Figure S66. HMBQ NMR spectrum (500 MHz, CD_3CN, 303 K) of glycoluril **Br⁻@BU3c**.

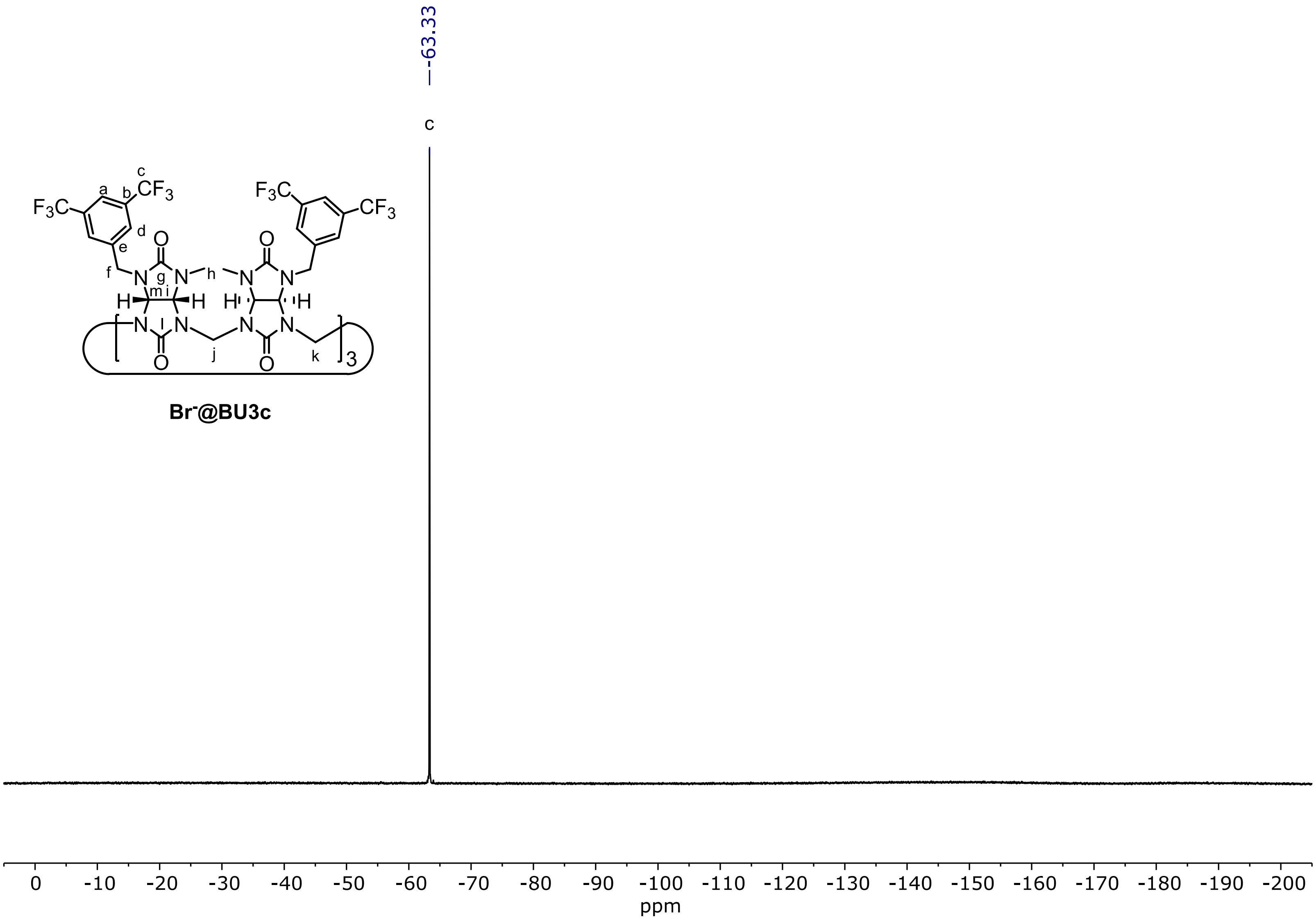

Figure S67. $^{19}F\{^{1}H\}$ NMR spectrum (282 MHz, CD_3CN, 303 K) of bambus[6]uril **Br⁻@BU3c**.

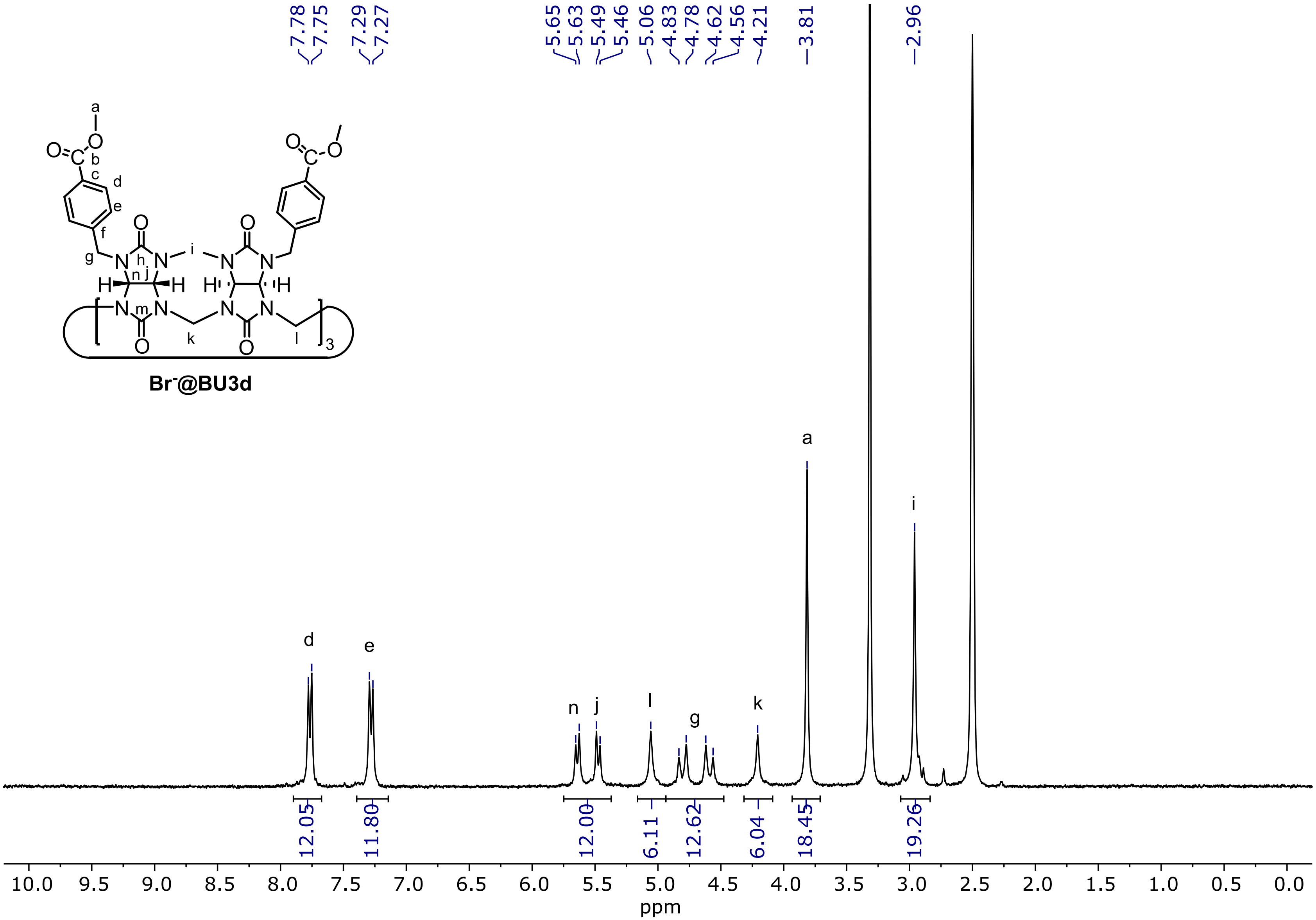

Figure S68. ^{1}H NMR spectrum (300 MHz, DMSO-d_6, 303 K) of bambus[6]uril **Br$^-$@BU3d**.

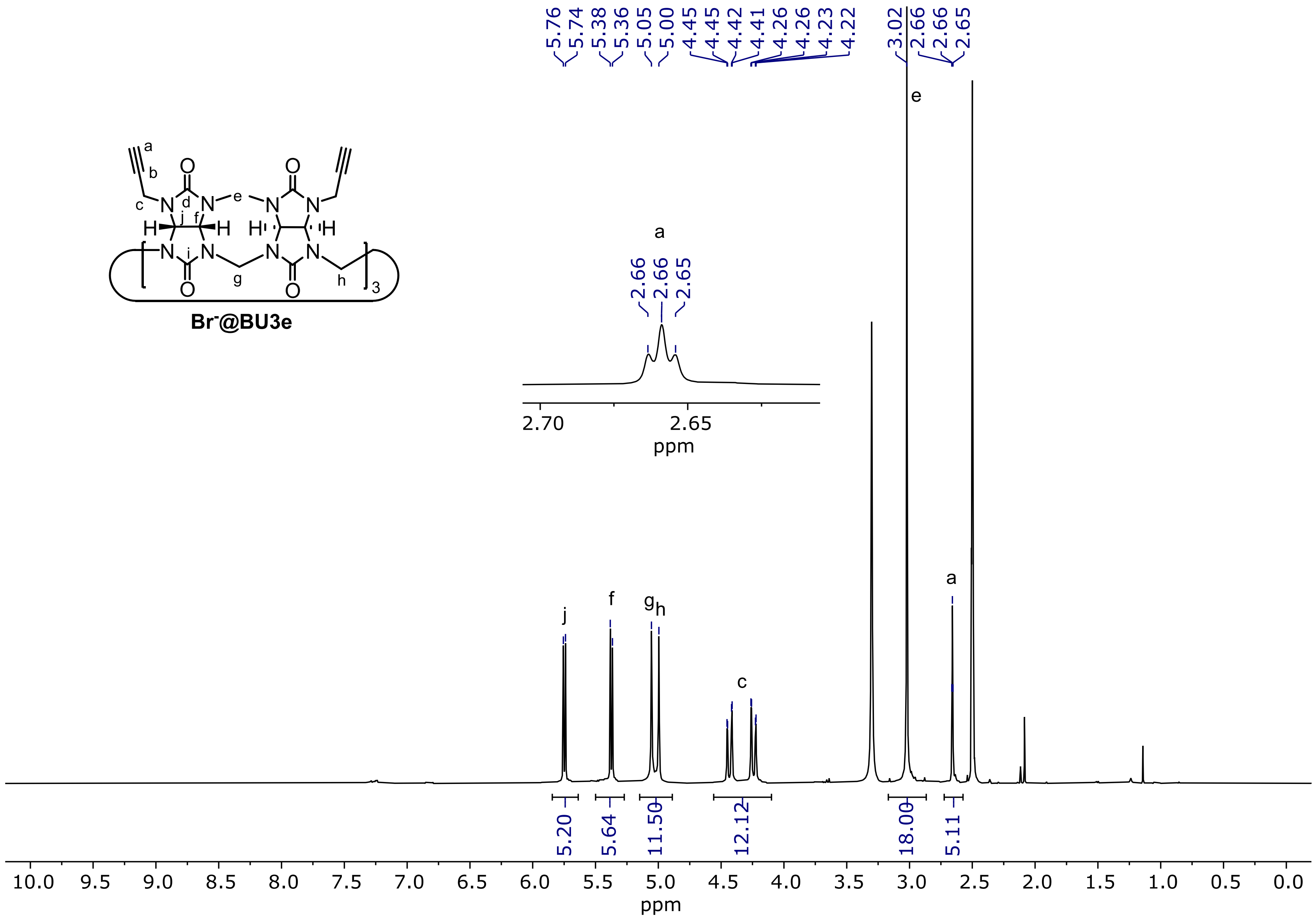

Figure S69. ^{1}H NMR spectrum (500 MHz, DMSO-d_6, 303 K) of bambus[6]uril **Br⁻@BU3e**.

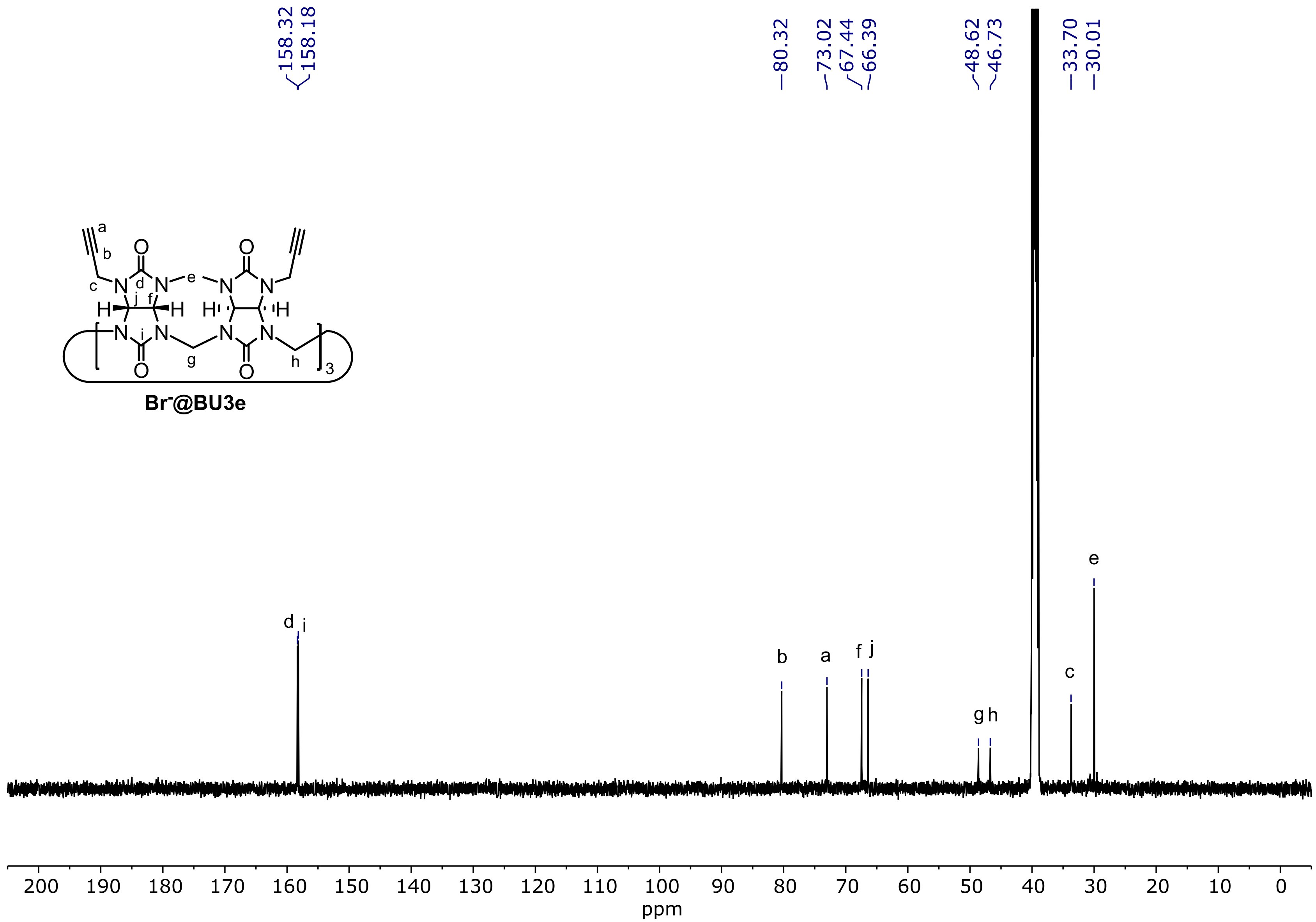

Figure S70. $^{13}C\{^{1}H\}$ NMR spectrum (126 MHz, DMSO-d_6, 303 K) of bambus[6]uril **Br⁻@BU3e**.

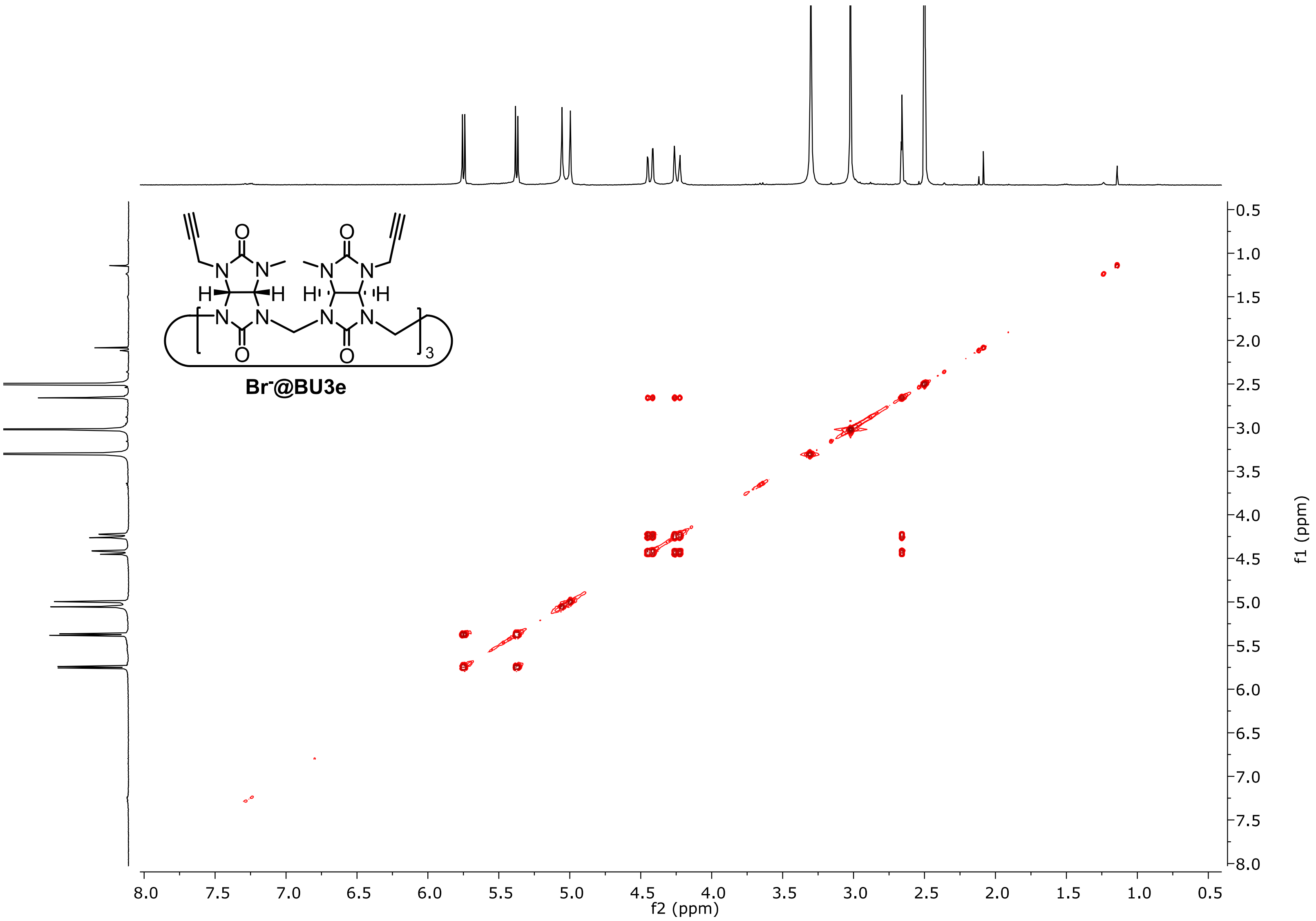

Figure S71. COSY NMR spectrum (500 MHz, DMSO-d_6, 303 K) of glycoluril **Br$^-$@BU3d**.

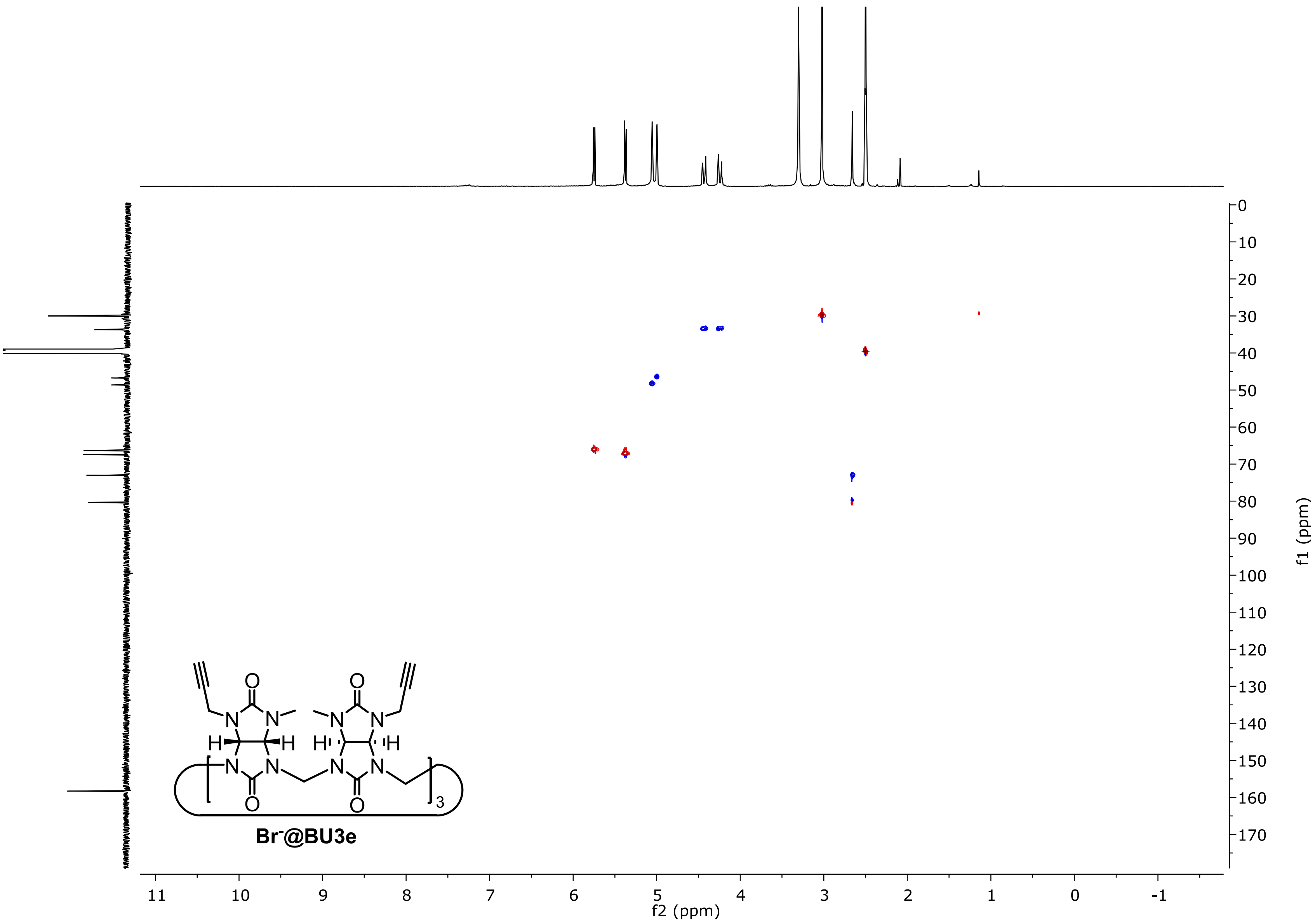

Figure S72. HSQC NMR spectrum (500 MHz, DMSO-d_6, 303 K) of glycoluril **Br⁻@BU3d**.

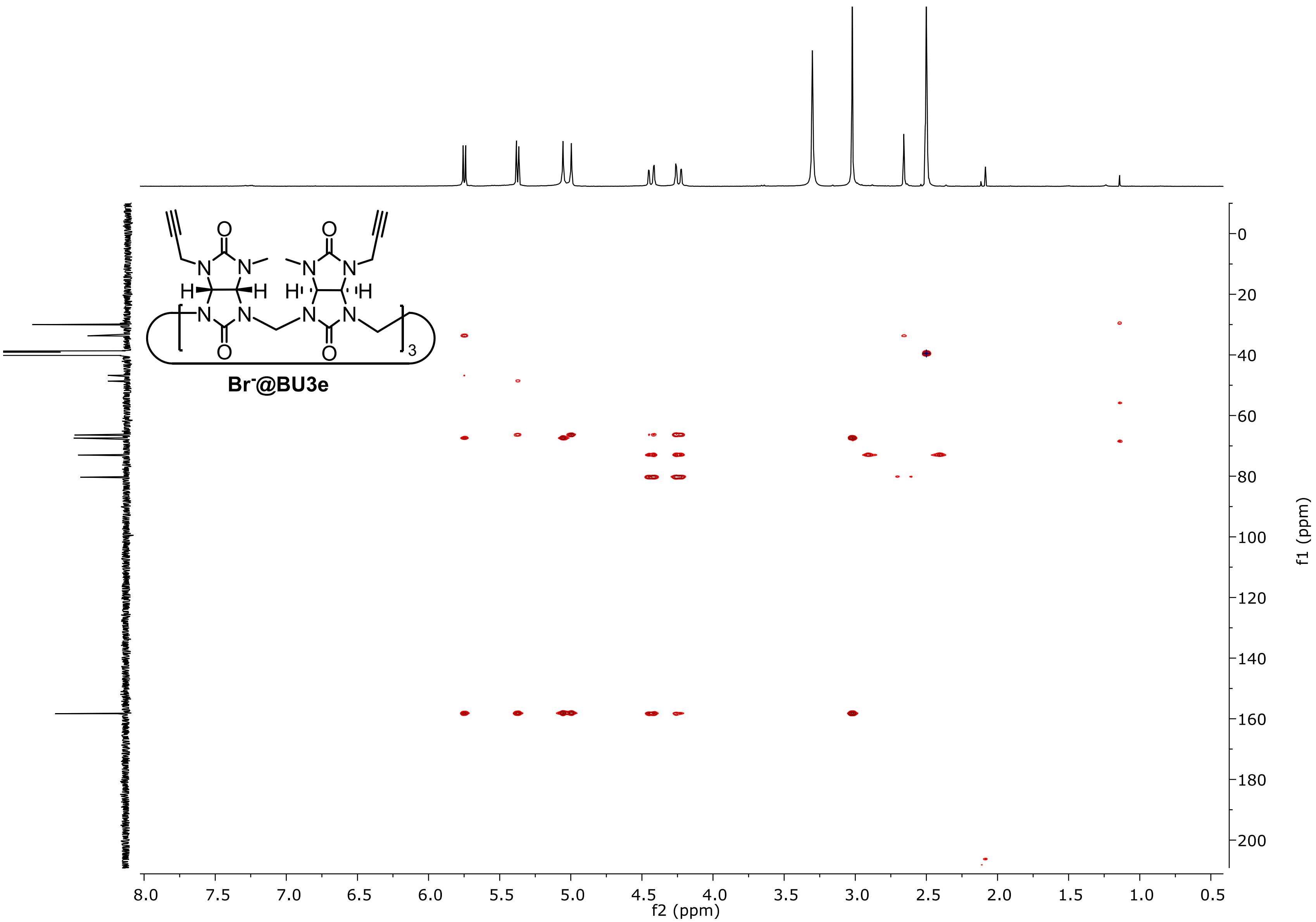

Figure S73. HMBC NMR spectrum (500 MHz, DMSO-d_6, 303 K) of glycoluril **Br$^-$@BU3d**.

3. MS spectra

APCI+ (MMI)
nitrogen flow 5 L/min, gas temperature 325°C, nebulizer 45 psig, skimmer 65 V, vaporizer 200°C, fragmentor 5 V, dissolved in dichloromethane

$C_{19}H_{24}N_4O_2$ **exact mass: 340.1899**

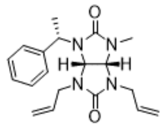

4

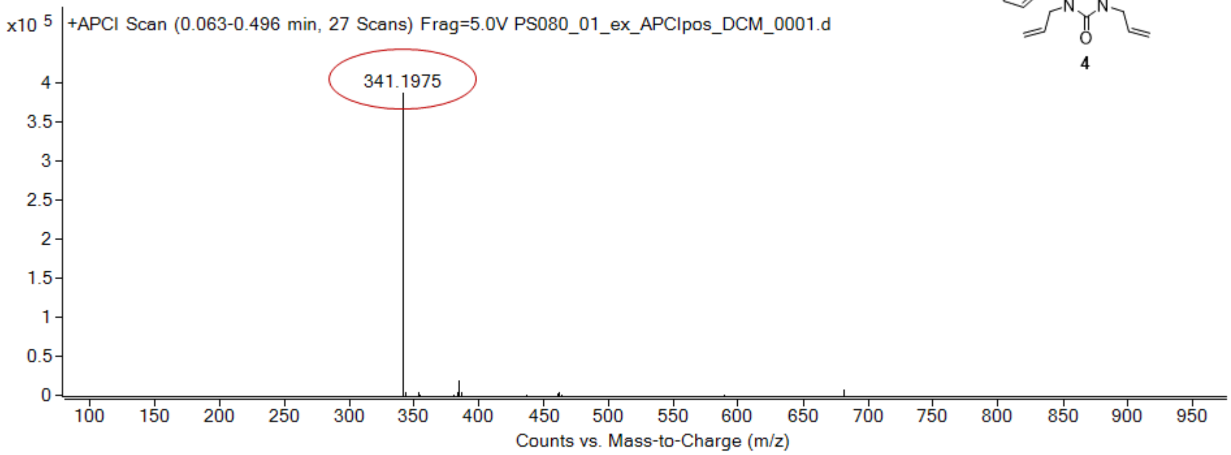

expected mass: $[M+H]^+ = 341.1972$ observed mass : $[M+H]^+ = 341.1975$ mass accuracy = 0.9 ppm

Figure S74. HRMS (APCI+) spectrum of glycoluril **4**.

APCI+ (MMI)
nitrogen flow 5 L/min, gas temperature 325°C, nebulizer 45 psig, skimmer 65 V, vaporizer 200°C, fragmentor 20 V, dissolved in methanol

$C_{11}H_{16}N_4O_2$ **exact mass: 236.1273**

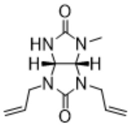

5

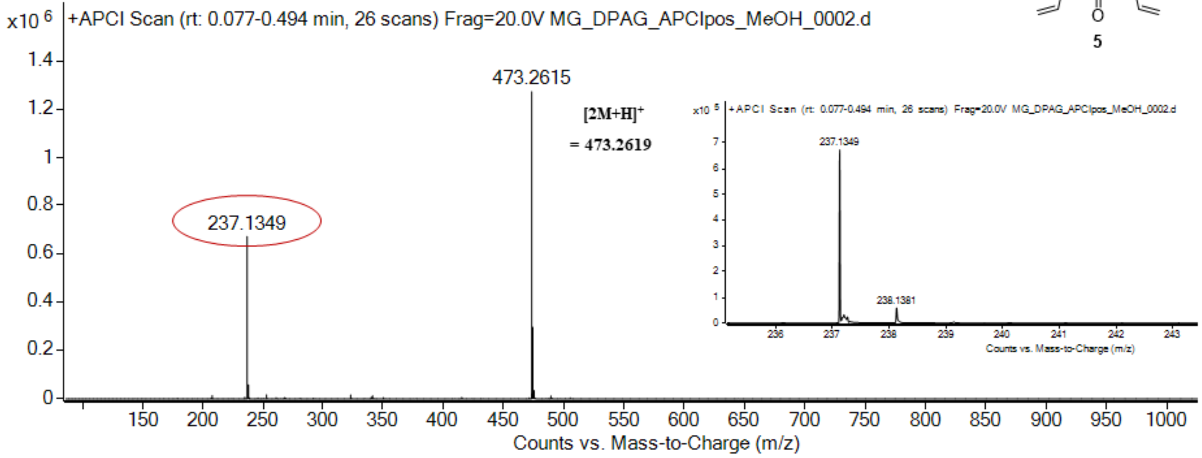

expected mass: $[M+H]^+ = 237.1346$ observed mass: $[M+H]^+ = 237.1349$ mass accuracy = 1.3 ppm

Figure S75. HRMS (APCI+) spectrum of glycoluril **5**.

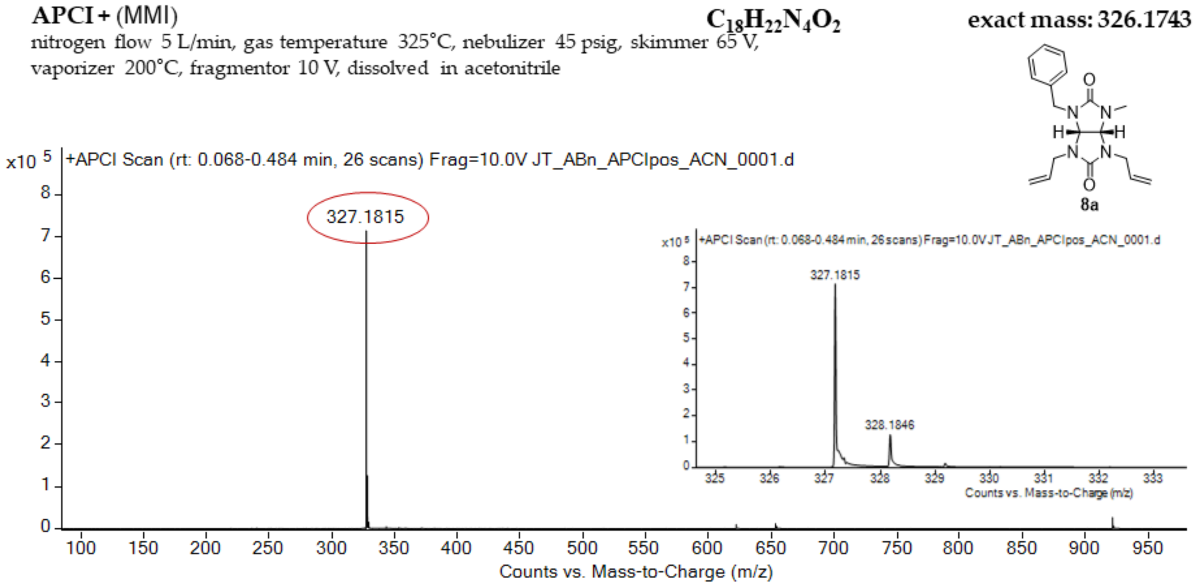

expected mass: $[M+H]^+ = 327.1816$ observed mass : $[M+H]^+ = 327.1815$ mass accuracy = - 0.3 ppm

Figure S76. HRMS (APCI+) spectrum of glycoluril **8a**.

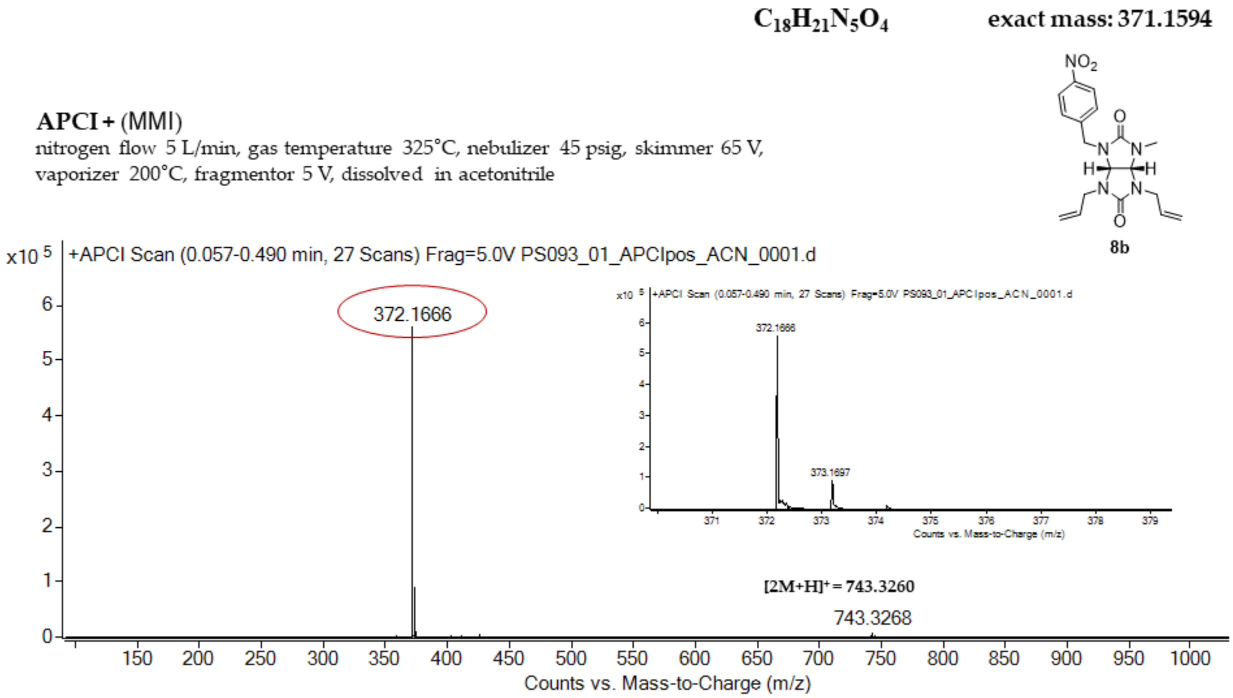

expected mass: $[M+H]^+ = 372.1666$ observed mass: $[M+H]^+ = 372.1666$ mass accuracy < 0.1 ppm

Figure S77. HRMS (APCI+) spectrum of glycoluril **8b**.

APCI + (MMI)
nitrogen flow 5 L/min, gas temperature 325°C, nebulizer 45 psig,
skimmer 65 V, vaporizer 200°C, fragmentor 5 V, dissolved in methanol

$C_{20}H_{20}F_6N_4O_2$ **exact mass: 462.1490**

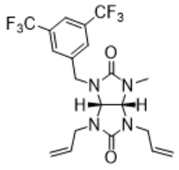

8c

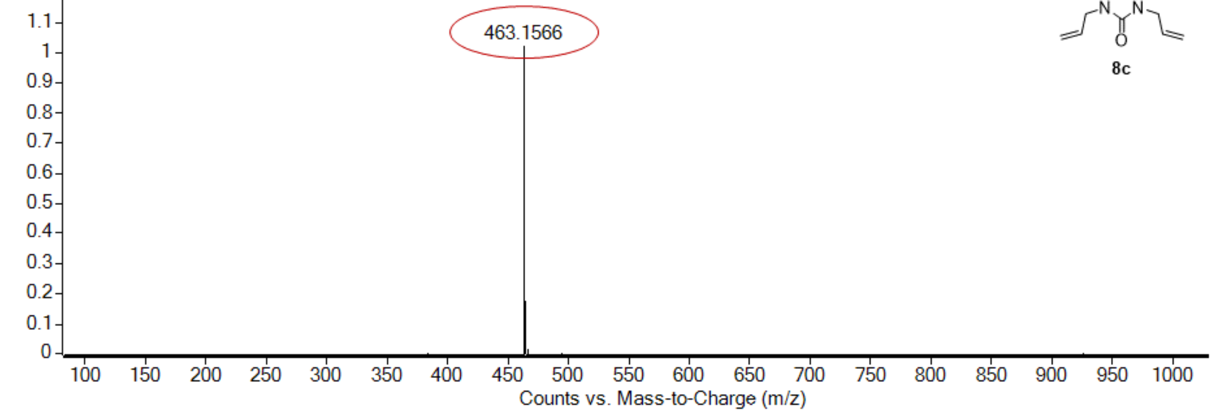

expected mass: $[M+H]^+$ = 463.1563 observed mass: $[M+H]^+$ = 463.1566 mass accuracy = 0.6 ppm

Figure S78. HRMS (APCI+) spectrum of glykoluril **8c**.

APCI + (MMI)
nitrogen flow 5 L/min, gas temperature 325°C, nebulizer 45 psig, skimmer 65 V,
vaporizer 200°C, fragmentor 20 V, dissolved in methanol

$C_{20}H_{24}N_4O_4$ **exact mass: 384.1798**

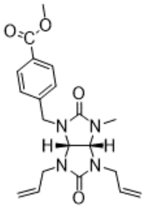

8d

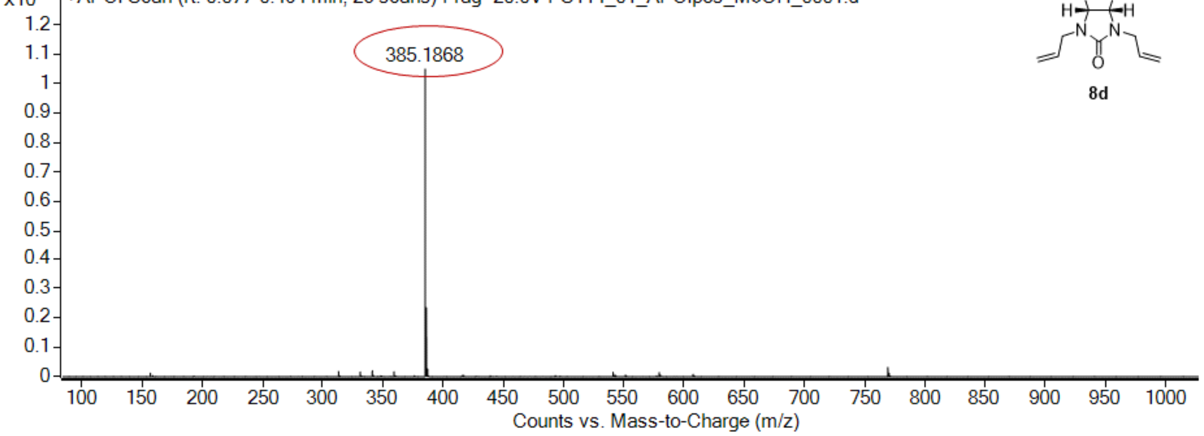

expected mass: $[M+H]^+$ = 385.1870 observed mass: $[M+H]^+$ = 385.1868 mass accuracy = - 0.5 ppm

Figure S79. HRMS (APCI+) spectrum of glycoluril **8d**.

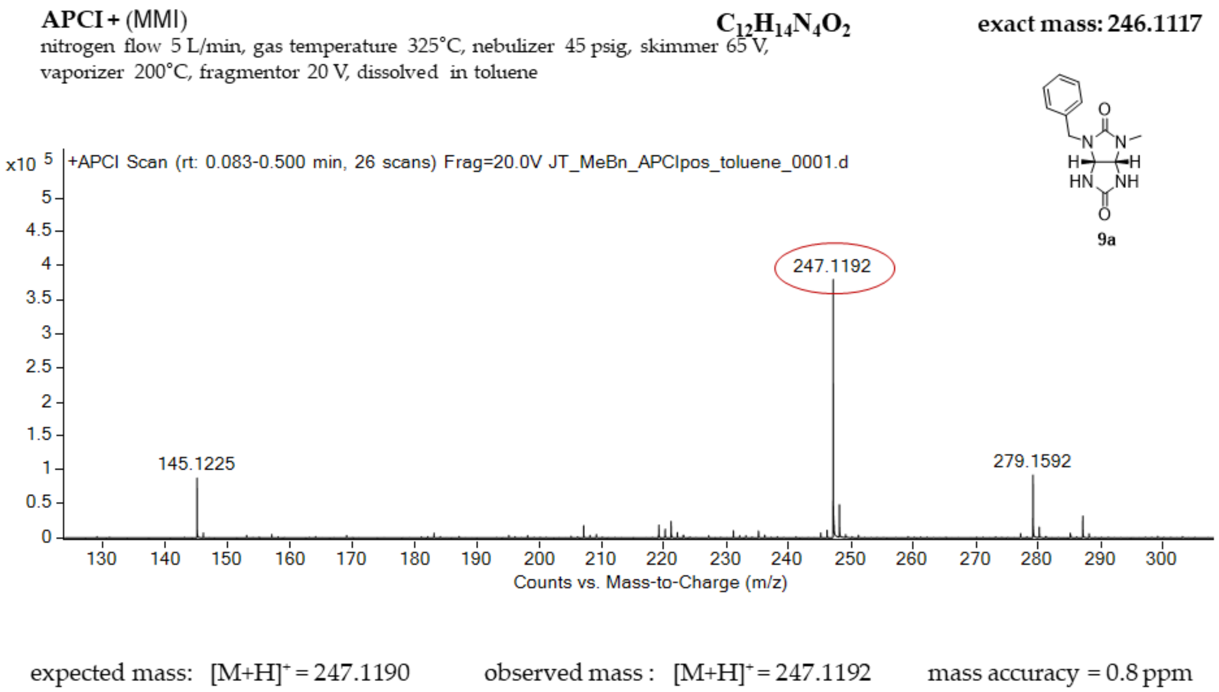

Figure S80. HRMS (APCI+) spectrum of glykoluril **9a**.

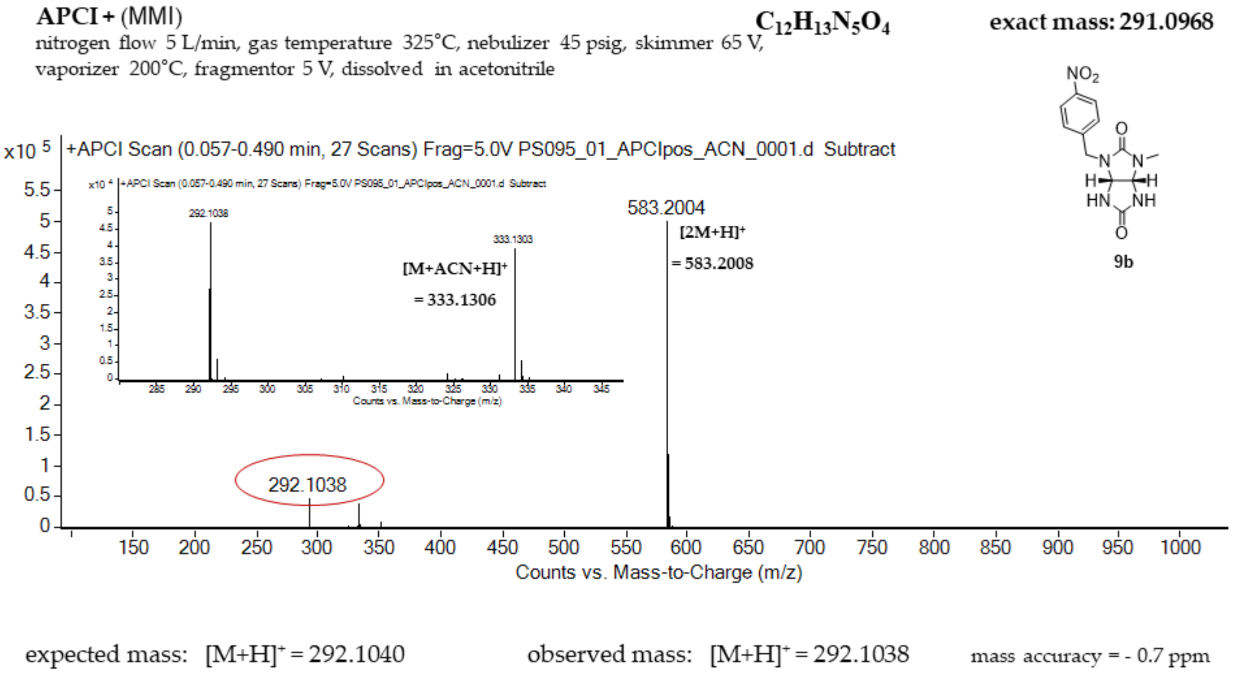

Figure S81. HRMS (APCI+) spectrum of glycoluril **9b**.

APCI+ (MMI) **$C_{14}H_{12}F_6N_4O_2$** **exact mass: 382.0864**

nitrogen flow 5 L/min, gas temperature 325°C, nebulizer 45 psig, skimmer 65 V, vaporizer 200°C, fragmentor 15 V, dissolved in methanol

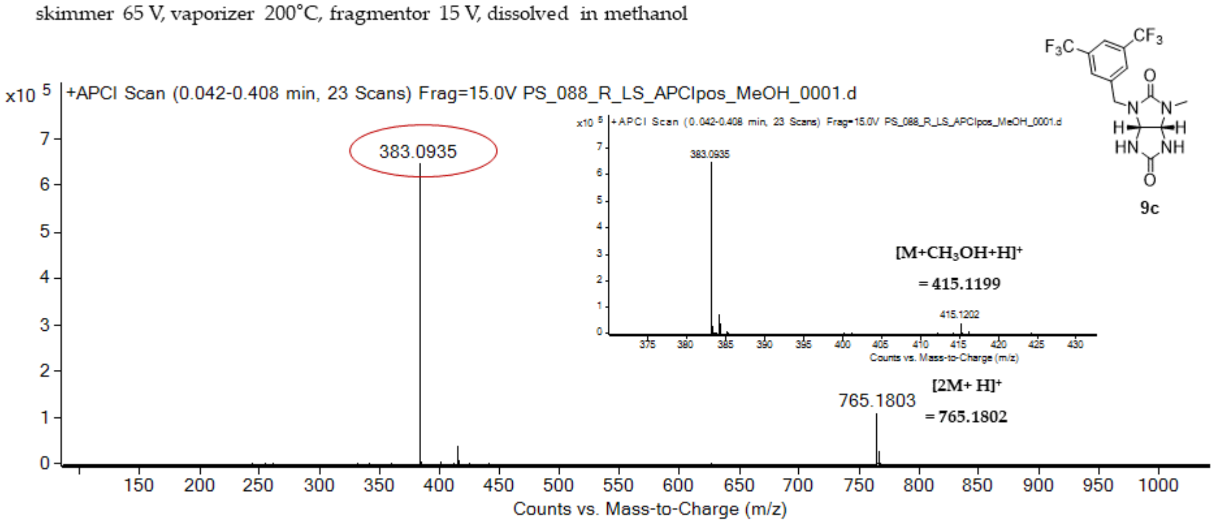

expected mass: $[M+H]^+$ = 383.0937 observed mass: $[M+H]^+$ = 383.0935 mass accuracy = - 0.5 ppm

Figure S82. HRMS (APCI+) spectrum of glycoluril **9c**.

APCI+ (MMI) **$C_{14}H_{16}N_4O_4$** **exact mass: 304.1172**

nitrogen flow 5 L/min, gas temperature 325°C, nebulizer 45 psig, skimmer 65 V, vaporizer 200°C, fragmentor 15 V, dissolved in methanol

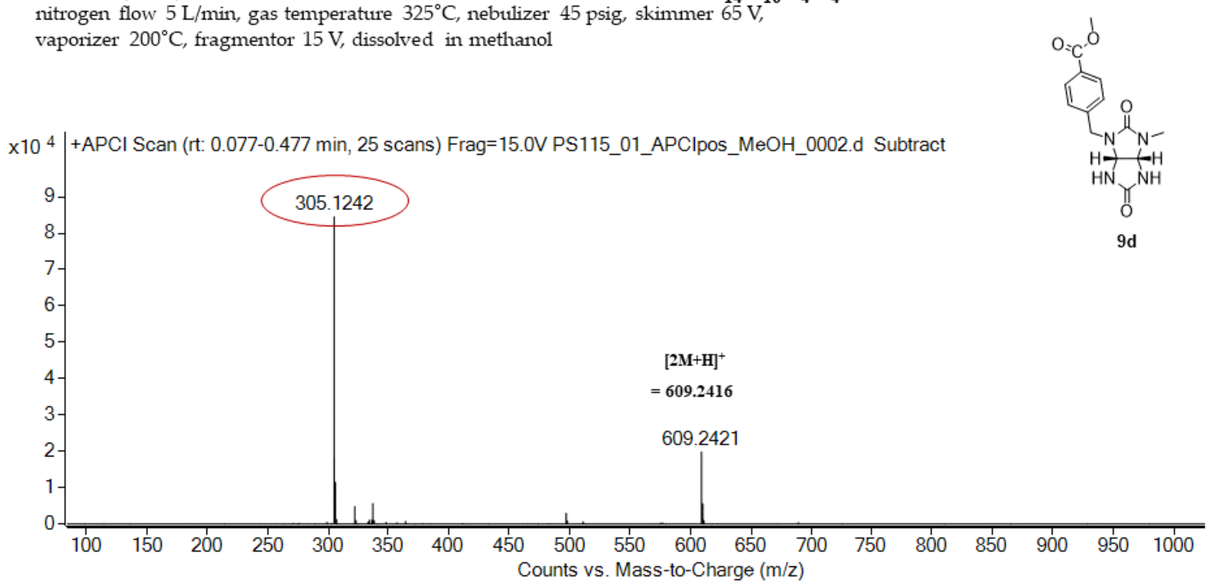

expected mass: $[M+H]^+$ = 305.1244 observed mass: $[M+H]^+$ = 305.1242 mass accuracy = - 0.7 ppm

Figure S83. HRMS (APCI+) spectrum of glycoluril **9d**.

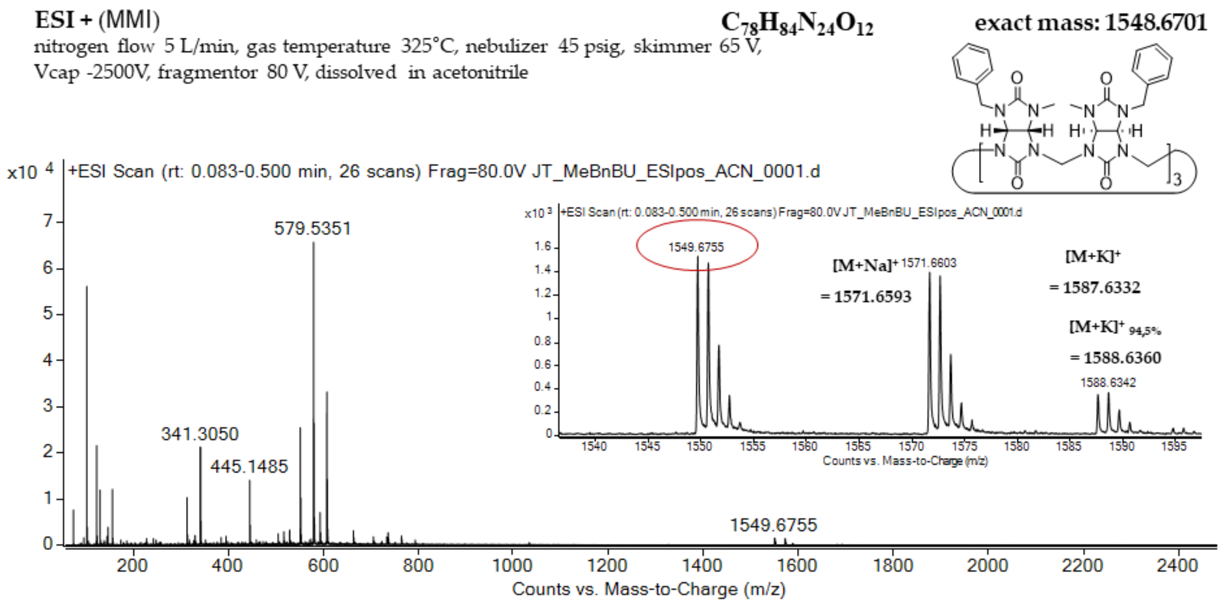

expected mass: $[M+H]^+$ = 1549.6773 observed mass : $[M+H]^+$ = 1549.6755 mass accuracy = - 1.2 ppm

Figure S84. ESI(+) MS spectrum of bambus[6]uril **HSO_4^-@BU3a** obtained *via* macrocyclization.

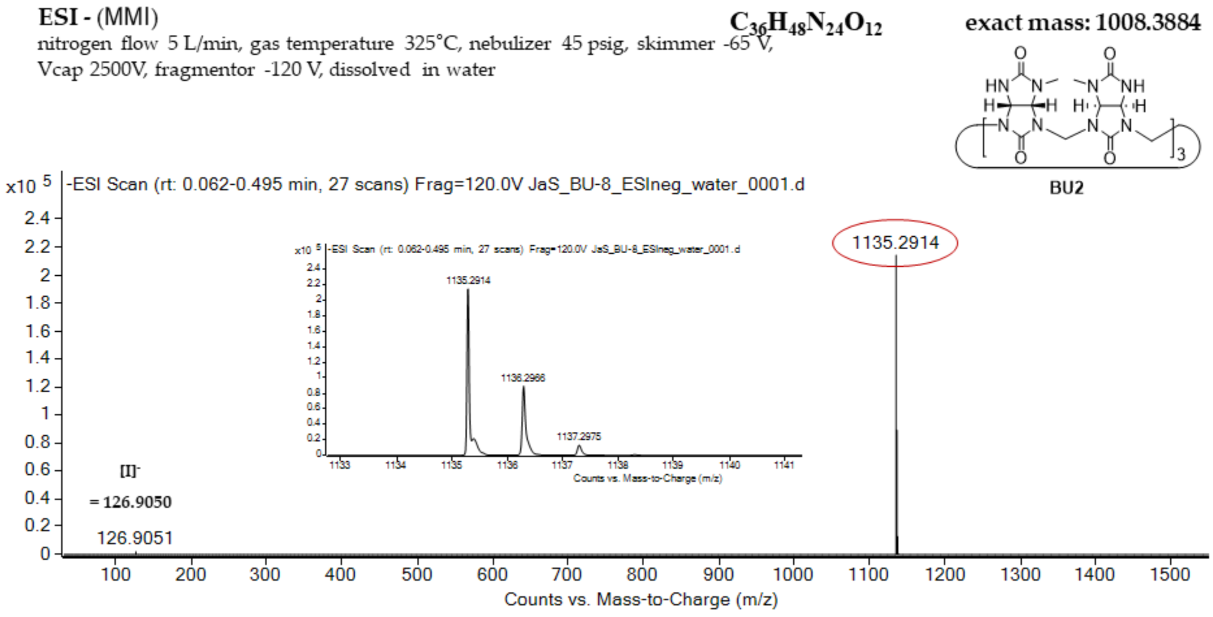

expected mass: $[M+I]^-$ = 1135.2934 observed mass : $[M+I]^-$ = 1135.2914 mass accuracy = - 1.8 ppm

Figure S85. ESI(-) MS spectrum of bambus[6]uril **BU2**.

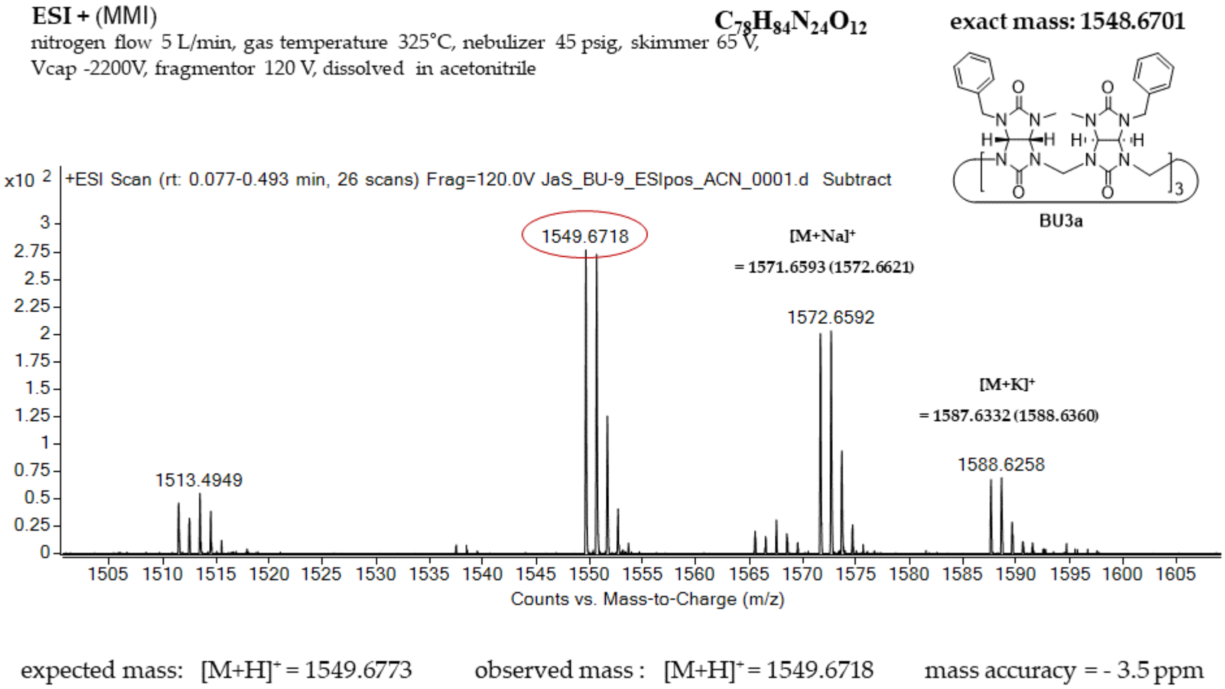

Figure S86. ESI(+) MS spectrum of bambus[6]uril **BU3a** obtained via alkylation.

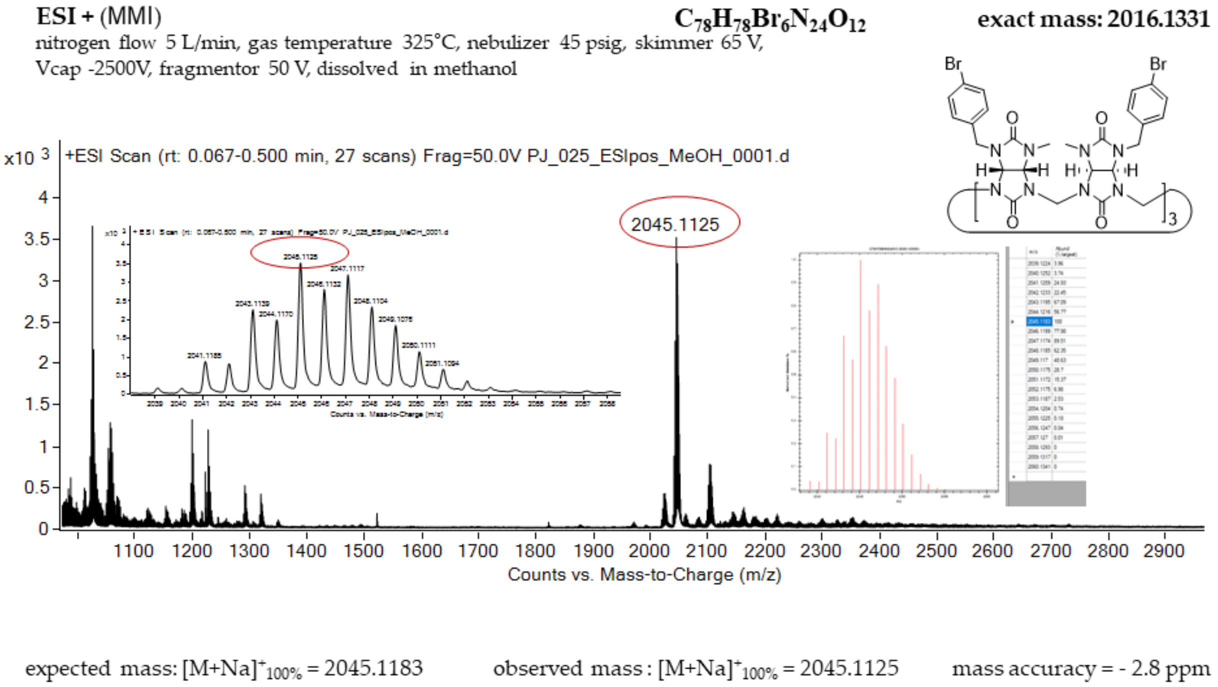

Figure S87. ESI(+) MS spectrum of bambus[6]uril **Br$^-$@BU3b**.

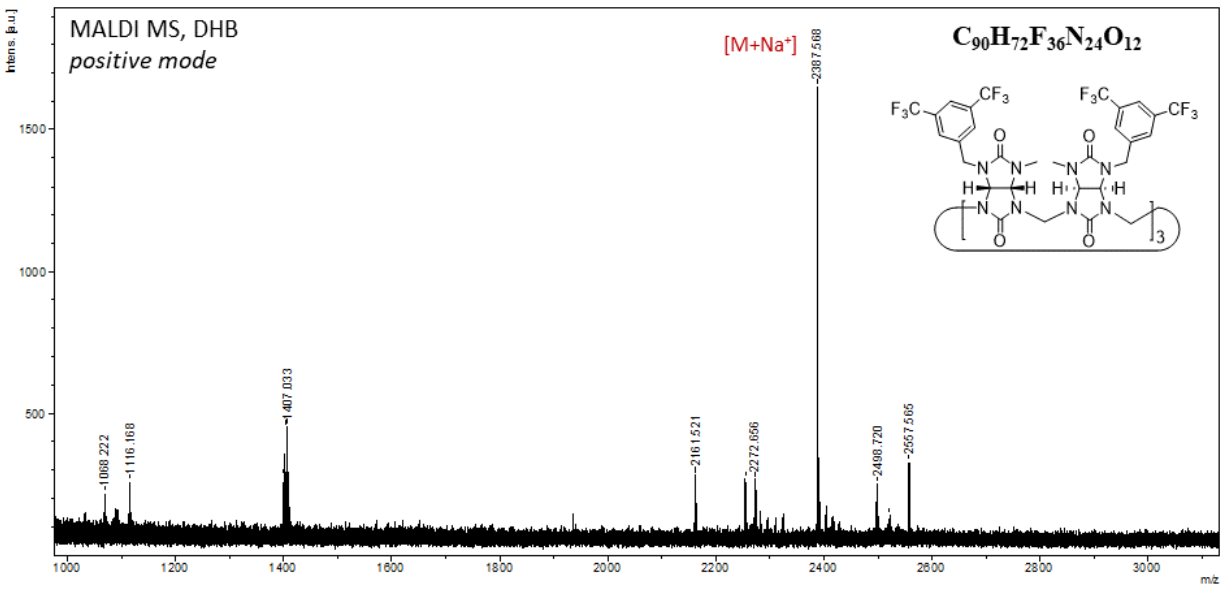

Figure S88. MALDI-TOF(+) MS spectrum of bambus[6]uril **Br^{-}@BU3c**.

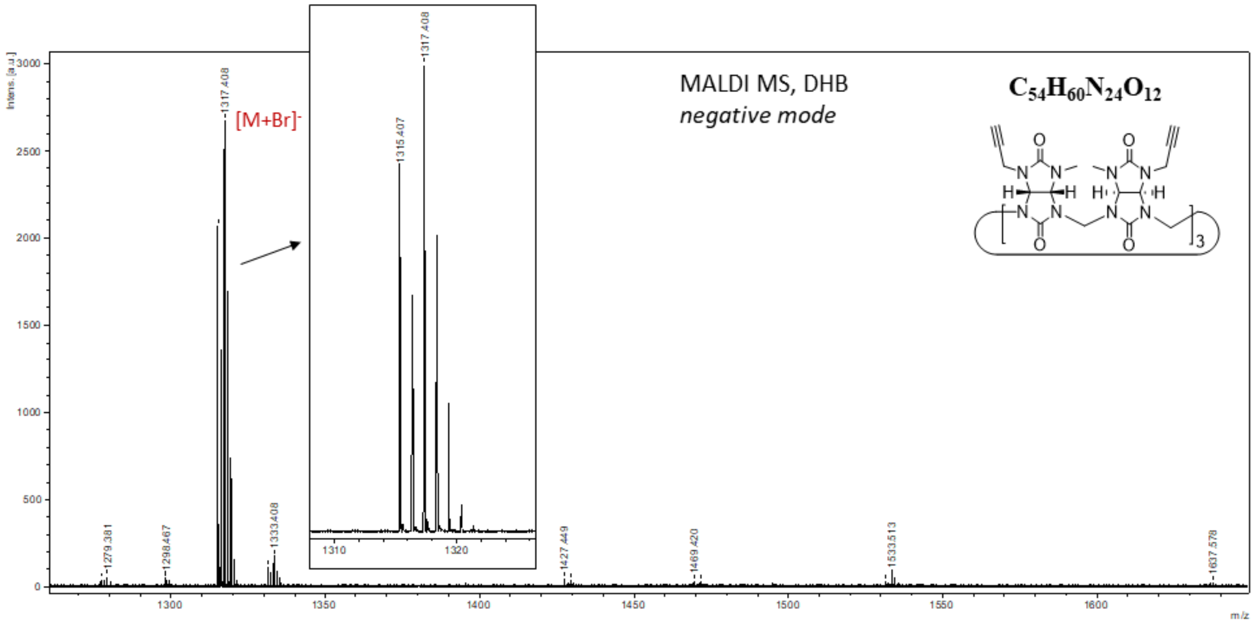

No relevant signals were observed outside the displayed m/z range.

Figure S89 MALDI-TOF(-) MS spectrum of bambus[6]uril **Br^{-}@BU3e**.

4. References

(1) Padiya, K. J.; Gavade, S.; Kardile, B.; Tiwari, M.; Bajare, S.; Mane, M.; Gaware, V.; Varghese, S.; Harel, D.; Kurhade, S. Unprecedented “In Water” Imidazole Carbonylation: Paradigm Shift for Preparation of Urea and Carbamate. *Org. Lett.* **2012**, *14* (11), 2814–2817. https://doi.org/10.1021/ol301009d.

(2) Paik, S.; Lee, J. Y. Simple and Efficient Cleavage of the *N*-(1-Phenylethyl) Unit of Carboxamides with Methanesulfonic Acid. *Tetrahedron Lett.* **2006**, *47* (11), 1813–1815. https://doi.org/10.1016/j.tetlet.2006.01.019.

(3) Zacuto, M. J.; Xu, F. One-Step RhCl3-Catalyzed Deprotection of Acyclic *N*-Allyl Amides. *J. Org. Chem.* **2007**, *72* (16), 6298–6300. https://doi.org/10.1021/jo070553t.

(4) Cadierno, V.; Gimeno, J.; Nebra, N. Efficient Tandem Process for the Catalytic Deprotection of *N*-Allyl Amides and Lactams in Aqueous Media: A Novel Application of the Bis(Allyl)–Ruthenium(IV) Catalysts [Ru(H3:H2:H3-C12H18)Cl2] and [Ru(H3:H3-C10H16)(μ-Cl)Cl2]. *Chem. Eur. J.* **2007**, *13* (23), 6590–6594. https://doi.org/10.1002/chem.200700477.

(5) Ohmura, N.; Nakamura, A.; Hamasaki, A.; Tokunaga, M. Hydrolytic Deallylation of *N*-Allyl Amides Catalyzed by PdII Complexes. *Eur. J. Org. Chem.* **2008**, *2008* (30), 5042–5045. https://doi.org/10.1002/ejoc.200800771.
